# Integrative simulation analysis of myocardial ischaemia–reperfusion injury

**DOI:** 10.1113/JP290440

**Published:** 2026-08-20

**Authors:** Takao Shimayoshi, Ayako Takeuchi, Satoshi Matsuoka

**Affiliations:** ^1^ Center for Artificial Intelligence and Mathematical Data Science Okayama University Okayama Japan; ^2^ Department of Physiology and Biophysics, Faculty of Medicine, Dentistry and Pharmaceutical Sciences Okayama University Okayama Japan; ^3^ Department of Integrative and Systems Physiology, Faculty of Medical Sciences University of Fukui Fukui Japan; ^4^ Life Science Innovation Center University of Fukui Fukui Japan

**Keywords:** computational modelling, heart, ischaemia, mitochondria, reperfusion

## Abstract

**Abstract:**

Cardiac ischaemia, reduced coronary blood flow, causes significant damages to the heart, and paradoxically reperfusion, restored flow after ischaemia, often results in more severe injury. Although the mechanisms underlying cardiac ischaemia–reperfusion injury have been intensively investigated, effective therapeutic strategies applicable during reperfusion to mitigate this damage have not been established yet. To obtain more insights into the mechanisms underlying ischaemia–reperfusion injury, and to explore effective methods to relieve reperfusion injury, we developed a comprehensive mathematical model of ventricular myocyte, implementing excitation–contraction (E–C) coupling, cytosolic and mitochondrial pH regulation, pH dependence of major components of E–C coupling and cellular energy metabolism, including mitochondrial oxidative phosphorylation. The mathematical model successfully reproduced experimentally observed findings on cardiac ischaemia–reperfusion injury, such as intracellular and extracellular acidosis, cytosolic Na^+^ overload, cytosolic Ca^2+^ overload, mitochondrial dysfunction, action potential shortening and contraction failure. It was found that the reperfusion‐induced exacerbation of Na^+^_i_, Ca^2+^_i_ overloads was mainly caused by insufficient production of NADH, which is used in mitochondrial oxidative phosphorylation to synthesize ATP for Na^+^/K^+^ pump and sarcoplasmic/endoplasmic reticulum Ca^2+^ pump (SERCA) to expel accumulated cytosolic Na^+^ and Ca^2+^, respectively. It was suggested that the preservation of mitochondrial NADH production, attenuation of ATP consumption via SERCA or contraction for a short period or activation of the Na^+^/K^+^ pump during reperfusion mitigates reperfusion injury.

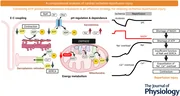

**Key points:**

- Experimental data on cardiac ischaemia–reperfusion injury are available.
- No effective methods applicable during reperfusion were available for mitigating the injury.
- We developed a comprehensive cardiomyocyte model to simulate the injury.
- Na^+^ and Ca^2+^ overloads and contraction failure could be reproduced by the model.
- It was found that correcting ATP supply–consumption imbalance during reperfusion, that is, preservation of mitochondrial NADH production, attenuation of ATP consumption or activation of the Na^+^/K^+^ pump, was effective at mitigating the injury.

## Graphical abstract

**Abstract figure legend** A comprehensive mathematical model of ventricular myocyte was developed to gain insights into the mechanisms underlying cardiac ischaemia–reperfusion injury. The model integrates excitation–contraction (E–C) coupling, cytosolic and mitochondrial pH regulation, pH dependence of major E–C coupling components and cellular energy metabolism, including mitochondrial oxidative phosphorylation (OXPHOS). During ischaemia, cytosolic acidosis develops, ATP depletes and NADH increases. Reperfusion restores oxygen  supply and cytosolic pH (pH_i_). However mitochondrial ATP production remains insufficient despite near‐complete depletion of NADH, leading to ATP shortage. This in turn results in insufficient function of the ATP‐dependent Na^+^/K^+^ pump (NaK) and sarcoplasmic/endoplasmic reticulum Ca^2+^ pump (SERCA), exacerbating Na^+^ and Ca^2+^ overloads, namely the hallmark of reperfusion injury. Our simulation analyses propose that correcting ATP production–utilization imbalance is an effective strategy for relieving reperfusion injury.

## Introduction

Cardiac ischaemia, reduction in coronary blood flow to a region of the heart, causes electrophysiological and mechanical damage to the heart, that is, arrhythmia and contractile dysfunction. Furthermore reperfusion, restart of coronary flow after ischaemia, often induces paradoxically more severe damage. The series of cardiac reactions leading to serious cardiac dysfunction is called ischaemia–reperfusion injury. The mechanisms have been extensively studied, but a comprehensive understanding is still lacking and effective treatments to reduce ischaemia–reperfusion injury have not been established (Baumeister & Quinn, 2016; Bertero et al., 2024; Carmeliet, 1999; Murphy & Steenbergen, 2008). Generally the following disturbances have been recognized during ischaemia and reperfusion. Ischaemia induces intracellular acidosis and activates sarcolemmal Na^+^‐H^+^ exchange (NHE), and then elevates cytosolic Na^+^ (Na^+^_i_) and subsequently increases cytosolic Ca^2+^ (Ca^2+^_i_) due to attenuation of Ca^2+^ efflux via sarcolemmal Na^+^‐Ca^2+^ exchange (NCX). Extracellular K^+^ (K^+^_o_) accumulates in the interstitial space and causes membrane depolarization. Insufficient supply of oxygen to cardiomyocytes depolarizes mitochondrial membrane potential (ΔΨ) and suppresses mitochondrial ATP synthesis in the mitochondrial oxidative phosphorylation (OXPHOS) process, and mitochondrial Ca^2+^ overload activates the mitochondrial permeability transition pore (mPTP), resulting in further depolarization of ΔΨ. Disturbed balance of energy metabolism induces decreases in phosphocreatine (PCr) and ATP, and an increase in inorganic phosphate (Pi). Finally the heart stops contraction and action potential generation. If coronary flow restarts (reperfusion), intracellular acidosis may recover after a period of time, but more severe damage is often induced partially because of mitochondrial dysfunction, excessive reactive oxygen species (ROS) production and activation of harmful signalling pathways. Acidosis and the resulting Na^+^_i_ overload have been recognized as crucial factors of ischaemia–reperfusion injury. In fact treatment of the heart with cariporide, an inhibitor of NHE, attenuated ischaemia–reperfusion injury when applied during the ischaemia period. However cariporide had no beneficial effects when applied during the early reperfusion period (Klein et al., 2000), and a clinical trial (Theroux et al., 2000) failed to show an overall clinical benefit of cariporide. Therefore a more comprehensive understanding of and an integrative approach to cardiac ischaemia–reperfusion injury are required.

Computational modelling analysis is a powerful tool to piece together fragmented experimental and clinical findings on cardiac ischaemia–reperfusion injury, and to obtain mechanistical insights into the injury, even though the complete scenario of the injury is still unclear. Ch'en et al. (1998) first developed a mathematical model of ventricular myocyte integrating excitation–contraction (E–C) coupling, cytosolic pH (pH_i_) regulation, pH_i_ dependence of contraction and cellular energy metabolism. They successfully simulated ischaemia–reperfusion injury, such as Na^+^_i_ overload, Ca^2+^_i_ overload and arrhythmias. However this model cannot evaluate the roles of OXPHOS and mPTP, which play important roles in ischaemia–reperfusion injury. Later Crampin and Smith (2006) developed an integrative ventricular cell model, in which pH_i_ regulation and dependence were improved, and successfully reproduced the responses of ventricular myocyte to acidosis. Roberts and Christini (2011, 2012) extended Crampin and Smith's model to simulate ischaemia–reperfusion injury and succeeded in reproducing the beneficial effects of NHE inhibition during ischaemia. However these models did not still include OXPHOS and mPTP. Recently Grass et al. (2022) developed a model of cardiomyocyte metabolism to simulate ischaemia–reperfusion injury, and explored optimal reperfusion strategies to minimize tissue damage from reperfusion injury. Although glycolysis and OXPHOS were included, this model lacked E–C coupling and pH_i_ regulation and dependence. Thus far no comprehensive cardiomyocyte model, which includes E–C coupling, pH_i_ regulation and dependence and cellular energy metabolism, has been developed. Such a comprehensive mathematical model should be useful in exploring strategies to mitigate ischaemia–reperfusion injury because it is, as mentioned earlier, a complex process involving the combination of multiple reactions.

Here we developed a comprehensive mathematical model of ventricular myocyte implementing E–C coupling, pH_i_ regulation, pH_i_ dependence of major components of E–C coupling and cellular energy metabolism, including OXPHOS. Our mathematical model successfully reproduced experimentally observed findings on cardiac ischaemia–reperfusion injury. We explored effective interventions which are applicable during reperfusion to mitigate reperfusion‐induced injury. It was demonstrated that the preservation of mitochondrial NADH production, attenuation of ATP consumption and activation of Na^+^/K^+^ pump (NaK) are effective ways to relieve ischaemia–reperfusion injury.

## Methods

### Computational integration of cardiac E–C coupling, energetics and pH regulation

**Figure 1:**
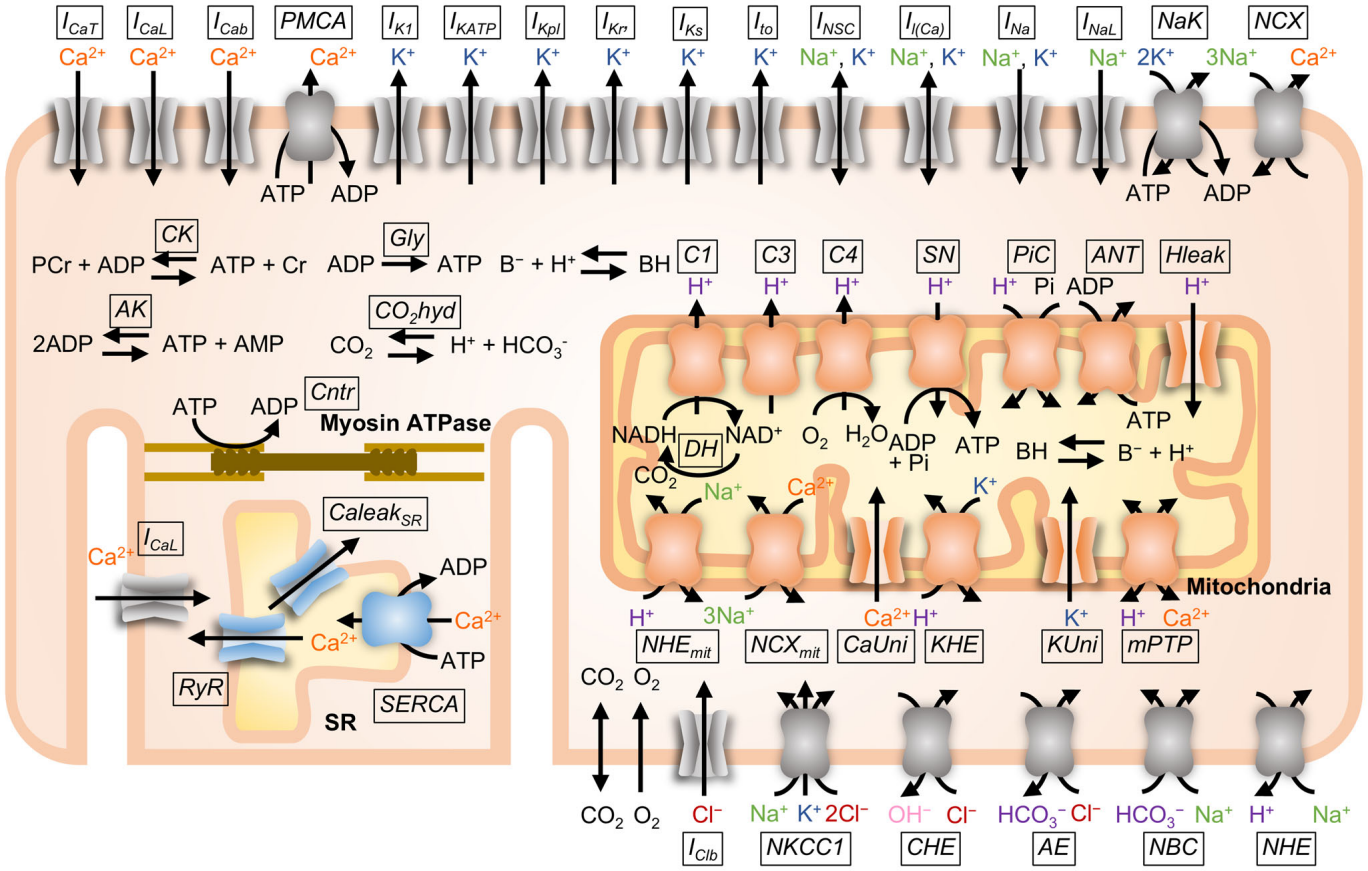
A scheme of the comprehensive mathematical model of ventricular myocyte The model includes excitation‐contraction coupling, cytosolic pH (pH_i_) regulation, pH_i_ dependence and cellular energy metabolism, including mitochondrial oxidative phophorylation. Only major factors are presented. See abbreviations in Appendix and Supplementary Material for details.

Figure 1 shows a scheme of an integrated mathematical model of cardiac excitation‐contraction‐energetics coupling. We expanded our previously published mathematical models of excitation‐contraction‐energetics coupling of guinea‐pig ventricular myocyte (Kuzumoto et al., 2008; Matsuoka et al., 2004) by implementing pH_i_ regulation, pH_i_ dependence of major components of E–C coupling and mitochondrial ion transporters. β1‐Adrenergic signalling pathway and cell volume regulation in Kuzumoto et al.’s (2008) model were omitted for simplicity. NaK model was replaced with the model developed by Oka et al. (2010) to obtain more accurate characteristics of NaK. The experimental data were primarily derived from experiments using guinea‐pig hearts, although data on the hearts of other animal species were also utilized. All equations are presented in Supplementary Material. Simulations were performed on a Windows PC using C program (Microsoft Visual Studio 2019). Ordinary differential equations were solved using CVODE (version 3.1.2; Gardner et al., 2022). Source code is available at https://github.com/atakeuti/TS‐AT‐SM‐2026‐IRmodel.

### Modelling pH regulation and dependence

**Figure 2:**
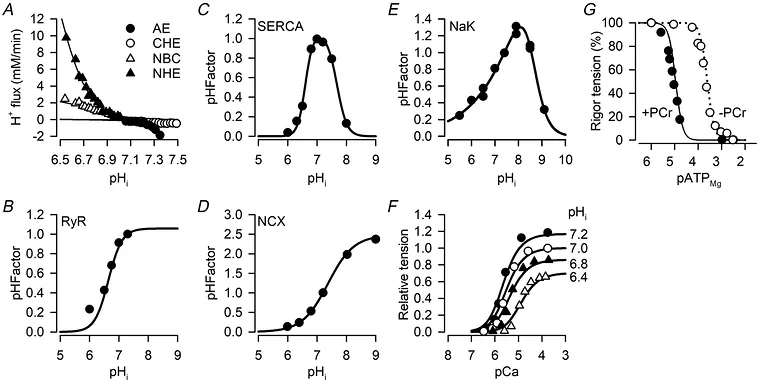
pH_i_ dependence of pH_i_‐regulating transporters and ATP_Mg_ dependence of contraction *A*, fluxes of sarcolemmal transporters that regulate cytosolic pH; Cl^−^‐HCO_3_^−^ exchange (anion exchange (AE)), Cl^−^‐OH^−^ exchange (CHE), Na^+^‐HCO_3_^−^ cotransporter (NBC) and Na^+^‐H^+^ exchange (NHE). pH_i_ dependences of RyR (ryanodine receptor) (*B*), SERCA (sarcoplasmic/endoplasmic reticulum Ca^2+^ pump) (*C*), NCX (Na^+^‐Ca^2+^ exchange) (*D*), NaK (Na^+^/K^+^ pump) (*E*) and contraction (*F*). *G*, ATP_Mg_ dependence of contraction. Lines are simulation results. Symbols are experimental data obtained by Leem et al. (1999) in panel (*A*), Xu et al. (1996) in panel (*B*), Ji et al. (1999) in panel (*C*), Doering and Lederer (1994) in panel (*D*), Friedrich et al. (1996) in panel (*E*), Orchard and Kentish (1990) in panel (*F*) and Mekhfi et al. (1996) in panel (*G*). Simulation data in panels (*F*) and (*G*) were obtained in isometric condition with [Pi_tot_]_i_ = 2.0 mm, [ATP_Mg_]_i_ = 6.5 mm and hsmL = 1.065 µm in panel (*F*) and with [Pi_tot_]_i_ = 0 mm, [PCr]_i_ = 0 or 12 mm, [Ca^2+^]_i_ = 0.001 µM, pHi = 7.1 and hsmL = 1.065 µm in panel (*G*).

Mathematical models of sarcolemmal transporters that regulate pH_i_, namely Cl^−^‐HCO_3_^−^ exchange (anion exchange (AE)), Na^+^‐HCO_3_^−^ cotransporter (NBC), Cl^−^‐OH^−^ exchange (CHE) and NHE, were implemented in a similar manner to previous studies (Crampin & Smith, 2006; Leem et al., 1999). The fluxes of these transporters well fit to experimental data of guinea‐pig ventricular myocyte (Leem et al., 1999) (Fig. 2*A*).

The pH_i_ dependences of ryanodine receptor (RyR), sarcoplasmic/endoplasmic reticulum Ca^2+^ pump (SERCA), sarcolemmal NCX and NaK were modelled in essentially the same manner as Crampin and Smith (2006), with reference to experimental data covering a wider range of pH (Doering & Lederer, 1994; Friedrich et al., 1996; Ji et al., 1999; Xu et al., 1996). Ionic currents of these channels and transporters were multiplied by a pH factor (pHFactor) without assuming specific state dependence. Equations were fit to the data using SigmaPlot software (Grafiti LLC, Palo Alto, CA, USA), as shown in Fig. 2*B–E*.

RyR

$$\mathrm{pHFactor}=\frac{1+10^{1.87\left(-7.15+pK_{\mathrm{RyR}}\right)}}{1+10^{1.87\left(-pH_{i}+pK_{\mathrm{RyR}}\right)}}\;,\;\;pK_{\mathrm{RyR}}=6.64$$

SERCA

$$\mathrm{pHFactor}=\frac{1+10^{1.14\left(-7.14+pK_{\mathrm{SERCA}}\right)}}{1+10^{1.14\left(-pH_{i}+pK_{\mathrm{SERCA}}\right)}}\;,\;\;pK_{\mathrm{SERCA}}=7.53$$

NCX

$$\mathrm{pHFactor}=\frac{1+10^{0.991\left(-7.15+pK_{\mathrm{NCX}}\right)}}{1+10^{0.991\left(-pH_{i}+pK_{\mathrm{NCX}}\right)}}\;,\;\;pK_{\mathrm{NCX}}=7.37$$

NaK

$$\begin{array}{ccc} & & \mathrm{pHFactor}\\ & & \;=\frac{1.0+10^{0.3457\left(-7.15+pK1\right)}}{\left(1.0+10^{0.3457\left(-pH_{i}+pK1\right)}\right)\left(1.0+10^{1.5556\left(-\left(-pH_{i}+pK2\right)\right)}\right)} \end{array}$$

pK1=9.2167,pK2=8.5574

Contraction models by Kuzumoto et al. (2008) and Shim et al. (2007), which were developed based on Negroni and Lascano's model (1996), were modified to reproduce pH_i_‐dependent shifts of pCa dependence and of maximal developed tension (Orchard & Kentish, 1990). Transitions from free troponin C(T) to troponin C bound to Ca^2+^ (TCa), and from free troponin C with crossbridge attached (T^*^) to troponin C bound to Ca^2+^ with crossbridge attached (TCa^*^), were assumed pH_i_ dependent, and transition rates were multiplied by pHFactor1. In addition transition from TCa to TCa^*^ was assumed pH_i_ dependent, and the rate was multiplied by pHFactor2. These assumptions well reproduced the experimental data (Orchard & Kentish, 1990) (Fig. 2*F*).

Contraction

$$\mathrm{pHFactor}1=\frac{1+{\left(\frac{10^{\left(3-7.15\right)}}{10^{\left(3-6.79\right)}}\right)}^{1.65}}{1+{\left(\frac{{\left[H^{+}\right]}_{i}}{10^{\left(3-6.79\right)}}\right)}^{1.65}}$$

$$\mathrm{pHFactor}2=\frac{1+{\left(\frac{10^{\left(3-7.15\right)}}{41.6\times 10^{-6}}\right)}^{1.47}}{1+{\left(\frac{{\left[H^{+}\right]}_{i}}{41.6\times 10^{-6}}\right)}^{1.47}}\;\left(1-0.414\right)+0.414$$

### Modelling mitochondrial function

The model of OXPHOS is essentially the same as our previous models (Kuzumoto et al., 2008; Matsuoka et al., 2004), which consist of Complex I (C1), Complex III (C3), Complex IV (C4), ATP synthase (SN), phosphate translocator (PiC), adenine nucleotide antiport (ANT), proton leak (Hleak) and substrate dehydrogenation (DH). In addition the mitochondrial model includes K^+^ uniporter (KUni), K^+^‐H^+^ exchange (KHE), Ca^2+^ uniporter (CaUni), mitochondrial Na^+^‐Ca^2+^ exchange (NCX_mit_), mitochondrial Na^+^‐H^+^ exchange (NHE_mit_) and mPTP to calculate mitochondrial H^+^, Ca^2+^, Na^+^ and K^+^ concentrations and mitochondrial membrane potential (ΔΨ). Full equations are available in the Supplementary Material.

### ATP and PCr dependence of contraction

Cardiac muscle stiffens when cytosolic ATP bound to Mg^2+^ (ATP_Mg_) is depleted, and the ATP dependence significantly shifts to higher ATP concentration when PCr is absent (Mekhfi et al., 1996), supporting the idea of PCr shuttle system. The apparent dependences of rigour on ATP_Mg_ and PCr were modelled using a function, ATPFactor, which was applied to two transition rates from crossbridge‐attached states to detached states:

$$\mathrm{ATPFactor}=\frac{{\left[{\mathrm{ATP}}_{\mathrm{Mg}}\right]}_{i}^{3}}{{\left[{\mathrm{ATP}}_{\mathrm{Mg}}\right]}_{i}^{3}+K_{m}{\mathrm{ATP}}^{3}}$$

$$K_{m}\mathrm{ATP}=0.464+10\frac{0.1^{2}}{{\left[\mathrm{PCr}\right]}_{i}^{2}+0.1^{2}}$$

As demonstrated in Fig. 2*G* the ATP dependence of model fit well to the data.

### Mitochondrial permeability transition pore

**Figure 3:**
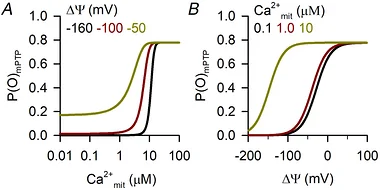
Characteristics of mPTP model *A*, Ca^2+^_mit_ dependence of probability of mPTP in open state (P(O)_mPTP_) at ΔΨ of −160, −100 and −50 mV. *B*, ΔΨ dependence of P(O)_mPTP_ at 0.1, 1.0 and 10 µM Ca^2+^_mit_. Ca^2+^_i_ = 1 µM, pH_mit_ = 7.6, pH_i_ = 7.1. Simulation was performed for 5 min to obtain steady state for each condition and the steady state values are plotted.

A mathematical model of mPTP published previously by Wacquier et al. (2020) was adapted with modification to decrease the permeability of mPTP in the physiological ranges of mitochondrial Ca^2+^ (Ca^2+^_mit_) and ΔΨ (Fig. 3). It is notable that mPTP is almost suppressed in the physiological ranges (normoxic condition) (Boyman et al., 2019; Halestrap, 2009); probability of mPTP in open state (*P*(O)_mPTP_) was 0.001, and the probability increased with an increase in Ca^2+^_mit_ or depolarization of ΔΨ.

### Integration of H^+^ and ATP in mitochondria and cytosol

Cytosolic and mitochondrial H^+^ concentrations ([H^+^]_i_ and [H^+^]_mit_) and cytosolic and mitochondrial total ATP concentrations ([ATP_tot_]_i_ and [ATP_tot_]_mit_) were calculated as follows:

$$\;\frac{d{\left[H^{+}\right]}_{i}}{dt}=\frac{\frac{\left(J_{\mathrm{CHE}}-J_{\mathrm{NHE}}\right)}{V_{i}}+J_{CO_{2}\mathrm{hyd}}-\left(\mathrm{dAT}P_{\mathrm{CK}}+\mathrm{dAT}P_{\mathrm{use}}+\mathrm{HfromMit}+V\mathrm{Heff}\right)}{\mathrm{buffers}}$$

$$\mathrm{HfromMit}=-4v_{C1}-4v_{C3}-2v_{C4}+n_{\mathrm{ATP}}\times v_{\mathrm{SN}}+v_{\mathrm{PiC}}$$

$$+v_{\mathrm{HLeak}}+v_{\mathrm{KHE}}+v_{\mathrm{ANT}}+v_{\mathrm{NHEmit}}-\frac{I_{\mathrm{mPTP}}H}{F\times V_{i}}$$

$$\;\frac{d{\left[H^{+}\right]}_{\mathrm{mit}}}{dt}\;=\;\left(\frac{-5v_{C1}\;-\;2v_{C3}\;-\;4v_{C4}\;+\;v_{\mathrm{DH}}\;+\;\left(n_{\mathrm{ATP}}\;-\;1\right)\;v_{\mathrm{SN}}\;+\;v_{\mathrm{PiC}}\;+\;v_{\mathrm{Hleak}}\;+\;v_{\mathrm{KHE}}\;+\;v_{\mathrm{ANT}}\;+\;v_{\mathrm{NHEmit}}}{R_{\mathrm{mc}}}\;-\;\frac{I_{\mathrm{mPTP}}H}{F\times V_{\mathrm{mit}}}\right)\;\times \;\frac{1}{R\mathrm{bu}f_{\mathrm{mit}}}$$

$$\;\frac{d{\left[{\mathrm{ATP}}_{\mathrm{tot}}\right]}_{i}}{dt}=v_{\mathrm{ANT}}+\mathrm{dAT}P_{\mathrm{CK}}+{\mathrm{dATP}}_{\mathrm{AK}}+{\mathrm{dATP}}_{\mathrm{Gly}}+{\mathrm{dATP}}_{\mathrm{use}}$$

$$\;\frac{d{\left[\mathrm{AT}P_{\mathrm{tot}}\right]}_{\mathrm{mit}}}{dt}=\frac{v_{\mathrm{SN}}-v_{\mathrm{ANT}}}{R_{\mathrm{mc}}}$$

*J*_CHE_ is the ion flux of CHE, *J*_NHE_ is the ion flux of NHE, J_CO2hyd_ represents the CO_2 _hydration reaction, dATP_CK_ is the rate of ATP use by creatine kinase and dATP_use_ is the rate of ATP use by NaK, SERCA, PMCA (plasma membrane Ca^2+^‐ATPase) and contraction. HfromMit represents H^+^ flux from mitochondria, *v*_X_ is the rate of X (C1, C3, C4, DH, SN, PiC, Hleak, KHE, ANT, NHE_mit_, KUni), *I*_mPTP_H represents the H^+^ current through mPTP, *V*Heff is the rate of H^+^ efflux from cytosol, buffers represents H^+^ buffer in cytosol, *F* is Faraday's constant (96.4867 C mmol^−1^), *V*_i_ is the cytosolic volume (12,800 µm^3^), *n*_ATP_ = 2.5, *R*_mc_ (0.2875) is the ratio of mitochondrial volume (*V*_mit_ = 3,680 µm^3^) to cytosolic volume, *R*buf_mit_ represents H^+^ buffering capacity coefficient in mitochondria, dATP_AK_ is the rate of ATP production by adenylate kinase and dATP_Gly_ is the rate of ATP production by glycolysis.

**Figure 4:**
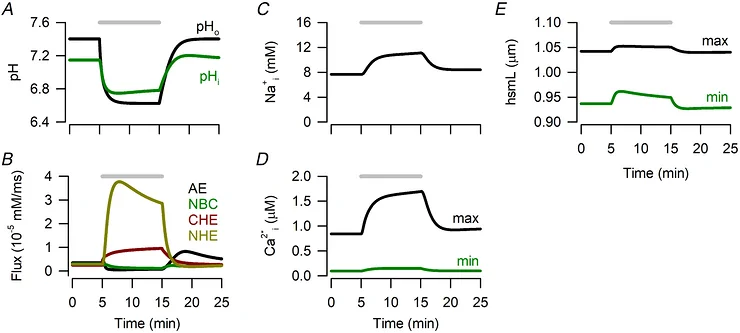
Simulation of respiratory acidosis on cardiomyocyte Extracellular CO_2_ was changed from 5% to 30% between 5 and 15 min (light‐grey bar period). *A*, pH_o_ and pH_i_; *B*, H^+^ fluxes via AE (anion exchange), CHE (Cl^−^‐OH^−^ exchange), NBC (Na^+^‐HCO_3_^−^ cotransporter) and NHE. *C*, concentration of Na^+^_i_; *D*, concentration of Ca^2+^_i_; and *E*, force. Stimulation interval was 400 ms.

**Figure 5:**
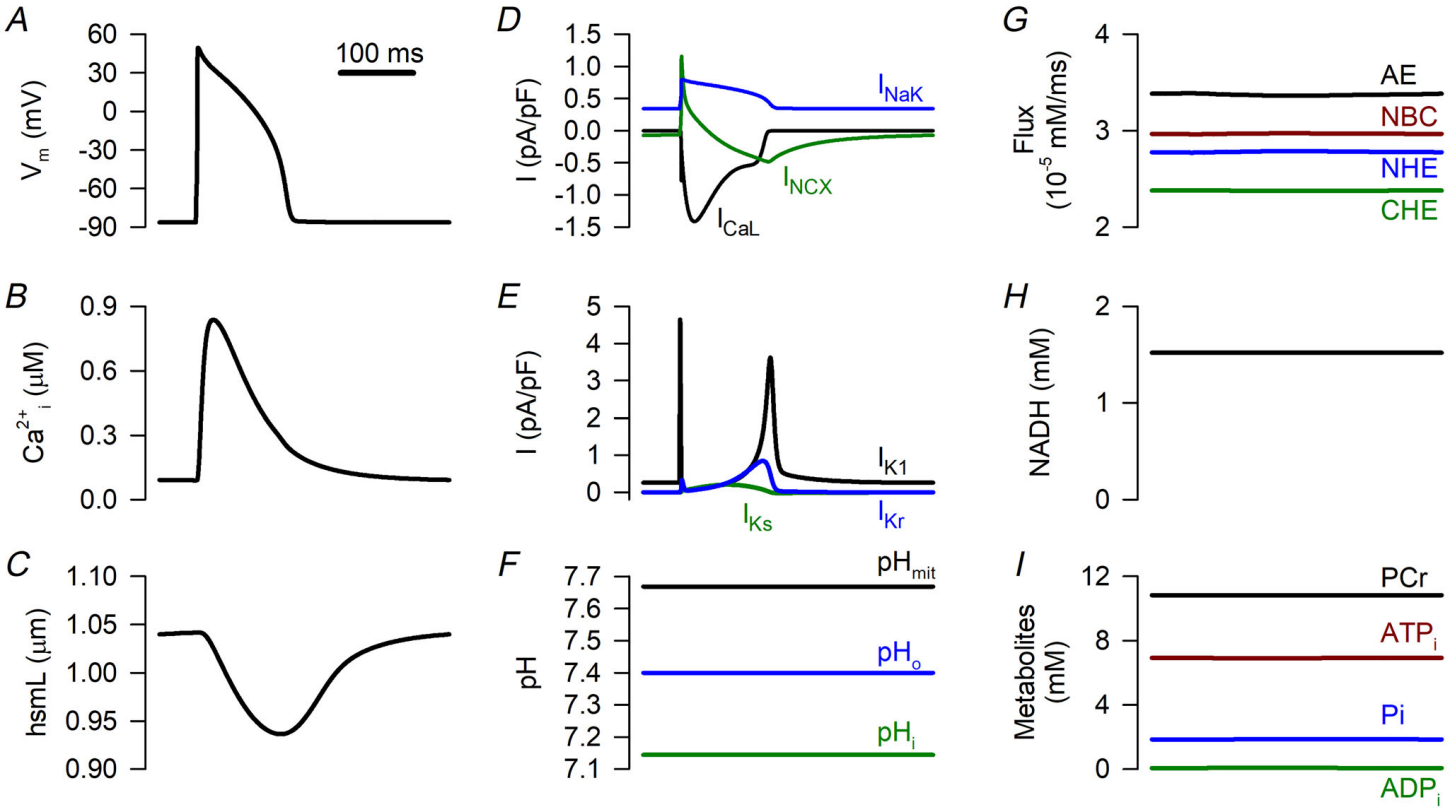
Time courses of major variables of the integrated model during one cycle in normoxic conditions *A*, *V*_m_. *B*, concentration of Ca^2+^_i_. *C*, hsmL (half sarcomere length). *D*, L‐type Ca^2+^ current (*I*_CaL_), Na^+^/Ca^2+^ exchange current (*I*_NCX_) and NaK (Na^+^/K^+^ pump) current (*I*_NaK_). *E*, delayed rectifier K^+^ current, slow component (*I*_Ks_), inward rectifier K^+^ current (*I*_K1_) and delayed rectifier K^+^ current, rapid component (*I*_Kr_). *F*, pH_mit_, pH_o_ and pH_i_. *G*, ion flux through AE (anion exchange), NBC (Na^+^‐HCO_3_^−^ cotransporter), CHE (Cl^−^‐OH^−^ exchange) and NHE. *H*, concentration of NADH in mitochondria. *I*, concentrations of PCr (phosphocreatine), ATP_i_, Pi_tot_ (P_i_ (inorganic phosphate)) and ADP_i_.

The integrated model successfully reproduced cardiac responses to respiratory acidosis (Fig. 4) as well as those at normoxic conditions as demonstrated later in Fig. 5. Respiratory acidosis increased Ca^2+^_i_ transient and attenuated contraction as demonstrated in isolated papillary muscle (Orchard & Kentish, 1990). In the integrated model an increase in extracellular CO_2_ (CO_2o_) from 5% to 30% induced extracellular and intracellular acidosis (Fig. 4*A*) and elevated the concentration of Na^+^_i_ (Fig. 4*C*) due to augmented Na^+^ influx through NHE (Fig. 4*B*). The increase in Na^+^_i_ and acidosis attenuated Ca^2+^ efflux via NCX, resulting in an increase in Ca^2+^_i_ transient (Fig. 4*D*). Inhibition of contraction by acidosis overcame the contraction‐enhancing effect of increased Ca^2+^_i_ transient, resulting in the attenuation of contraction during acidosis (Fig. 4*E*). These results were essentially similar to the experimental data (Orchard & Kentish, 1990) though the attenuation of contraction was relatively weaker in this simulation.

### Protocol for non‐flow global ischaemia and reperfusion

The model cell was electrically stimulated every 400 ms. Non‐flow global ischaemia was simulated by a decline in extracellular O_2_ (O_2o_) concentration and the accumulation or depletion of ions and CO_2_ in a restricted extracellular space. The O_2o_ decline was expressed by two exponentials, and O_2o_ recovery during reperfusion was expressed by a single exponential. The time constants were chosen so that time courses of changes in PCr and NADH fall within the experimental data range, and the standard duration of ischaemia was defined as 15 min, because changes in many variables are in line with experimental data, as shown in Fig. 7.

Scully et al. (2018) reported that the extracellular volume of heart was 20%–26% of heart tissue. The restricted extracellular volume (*V*_o_) was set in this study as 34% of cell volume (=∼25% of total space) to simulate accumulation of ions and CO_2_ during ischaemia, and released during normoxic and reperfusion periods.

Kuzmiak‐Glancy et al. (2022) reported that the activity of every component of the OXPHOS – that is, the dehydrogenase enzymes, the electron transport chain and ATP synthesis – decreased to ∼50% after the 60‐min global non‐flow ischaemia in isolated mitochondria. According to this report the activities of all the mitochondrial components except for H^+^ and Ca^2+^ buffers in the integrated model decreased to 50% of the control values during reperfusion after the standard 15‐min ischaemia. The effects of the mitochondrial impairments on cardiomyocyte function were systematically analysed in this study.

Normoxia

$$\;{\left[O_{2}\right]}_{o}=0.24\;\mathrm{mM}$$

$$\;\frac{d{\left[Na^{+}\right]}_{o}}{dt}=0.0,\;\;\frac{d{\left[K^{+}\right]}_{o}}{dt}=0.0,\;\;\frac{d{\left[Ca^{2+}\right]}_{o}}{dt}=0.0,$$

$$\;\frac{d{\left[Cl^{-}\right]}_{o}}{dt}=0.0,\;\;\frac{d{\left[CO_{2}\right]}_{o}}{dt}=0.0$$

Ischaemia

$$\;{\left[O_{2}\right]}_{o}=0.24\left(0.99e^{\left(\frac{t_{i}-t}{\tau_{i1}}\right)}+0.01e^{\left(\frac{t_{i}-t}{\tau_{i2}}\right)}\right)$$

$$\;\tau_{i1}=8,000\;\left(\mathrm{ms}\right),\;\;\tau_{i2}=8,000,000\;\left(\mathrm{ms}\right),$$

$$t_{i}\;\mathrm{is}\;\mathrm{the}\;\mathrm{time}\;\mathrm{when}\;\mathrm{ischaemia}\;\mathrm{begins}:\;\;180,000\;\mathrm{ms}$$

$$\begin{array}{ccc} & & \frac{d{\left[Na^{+}\right]}_{o}}{dt}=\frac{\frac{I\mathrm{cNa}}{F}-\left(J_{\mathrm{NKCC}1}+J_{\mathrm{NHE}}+J_{\mathrm{NBC}}\right)\;}{V_{o}}\;,\\ & & \frac{d{\left[K^{+}\right]}_{o}}{dt}=\frac{\frac{I\mathrm{cK}}{F}-J_{\mathrm{NKCC}1}\;}{V_{o}} \end{array}$$

$$\begin{array}{ccc} & & \frac{d{\left[Ca^{2+}\right]}_{o}}{dt}=\frac{\frac{I\mathrm{cCa}}{2F}\;}{V_{o}}\;,\\ & & \frac{d{\left[Cl^{-}\right]}_{o}}{dt}=\frac{\frac{-I\mathrm{cCl}}{F}-\left(2J_{\mathrm{NKCC}1}+J_{\mathrm{CHE}}+J_{\mathrm{AE}}\right)}{V_{o}}\;, \end{array}$$

$$\;\frac{d{\left[{\mathrm{CO}}_{2}\right]}_{o}}{dt}=\frac{J_{{\mathrm{CO}}_{2}}\times V_{i}}{V_{o}}$$

*I*cNa is the total Na^+^ current, *J*_NKCC1_ represents the ion flux of Na^+^/K^+^/2Cl^−^ cotransporter 1 (NKCC1); *J*_NHE_ represents the ion flux of NHE; *J*_NBC_ represents the ion flux of NBC, *I*cK is the total K^+^ current, *I*cCl is the total Cl^−^ current, *J*_CHE_ represents the ion flux of CHE, *J*_AE_ represents the ion flux of AE, *F* is Faraday's constant and $J_{CO_{2}}$ represents the diffusive flux of CO_2_ across sarcolemmal membrane.

Reperfusion

$$\;{\left[O_{2}\right]}_{o}={\left[O_{2}\right]}_{o\_i}+\left(0.24-{\left[O_{2}\right]}_{o\_i}\right)\left(1.0-e^{\left(\frac{t_{r}-t}{\tau_{r}}\right)}\right)$$

$$\begin{array}{ccc} & & \tau_{r}=60,000\;\mathrm{ms};\;t_{r}\mathrm{is}\;\mathrm{the}\;\mathrm{time}\;\mathrm{when}\;\mathrm{reperfusion}\;\mathrm{begins}:\\ & & 1,080,000\;\mathrm{ms} \end{array}$$

$${\left[O_{2}\right]}_{o\_i}:{\left[O_{2}\right]}_{o}\;\mathrm{at}\;\mathrm{the}\;\mathrm{end}\;\mathrm{of}\;\mathrm{ischaemia}$$

$$\;\frac{d{\left[Na^{+}\right]}_{o}}{dt}=\frac{\left(140.0-{\left[Na^{+}\right]}_{o}\right)}{\tau_{r}}\;,\;\;\frac{d{\left[K^{+}\right]}_{o}}{dt}=\frac{\left(5.4-{\left[K^{+}\right]}_{o}\right)}{\tau_{r}}$$

$$\;\frac{d{\left[Ca^{2+}\right]}_{o}}{dt}=\frac{\left(1.8-{\left[Ca^{2+}\right]}_{o}\right)}{\tau_{r}}\;,\;\;\frac{d{\left[Cl^{-}\right]}_{o}}{dt}=\frac{\left(140.0-{\left[Cl^{-}\right]}_{o}\right)}{\tau_{r}}$$

$$\;\frac{d{\left[CO_{2}\right]}_{o}}{dt}=\frac{\left(1.236-{\left[CO_{2}\right]}_{o}\right)}{\tau_{r}}$$

### Sensitivity analysis

Sensitivity was defined as the relative changes in Na^+^_i_ and Ca^2+^_i_ concentrations immediately prior to stimulus pulse. These values were calculated using linear regression by varying the amplitude factor (AF) for each component by ±1%, ±2% and 0%. The standard simulation protocol was performed after 1‐h stabilization under normoxic conditions. Data were collected immediately before ischaemia (normoxia, 3 min after start of simulation), after 15 min of ischaemia (18 min) and after 32 min of reperfusion (50 min) in the ischaemia–reperfusion protocol.

## Results

### Excitation‐contraction–energetics coupling in normoxic conditions

Figure 5 demonstrates representative variables of excitation‐contraction‐energetics coupling in normoxic conditions. The configurations of action potential, Ca^2+^_i_ transient, half sarcomere length (hsmL) and ion currents through major channels/transporters were essentially similar to those reported in Kuzumoto et al. (2008). Concentrations of diastolic and systolic Ca^2+^_i_ (0.09 and 0.84 µm) and Na^+^_i_ (7.6 mm) were within a physiological range. The bicarbonate buffer and the four sarcolemmal pH_i_‐regulating transporters maintained cytosolic and mitochondrial pH within a physiological range (pH_i_ = 7.14 and pH_mit_ = 7.67), and no major transient changes were observed in the four pH_i_‐regulating transporters during one cycle. ΔΨ (−155.1 mV) and energy metabolites (e.g. PCr = 10.8 mm; total cytosolic ATP (ATP_i_) = 6.9 mm; Pi = 1.9 mm; total cytosolic ADP (ADP_i_) = 0.08 mm) were also within a physiological range and were stable during one cycle.

### Excitation‐contraction–energetics coupling during ischaemia and reperfusion

**Figure 6:**
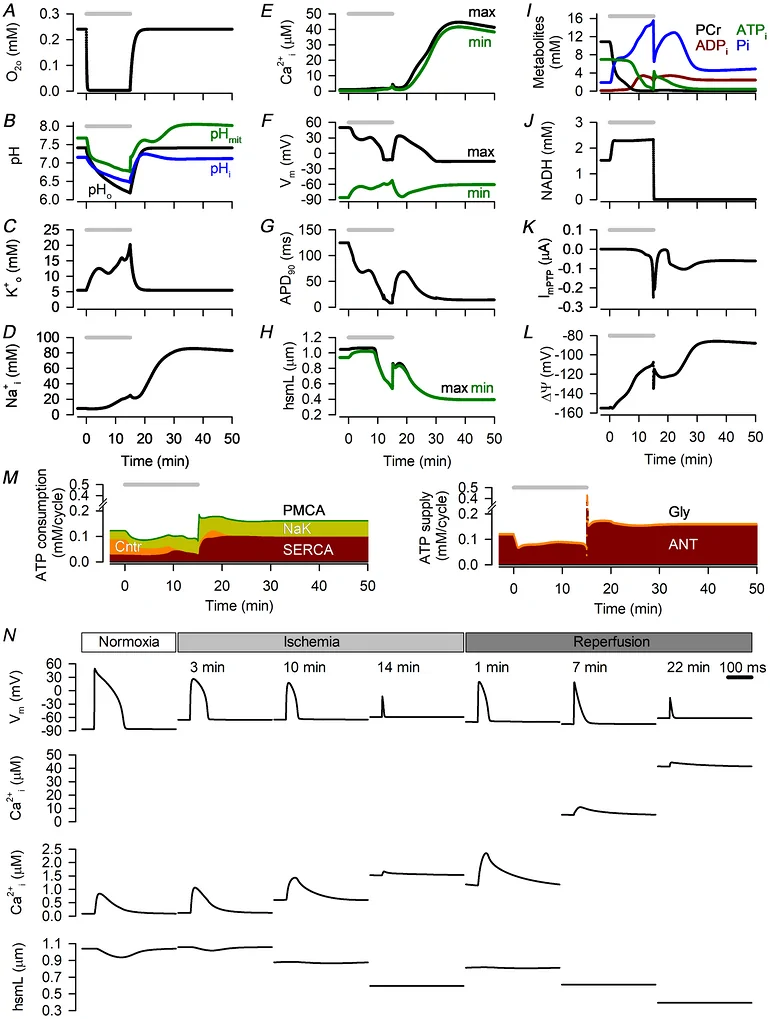
Simulation of ischaemia and reperfusion with the integrated model *A*, extracellular oxygen (O_2o_). O_2o_ concentration was set as described in ‘Methods’ section. Major variables in excitation–contraction–energetics coupling are demonstrated as follows. *B*, pH in extracellular solution (pH_o_), in mitochondria (pH_mit_) and in cytosol (pH_i_). *C*, extracellular K^+^ (K^+^_o_) concentration. *D*, cytosolic Na^+^ (Na_i_) concentration. *E*, cytosolic free Ca^2+^ (Ca^2+^_i_) concentration. *F*, maximum and minimum values of membrane potential (*V*_m_) during one cycle. *G*, action potential duration at 90% repolarization (APD_90_). *H*, maximum and minimum values of half sarcomere length (hsmL). *I*, cytosolic concentration of PCr (phosphocreatine), ATP_i_, ADP_i_ and Pi (inorganic phosphate). *J*, mitochondrial NADH concentration. *K*, mPTP (mitochondrial permeability transition pore) current (*I*_mPTP_). *L*, mitochondrial membrane potential (ΔΨ). Values at the end of one cycle were plotted. *M*, left panel: ATP consumptions per cycle by PMCA (plasma membrane Ca^2+^‐ATPase), NaK (Na^+^/K^+^ pump), Cntr (contraction) and SERCA (sarcoplasmic/endoplasmic reticulum Ca^2+^ pump). Right panel: ATP supplies to cytosol per cycle by Gly and ANT (adenine nucleotide antiport). *N*, configurations of action potential, Ca^2+^_i_ transient and cell shortening as revealed by hsmL during one stimulus cycle. Note that the scale for Ca^2+^_i_ is 0–2.5 µm in the first 5 transients, followed by the scale of 0–50 µm in the last 2 transients. Ischaemia period is indicated by a grey bar.

**Figure 7:**
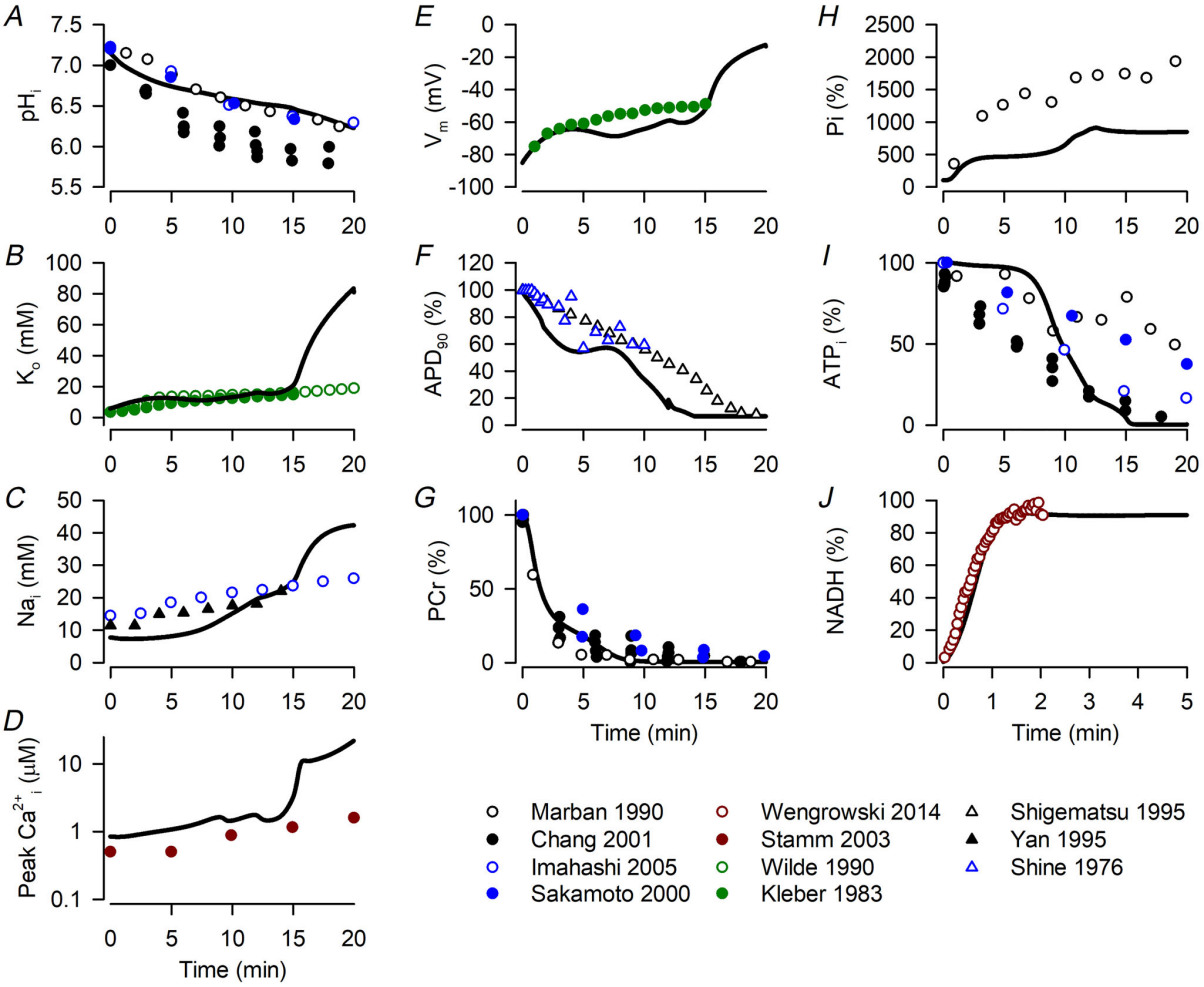
Comparison of simulation results with experimental data Simulation results during 20 min of ischaemia were compared with experimental data. *A*, pH_i_; *B*, *K*_o_ concentration; *C*, Na^+^_i_ concentration; *D*, maximal Ca^2+^_i_ concentration per cycle; *E*, resting *V*_m_; *F*, APD_90_; *G*, PCr (phosphocreatine) concentration normalized to control value; *H*, Pi (inorganic phosphate) concentration normalized to control value; *I*, ATP_i_ concentration normalized to control value; *J*, NADH concentration normalized to maximal value. Simulation results are shown as lines and experimental data as symbols (Chang et al., 2001; Imahashi et al., 2005; Kleber, 1983; Marban et al., 1990; Sakamoto et al., 2000; Shigematsu et al., 1995; Shine et al., 1976; Stamm et al., 2003; Wengrowski et al., 2014; Wilde et al., 1990; Yan et al., 1995).

The integrated model reconstructed many phenomena observed experimentally during cardiac ischaemia and reperfusion (Fig. 6). Comparisons to experimental data of 20 min of global ischaemia are shown in Fig. 7. O_2o_ was set to decrease exponentially from 0.24 to ∼2.14×10^−3 ^mm during 15 min of ischaemia (Fig. 6*A*). During ischaemia pH_o_, pH_i_ and pH_mit_ decreased to 6.18, 6.47 and 6.76, respectively (Fig. 6*B*). The decrease in pH_i_ was consistent with the experimental data of Marban et al. (1990) and Sakamoto et al. (2000), but was slower compared to the data of Chang et al. (2001) (Fig. 7*A*). K^+^_o_ increased to 20.2 mm for the first 15 min of ischaemia (Fig. 6*C*), which was close to ∼15 mm reported in guinea‐pig Langendorff perfused heart (Kleber, 1983; Wilde et al., 1990), and thereafter soared at the end of 20 min of ischaemia (Fig. 7*B*). Na^+^_i_ increased to 24.6 mm for 15 min of ischaemia (Fig. 6*D*), which was also close to the reported data(Imahashi et al., 2005;  Yan et al., 1995) (Fig. 7*C*). Cytosolic Ca^2+^_i_ (Fig. 6*E*) slightly increased, and resting *V*_m_ (Fig. 6*F*) depolarized in a similar manner to experimental data (Fig. 7*D* and *E*) up to 15 min of ischaemia (Kleber, 1983; Stamm et al., 2003). Action potential (Fig. 6*F* and *G*) suppressed during ischaemia with faster time course compared to experimental data of isolated guinea‐pig right ventricular walls (Shigematsu et al., 1995) (Fig. 7*F*). Notably contraction (Fig. 6*H* and *N*) ceased ∼5 min before the cessation of action potential, which is consistent with experimental data (Shigematsu et al., 1995; Shine et al., 1976).

Experiments using nuclear magnetic resonance spectroscopy revealed drastic change in energy metabolites during cardiac ischaemia, namely a depletion of PCr, an increase in Pi and later a decrease in ATP_i_ (Chang et al., 2001; Marban et al., 1990; Sakamoto et al., 2000). These alterations are reproduced well as shown in Fig. 6*I* and Fig. 7*G–I*, though ATP_i_ decreased more sharply and % change in Pi was smaller than experimental data.

**Figure 8:**
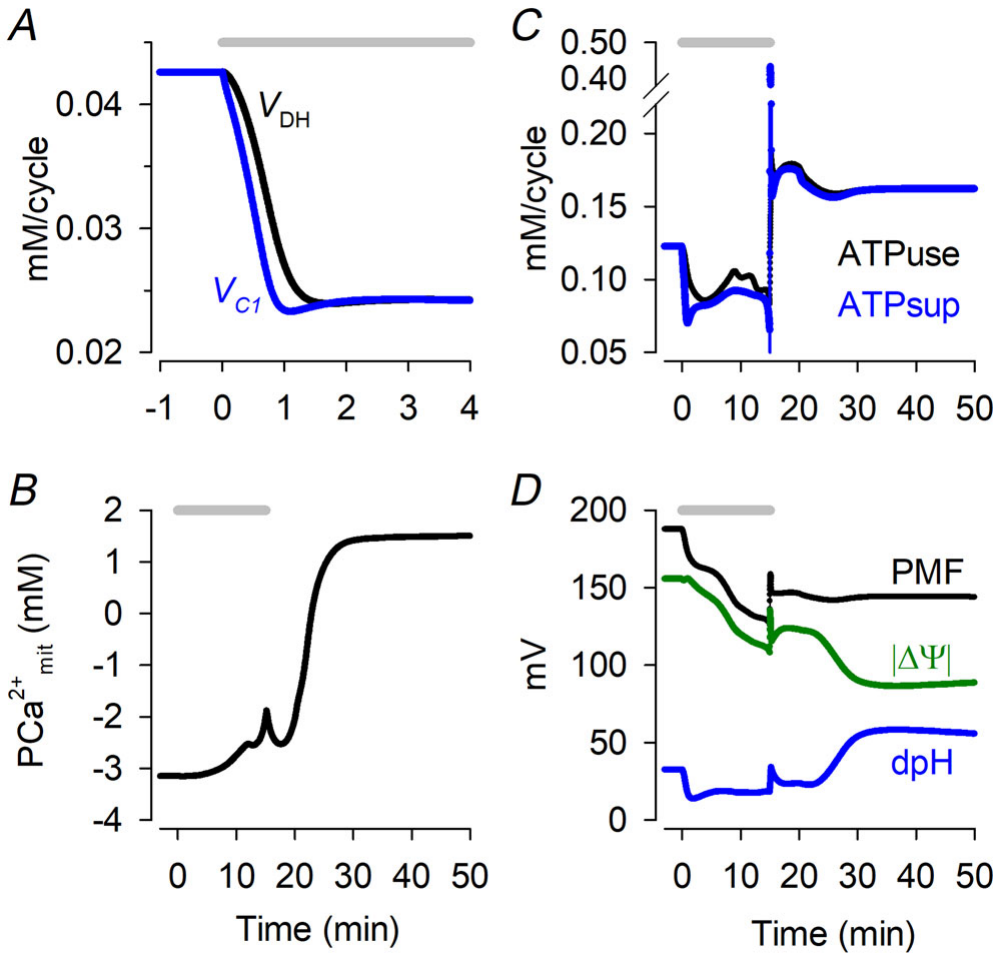
Mitochondrial variables in the ischaemia–reperfusion protocol *A*, rates of substrate dehydrogenation (*v*_DH_) and Complex I (*v*_C1_). *B*, mitochondrial Ca^2+^ (Ca^2+^_mit_). *C*, sum of ATP consumption (ATPuse) by NaK (Na^+^/K^+^ pump), SERCA (sarcoplasmic/endoplasmic reticulum Ca^2+^ pump), contraction and PMCA (plasma membrane Ca^2+^‐ATPase) per cycle and ATP supply (ATPsup) from mitochondria via ANT (adenine nucleotides antiport). *D*, proton motive force (PMF, black), |ΔΨ| (dark green) and dpH (blue). PMF = dPH + |ΔΨ|. Grey bars indicate the ischaemia period.

Oxygen deficiency suppressed mitochondrial Complex IV, leading to suppression of NADH utilization by Complex I and a monotonical increase in NADH (Figs 6*J* and 7*J*) with a similar time course to experimental data (Wengrowski et al., 2014). Although both the rate of substrate dehydrogenation (*v*_DH_), namely the rate of NADH production, and the rate of Complex I (*v*_C1_), namely the rate of NADH consumption, decreased similarly, their imbalance (Fig. 8*A*) caused NADH to increase. Ca^2+^_mit_ increased gradually during ischaemia, but it increased explosively at the late stage (Fig. 8*B*) due to the opening of mPTP (Fig. 6*K*). The opening of mPTP also induced ΔΨ depolarization (Fig. 6*L*), as demonstrated in rabbit ventricular myocytes (Seidlmayer et al., 2015) and mouse neonatal cardiomyocytes (Ashok et al., 2023).

Under normoxic conditions ATP consumption and ATP supply from mitochondria were balanced, but both decreased under ischaemic conditions, with the decrease in ATP supply from mitochondria being larger (Fig. 8*C*). The decrease in mitochondrial ATP supply was due to the decrease in proton motive force caused mainly by a decrease in |ΔΨ|, whereas the pH difference between mitochondria and cytosol (dpH) was relatively preserved after an early drop (Fig. 8*D*) because of a nearly parallel decrease in pH_i_ and pH_mit_ (Fig. 6*B*). As shown in Fig. 6*M* the main ATP consumer under normoxic conditions was contraction (∼50%). During ischaemia ATP consumption by contraction decreased, and NaK became the main ATP consumer. During the last several minutes of ischaemia, ATP consumption by NaK and SERCA further increased.

Simulated reperfusion, namely reoxygenation and release of extracellular diffusion restriction, paradoxically caused further damage to the model cell, though pH_i_ returned to the normal level (Fig. 6*B*). Na^+^_i_ and Ca^2+^_i_ (Fig. 6*D* and *E*) further increased after a transient decrease. ATP_i_ recovered upon reperfusion but then decreased again to ∼0.4 mm (Fig. 6*I*). Action potential partially recovered, and contracture was significantly reduced, but subsequently, along with the decrease in ATP_i_, action potential was suppressed and contracture progressed again (Fig. 6*F*–*H*). The total ATP consumption increased during reperfusion by ∼30% compared to normoxic conditions, and SERCA became the main ATP consumer (∼60%). These phenomena were essentially consistent with experimentally reported reperfusion injury (Ashok et al., 2023; Carmeliet, 1999; Seidlmayer et al., 2015; Shigematsu et al., 1995), though exacerbation of Na^+^_i_ and Ca^2+^_i_ overloads was more severe than the reported data. The extreme increases in Na^+^_i_ and Ca^2+^_i_ during reperfusion may be caused by severe ATP depletion during ischaemia. Changes in representative variables (*V*_m_, Ca^2+^_i_ and hsmL) during one cycle are plotted in Fig. 6*N*. The model cell ceased to produce an effective action potential after 15 min of reperfusion and remained in a contracture state throughout the reperfusion period.

### Na^+^ overload during ischaemia and reperfusion

**Figure 9:**
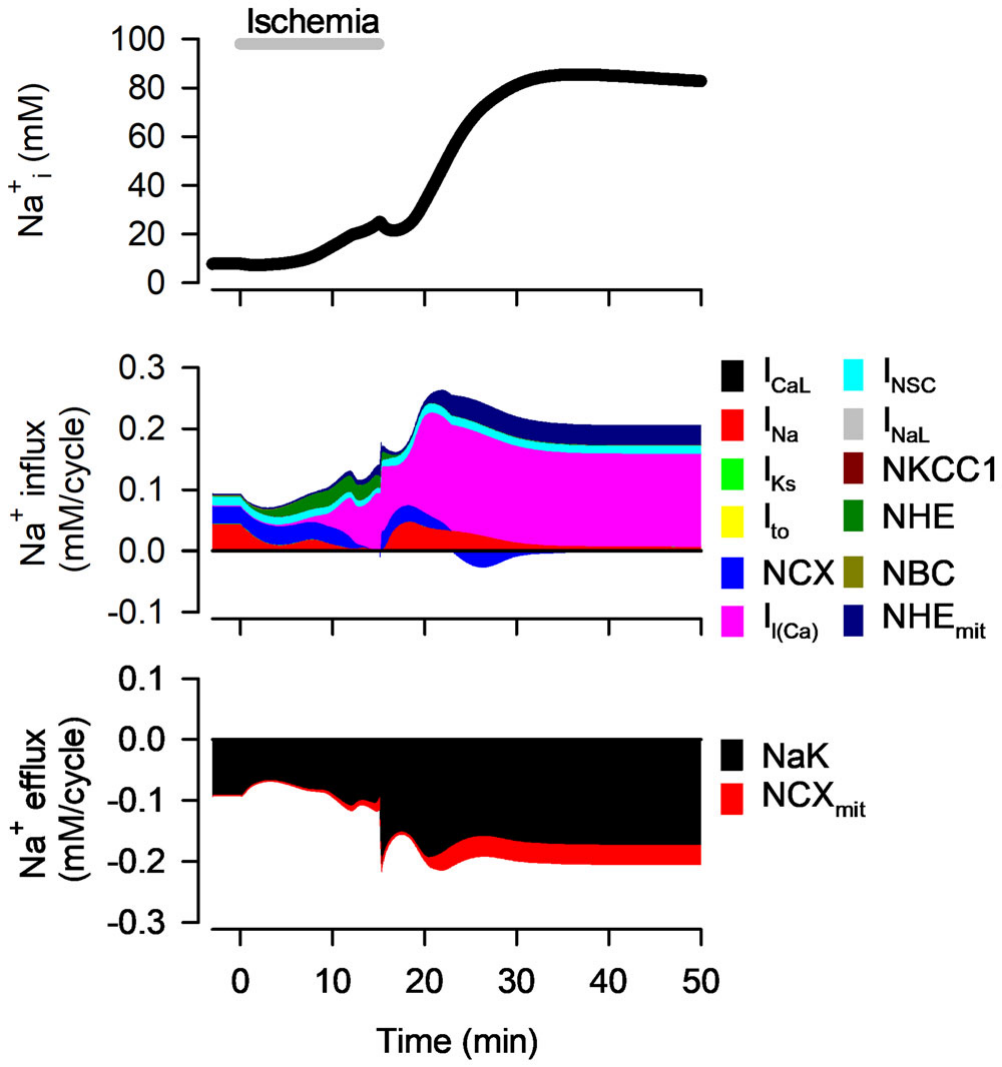
Na^+^ influx to and efflux from cytosol Upper panel: concentration of Na^+^_i_. Same as Fig. 6*D*. Middle panel: Na^+^ influx to cytosol per cycle. Bottom panel: Na^+^ efflux from cytosol per cycle. Each flux was calculated as the sum of the fluxes for one cycle.

As shown in Fig. 6*D* one of the drastic changes during ischaemia and reperfusion was accumulation of Na^+^_i_. Na^+^ influx to and efflux from cytosol are plotted in Fig. 9. Under normoxic conditions major Na^+^ influx was mediated by Na^+^ current (*I*_Na_) and NCX, and major Na^+^ efflux was through NaK. During ischaemia acidosis directly activated Na^+^ influx through NHE and attenuated Na^+^ efflux through NaK (Fig. 2*A* and *E*). In the first several minutes of ischaemia however, Na^+^ influx through *I*_Na_ was indirectly attenuated due to depolarization of resting *V*_m_ (Fig. 6*F*), which was caused by K^+^_o_ increase and K^+^_i_ decrease associated with NaK attenuation by acidosis. The decrease in Na^+^ influx and efflux kept Na^+^_i_ almost constant during the first several minutes. Thereafter further acidosis augmented Na^+^ influxes through NHE, and the subsequent increase in Ca^2+^_i_ induced the Ca^2+^‐activated background cation current (*I*_l(Ca)_) which became the major source of Na^+^ influx, resulting in a gradual increase in Na^+^_i_ to ∼25 mm.

Reperfusion restored pH_i_ in the normal range and partially but temporally restored resting *V*_m_. The partial recovery of resting *V*_m_ however restored Na^+^ influx via *I*_Na_ and augmented Na^+^ influx via *I*_l(Ca)_, resulting in a further elevation of Na^+^_i_. Na^+^ efflux through NaK increased after the onset of reperfusion due to the partial recovery of ATP_i_, but NaK activity could not fully function because of extremely low ATP_i_ (∼0.4 mm) at a later phase of reperfusion (Fig. 6*I*).

**Figure 10:**
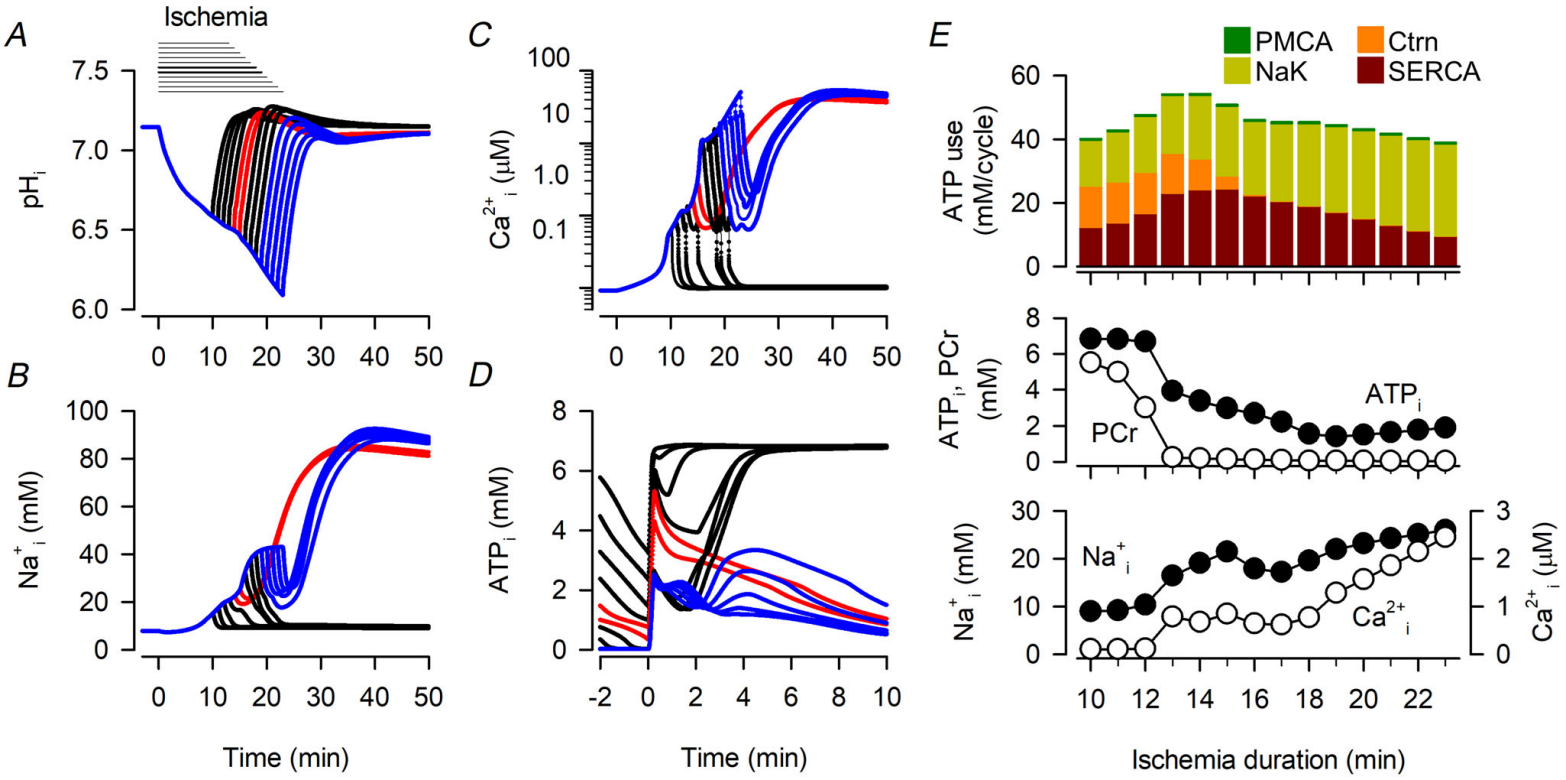
Dependence of reperfusion‐induced disturbances on ischaemia duration *A*, pH_i_. *B*, Na^+^_i_ concentration. *C*, Ca^2+^_i_ concentration. *D*, ATP_i_ concentration. Black lines represent ischaemia durations of 10–13 and 16–18 min, red lines represent ischaemia durations of 14 and 15 min and blue lines represent ischaemia durations of 19–23 min. In panel (*D*) traces were plotted with the reperfusion start time set to 0. *E*, upper panel: sum of ATP consumption by PMCA (plasma membrane Ca^2+^‐ATPase), NaK (Na^+^/K^+^ pump), contraction (Ctrn) and SERCA (sarcoplasmic/endoplasmic reticulum Ca^2+^ pump) per cycle for 2 min after start of reperfusion. Middle panel: PCr (phosphocreatine) and ATP_i_ concentrations at 2 min after start of reperfusion. Lower panel: Na^+^_i_ and Ca^2+^_i_ concentrations at 2 min after start of reperfusion. Values are plotted for each ischaemia duration.

The reperfusion injury, characterized by abnormal action potential, Na^+^_i_ and Ca^2+^_i_ overloads and contracture, depended on the duration of ischaemia (Fig. 10*A–D*). The model cell fully recovered from ischaemia‐induced abnormalities when the duration of ischaemia was <13 min using this ischaemia–reperfusion protocol. Unexpectedly a longer duration of 16–18 min did not induce reperfusion injury, whereas even longer durations did induce the injury. As shown in Fig. 10*D* a critical point determining whether ATP_i_ level recovered or decreased occurs within a few minutes of reperfusion. With ischaemic durations of 16–18 min, ATP consumption during the first 2 min of reperfusion was smaller than that during shorter periods of ischaemia (Fig. 10*E*, upper panel). This was because depletion of PCr shifted ATP dependence of contraction towards higher concentrations, thereby preventing ATP consumption by contraction. The cessation of contraction enabled the preferential allocation of ATP to NaK and SERCA, leading to the relief of Na^+^_i_ and Ca^2+^_i_ overloads (Fig. 10*E*).

### Possible determinants of ischaemia–reperfusion injury

**Figure 11:**
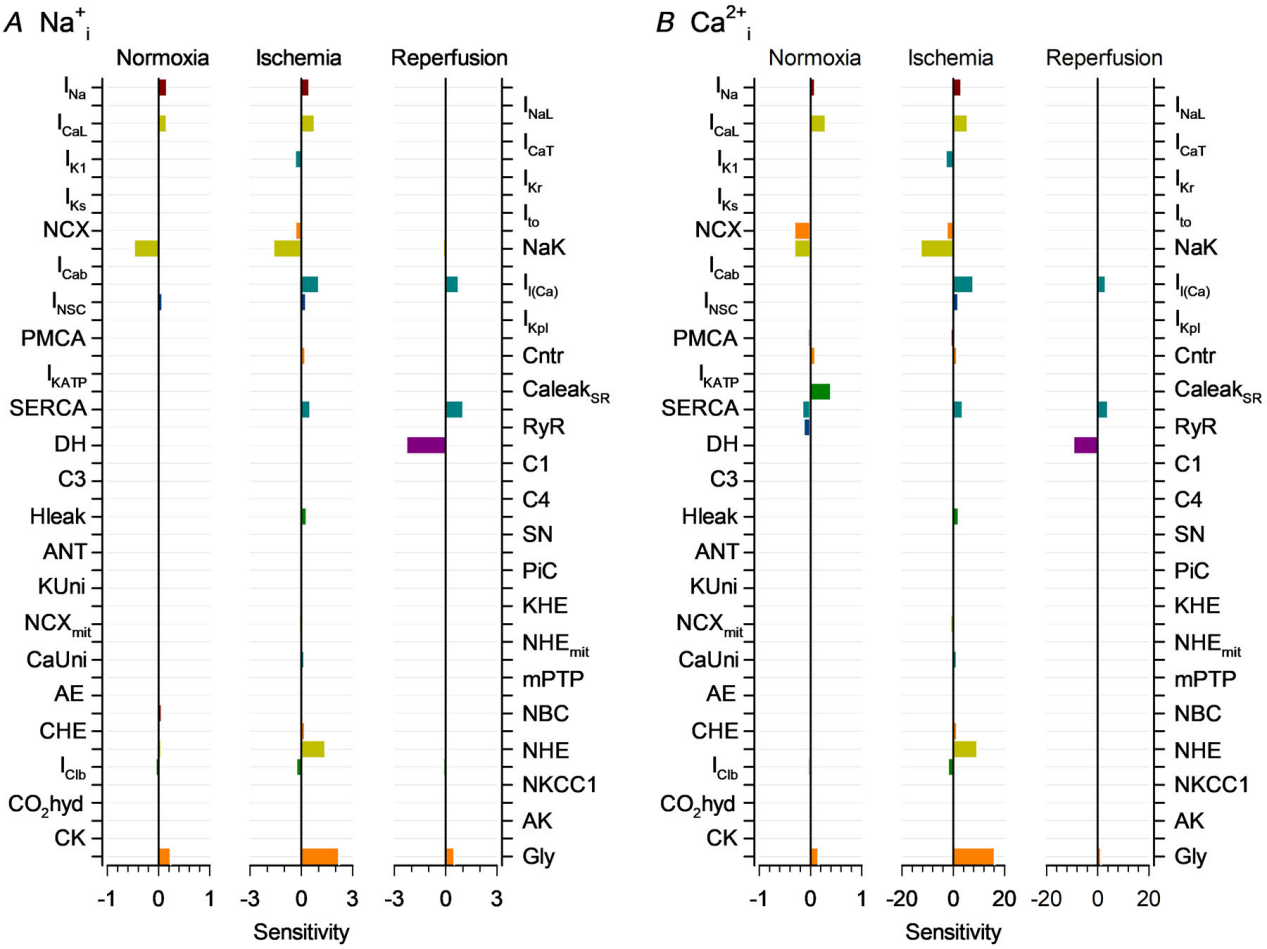
Sensitivity analysis on Na^+^_i_ and Ca^2+^_i_ Sensitivities of major components for Na^+^_i_ (*A*) and Ca^2+^_i_ (*B*) in normoxia, 15 min of ischaemia and 32 min of reperfusion were plotted.

To explore the possible determinants of ischaemia–reperfusion injury, we performed sensitivity analyses. The sensitivity of each component was obtained under normoxic conditions, after 15 min of ischaemia and after 32 min of reperfusion in the ischaemia–reperfusion protocol. The sensitivity of each component was determined for two major variables: Na^+^_i_ and Ca^2+^_i_ (Fig. 11*A* and *B*). Generally the sensitivity values were relatively small in the normoxic period, indicating the robustness of the system, whereas the sensitivity values increased at the end of ischaemia and different factors contributed to the response. With regard to Na^+^_i_ (Fig. 11*A*), NaK had a great reducing influence on Na^+^_i_ in normoxic and ischaemic conditions, with almost no influence at the end of reperfusion. To our surprise mitochondrial substrate dehydrogenation (DH), namely NADH production, had a strong reducing impact on Na^+^_i_ (Fig. 11*A*) in reperfusion. DH also had normalizing effects on Ca^2+^_i_ (Fig. 11*B*) in reperfusion, suggesting that DH influences reperfusion injury. In the following section we further analyse the role of DH in the injury.

### NADH production rate is a determinant of reperfusion injury

**Figure 12:**
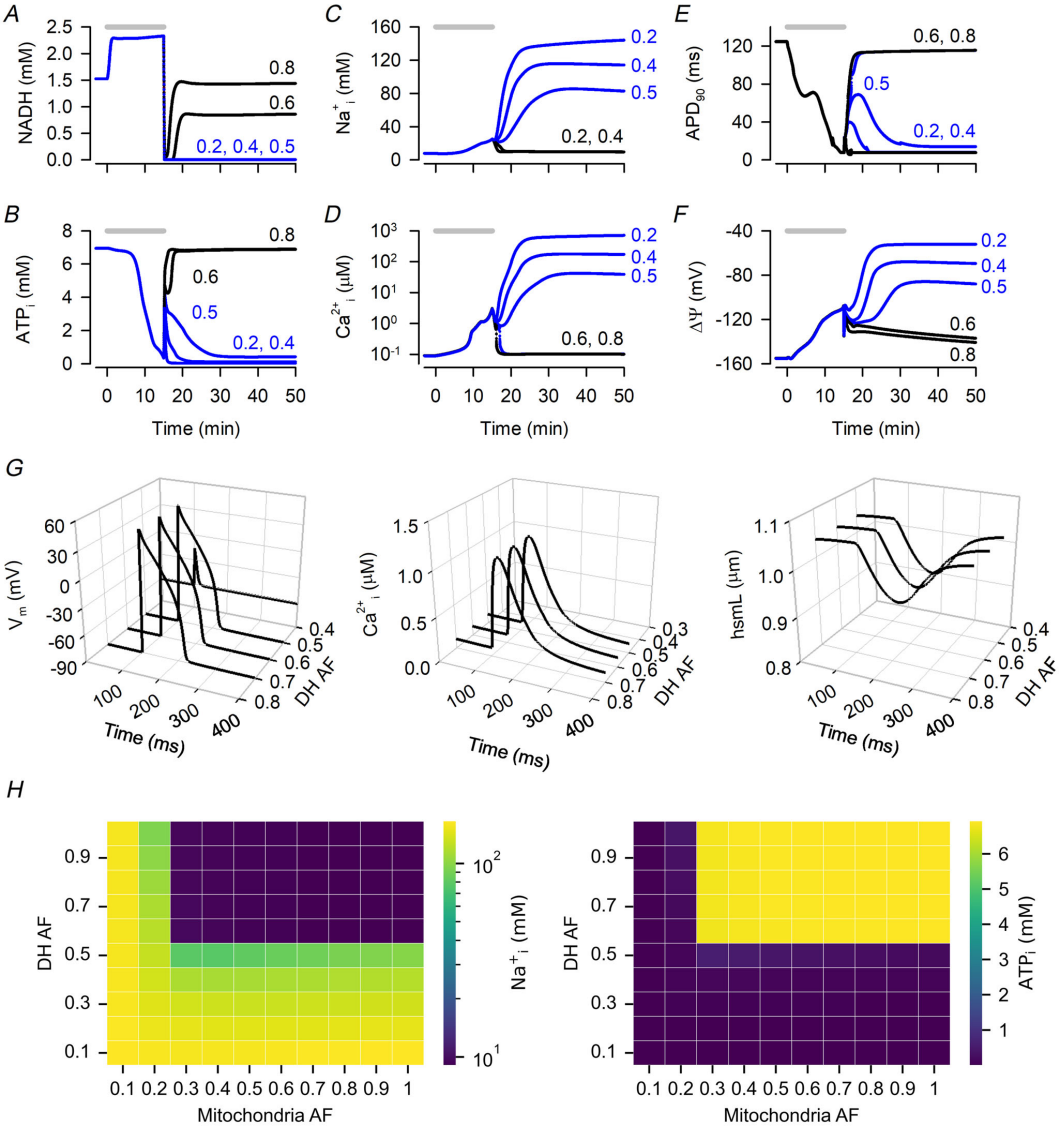
Contribution of NADH production rate on ischaemia–reperfusion injury The rate constant of substrate dehydrogenation (*k*_DH_) was varied upon start of reperfusion after 15 min of ischaemia by multiplying an amplitude factor (DH AF) of 0.1–1.0, whereas amplitudes of all other mitochondrial components were decreased to 50% as in the standard reperfusion protocol. *A*, NADH concentration. *B*, ATP_i_ concentration. *C*, Na^+^_i_ concentration. *D*, Ca^2+^_i_ concentration. *E*, APD_90_. *F* ΔΨ. Values at the end of one cycle are plotted in panels (*A–F*). *G*, configurations of action potential, Ca^2+^_i_ transient and cell shortening as revealed by hsmL (half sarcomere length) during one stimulus cycle at 50 min. Presented data are for DH AF of 0.8, 0.7, 0.6 and 0.5 for *V*_m_, for DH AF of 0.8, 0.7 and 0.6 for Ca^2+^_i_ and hsmL. Note that other traces with lower DH AF were scale overed from graphs. Light‐grey bars indicate the ischaemia period. *H*, heat map plot of Na^+^_i_ (left) and ATP_i_ (right) concentrations at 50 min (end of 32 min of reperfusion) in changing DH AF 0.1–1.0 and mitochondrial amplitude factors other than DH (mitochondria AF) 0.1–1.0. Note that Na^+^_i_ and ATP_i_ concentrations were within normal range when DH AF was set >0.6 for a wide range of mitochondrial AF.

In the standard simulation protocol of reperfusion (Fig. 6), we assumed that the activities of all the mitochondrial components except for H^+^ and Ca^2+^ buffers were damaged to 50% of those in normoxic conditions according to Kuzmiak‐Glancy et al. (2022). The effect of residual DH activity on reperfusion injury is shown in Fig. 12. The rate constant of substrate dehydrogenation (*k*_DH_) was varied at the start of reperfusion with an amplitude factor (DH AF) of 0.2–0.8, whereas DH AF was 0.5 in the standard reperfusion protocol (Fig. 6). Major variables associated with E–C coupling and energy metabolism were restored to the levels in normoxic conditions when *k*_DH_ was maintained close to the control values (DH AF = 0.6–0.8) (Fig. 12*A–F*). The configurations of action potential, Ca^2+^_i_ transient and cell shortening were restored to the normal (Fig. 12*G*; DH A*F* = 0.6, 0.7 and 0.8). The results suggested that DH activity, namely capability of NADH production, during the reperfusion period is an important determinant of reperfusion injury. Then the relation between DH activity and other mitochondrial activities was examined. DH AF was varied 0.1–1.0, with 0.1 step width, and AFs (mitochondria AF) of 0.1–1.0 were multiplied to other mitochondrial components at the start of reperfusion. Na^+^_i_ and ATP_i_ at the end of simulation were plotted on a heat map, respectively (Fig. 12*H*). Clearly maintaining DH activity (DH AF: 0.6–1.0) was effective at mitigating reperfusion injury.

### Direct modulation of Na^+^ flux for mitigating reperfusion injury

**Figure 13:**
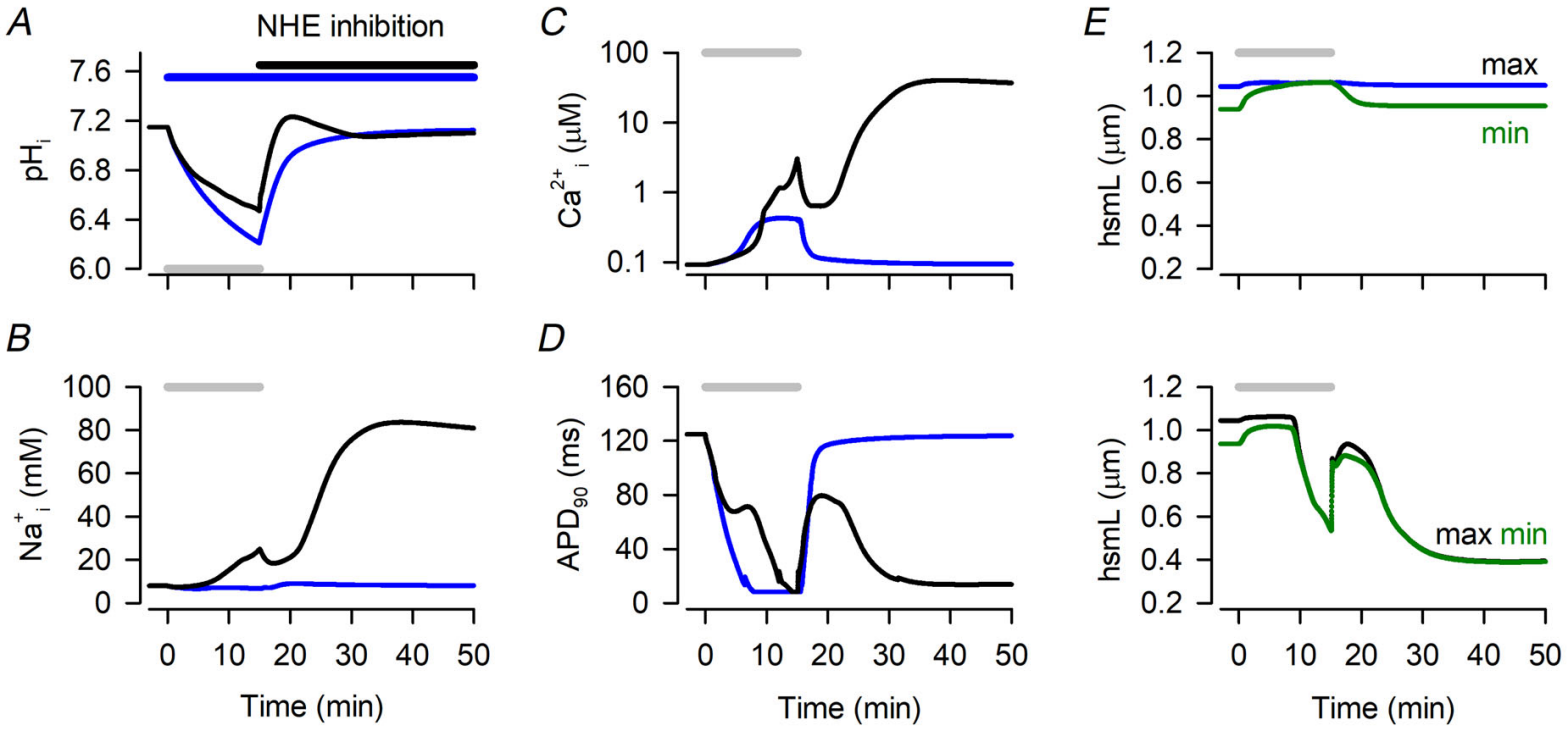
Simulation of the effects of NHE inhibition on ischaemia–reperfusion injury NHE activity was completely suppressed throughout the ischaemia and reperfusion periods (blue lines) or only the reperfusion period (black lines). *A*, pH_i_. *B*, Na^+^_i_ concentration. *C*, minimum Ca^2+^_i_ concentration during one cycle. *D*, APD_90_. *E*, hsmL (half sarcomere length). Upper panel represents inhibition of NHE during the ischaemia and reperfusion periods; lower panel represents inhibition of NHE only for the reperfusion period. Grey bars indicate the ischaemia period.

As demonstrated in the sensitivity analysis on Na^+^_i_ (Fig. 11), NHE and NaK had considerable effects at the end of ischaemia. We next examined whether modulations of these Na^+^ permeable transporters were effective in mitigating reperfusion injury. In the model NHE inhibition throughout the ischaemic and reperfusion periods had beneficial effects in suppressing Na^+^_i_ overload and mitigating reperfusion injury though intracellular acidosis became more severe (Fig. 13, blue lines). The model also demonstrated the ineffectiveness of NHE inhibition applied only during the reperfusion period (Fig. 13, black lines). The ineffectiveness was due to the already low activity of NHE during reperfusion because pH_i_ was recovered (Fig. 6). These results are consistent with the previous simulation study by Roberts and Christini (2011) and reproduce the experimental study showing that treatment of heart with cariporide, an inhibitor of NHE, attenuates ischaemia–reperfusion injury when applied during the ischaemia period, whereas it had no beneficial effects when applied in the early reperfusion period (Klein et al., 2000).

**Figure 14:**
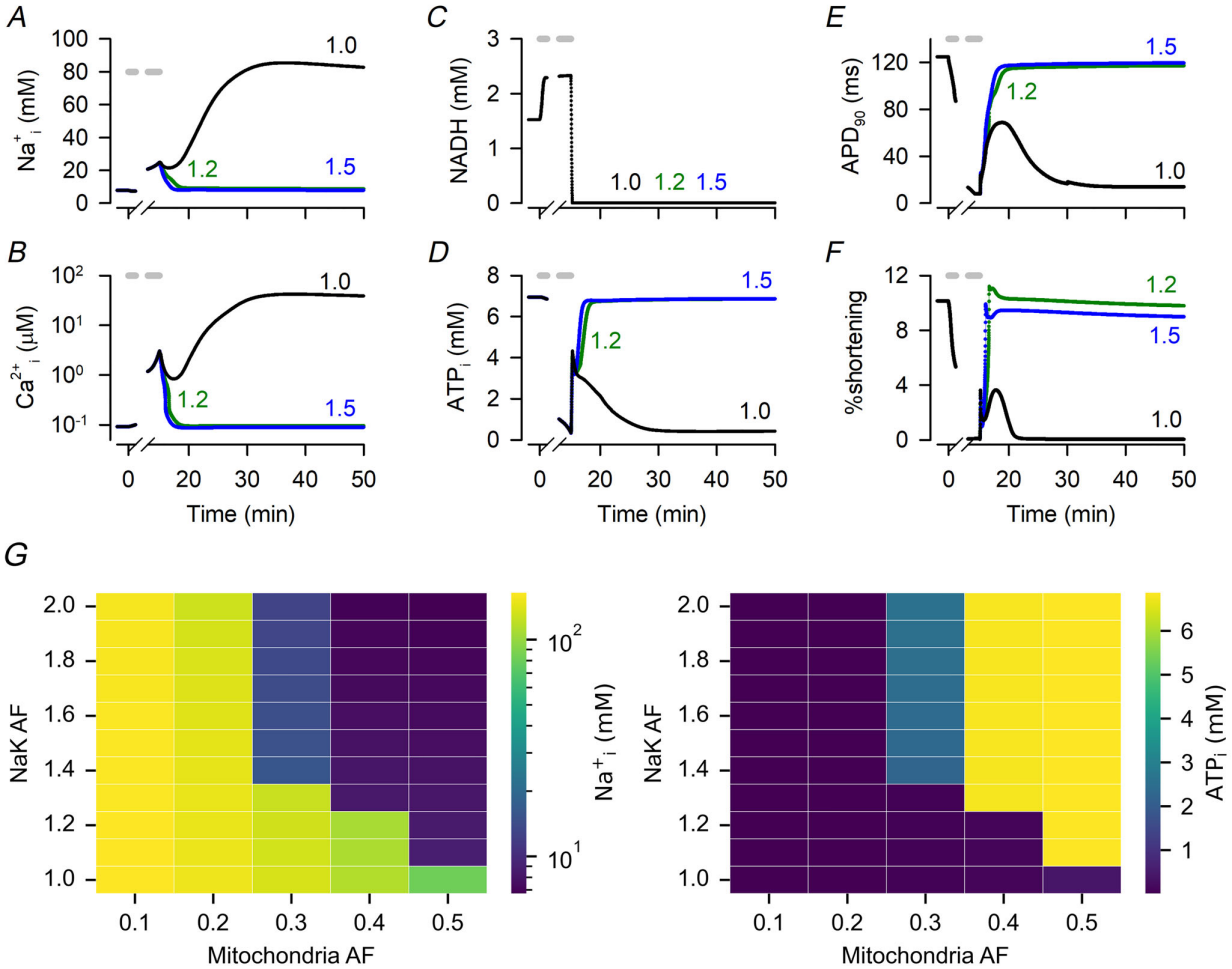
Activation of NaK (Na^+^/K^+^ pump) during reperfusion An amplitude factor of NaK (NaK AF, 1.0–2.0) was multiplied to the NaK current upon the onset of reperfusion in the standard ischaemia–reperfusion protocol. *A*, Na^+^_i_ concentration. *B*, Ca^2+^_i_ concentration. *C*, NADH concentration. *D*, ATP_i_ concentration. *E*, APD_90_. *F*, cell shortening (%). % shortening = (maximalhsmL−minimalhsmL)×100/maximalhsmL. Values at the end of one cycle are plotted in panels (A–F). *G*, Heat map plot of Na^+^_i_ (left) and ATP_i_ (right) concentrations at 50 min (end of 32 min of reperfusion) in changing NaK AF 1.0–2.0 and Mitochondria AF 0.1–0.5. Note that Na^+^_i_ and ATP_i_ concentrations were within normal range when NaK AF was increased even though mitochondrial activity was low (Mitochondria AF 0.4 and 0.5).

The negative values of NaK on sensitivity analysis at normoxia and ischaemia (Fig. 11) suggested that although the sensitivity at reperfusion was low, the activation of NaK upon reperfusion may relieve Na^+^_i_ overload. To test this hypothesis the amplitude of NaK was augmented up to twofold by multiplying the AF (NaK AF, 1.0–2.0) upon the onset of reperfusion (Fig. 14). The increase in NaK activity relieved Na^+^_i_ overload and restored ATP_i_ to the levels in normoxic conditions (Fig. 14). The relieving effect of NaK activation is still effective against severe mitochondria damage (Mitochondria AF 0.4 and partially 0.3).

### Short‐term suppression of ATP consumption for mitigating reperfusion injury

**Figure 15:**
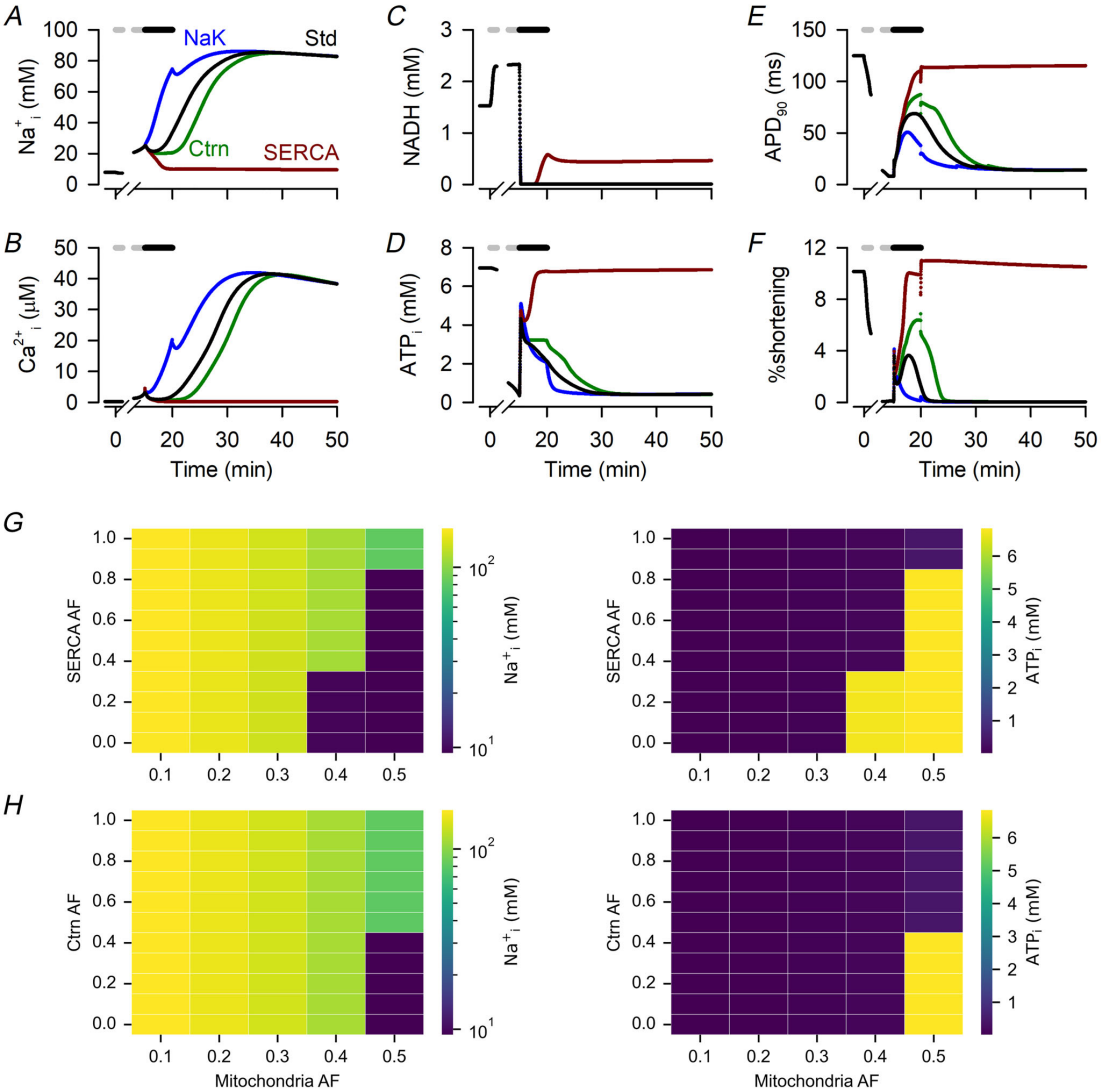
Effects of short‐term suppression of ATP consumption during reperfusion on ischaemia–reperfusion injury Activity of NaK (Na^+^/K^+^ pump), SERCA (sarcoplasmic/endoplasmic reticulum Ca^2+^ pump) or contraction was suppressed by 50% with an amplitude factor of NaK (NaK AF), SERCA (SERCA AF) or contraction (Ctrn AF) of 0.5, respectively, for 5 min after reperfusion (black bars). *A*, Na^+^_i_ concentration. *B*, Ca^2+^_i_ concentration. *C*, NADH concentration. *D*, ATP_i_ concentration. *E*, APD_90_. *F*, cell shortening (%). *G*, heat map plot of Na^+^_i_ (left) and ATP_i_ (right) concentrations at 50 min (end of 32 min of reperfusion) in changing SERCA AF 0.0–1.0 and Mitochondria AF 0.1–0.5. *H*, heat map plot of Na^+^_i_ (left) and ATP_i_ (right) concentrations at 50 min (end of 32 min of reperfusion) in changing Ctrn AF 0.0–1.0 and Mitochondria AF 0.1–0.5. Note that Na^+^_i_ and ATP_i_ concentrations were within the normal range when SERCA AF was decreased even though mitochondrial activity was low (Mitochondria AF 0.4 and 0.5), and suppression of contraction has weak but similar effects.

Because increasing mitochondrial substrate dehydrogenation was effective in mitigating reperfusion injury, a suitable reduction in ATP consumption via NaK, SERCA or contraction during reperfusion may increase ATP_i_ and be effective in mitigating reperfusion injury though this carries the risk of exacerbating reperfusion injury. In Fig. 15*A–F* the activity of NaK, SERCA or contraction was inhibited by 50% for 5 min after the onset of reperfusion. The attenuation of SERCA was effective, and that of contraction had some positive effect in mitigating reperfusion injury, whereas inhibition of NaK worsened the injury. As shown in Fig. 15*G* and *H*, SERCA inhibition was effective against more severe mitochondrial damage, whereas the effect of contraction inhibition was relatively limited.

## Discussion

We aimed to gain insight into the mechanisms underlying myocardial ischaemia–reperfusion injury and to explore effective interventions applicable during reperfusion to mitigate reperfusion injury. Our newly developed integrated model of myocardial excitation‐contraction‐energetics coupling revealed that mitochondrial dysfunction during reperfusion, especially dysfunction of mitochondrial substrate dehydrogenation, is a pivotal factor of reperfusion injury. It was demonstrated that maintaining mitochondrial substrate dehydrogenation, suppressing ATP consumption via SERCA or contraction for a short period or activating NaK potentially mitigates reperfusion injury.

### Ischaemia–reperfusion injury

This simulation analysis revealed the following mechanisms underlying ischaemia–reperfusion injury. During ischaemia a decrease in extracellular O_2_ and cessation of extracellular circulation induce progressive acidosis in the extracellular, intracellular and mitochondrial matrix spaces, leading to activation of NHE, followed by gradual increases in Na^+^_i_ and Ca^2+^_i_. Though total ATP consumption decreases due to acidosis, ATP_i_ concentration decreases because of attenuation of mitochondrial ATP synthesis caused mainly by ΔΨ depolarization, which is facilitated by mPTP opening at the late stage of ischaemia. Contraction ceases relatively early due to PCr depletion, that is, the inhibition of direct ATP supply via creatine kinase, followed by the failure of action potential generation. By the end of the standard 15‐min ischaemia, the mechanical and electrical activities of the model cell completely cease, accompanied by an increase in Na^+^_i_ and Ca^2+^_i_.

On reperfusion recovery of extracellular flow and oxygen supply facilitates the restoration of extracellular and intracellular pH, but mitochondrial function remains impaired. The remaining mitochondrial activity synthesizes ATP by utilizing accumulated ADP and Pi, leading to a partial recovery of ATP_i_ level alongside the near‐complete depletion of NADH, a primary substrate for OXPHOS. If ATP_i_ remained at relatively high levels before reperfusion (short ischaemia) or ATP consumption was relatively low, the model cell recovers to a near‐control state. If ATP_i_ level was relatively low before reperfusion (prolonged ischaemia) or ATP consumption was relatively high (high Na^+^ and Ca^2+^_i_ levels), ATP_i_ decreases again, leading to severe Na^+^ and Ca^2+^_i_ overloads (reperfusion injury).

Preservation of mitochondrial NADH production during reperfusion facilitates ATP synthesis, leading to the activation of NaK and SERCA, which then improves Na^+^_i_ and Ca^2+^_i_ overloads. There are several possibilities to activate mitochondrial substrate dehydrogenation. NADH is produced in mitochondria from NAD^+^ by pyruvate dehydrogenase, citrate cycle (isocitrate dehydrogenase, 2‐oxoglutarate dehydrogenase and malate dehydrogenase) and β‐oxidation (3‐hydroxyacyl‐CoA dehydrogenase) (Walker & Tian, 2018). Activating these enzymes would increase NADH level. The activation of NAD^+^ synthesis by nicotinic acid phosphoribosyltransferase (NAPRT), nicotinamide phosphoribosyltransferase (NAMPT) and amino‐carboxy‐muconate‐semialdehyde decarboxylase (ACMSD) (Yusri et al., 2025) may be effective. Yamamoto et al. (2014) demonstrated that the application of nicotinamide mononucleotide (NMN), a product of NAMPT, just before and during reperfusion protected the heart from ischaemia–reperfusion injury. They concluded that the effect of NMN is mediated through activation of Sirt1, but the effect may be, at least in part, due to conversion of NAD^+^ to NADH.

Suppression of ATP consumption by SERCA or contraction upon reperfusion for a short period potentially mitigates reperfusion injury in this study. To date studies on the effects of manipulating SERCA activity on reperfusion injury have yielded conflicting results. SERCA inhibition in the early stage of reperfusion was suggested to diminish cell damage by preventing mPTP opening (Abdallah et al., 2011), whereas SERCA1a overexpression was reported to be beneficial against reperfusion injury (Talukder et al., 2007). Recently exogenous application of a micropeptide that inhibits SERCA, myoregulin, was reported to attenuate functional impairment caused by ischaemia–reperfusion (Appleby et al., 2024). Because of these conflicting experimental data our computational model provides a mechanistic basis that supports the beneficial role of SERCA inhibition against reperfusion injury. The strategy to attenuate reperfusion injury by suppressing contraction using 2,3‐butanedione monoxime (BDM) has been explored for decades; however its efficacy remains debated, probably due to its non‐selective and protocol‐dependent effects (Armstrong & Ganote, 1991; Habazettl et al., 1998). In the past few years two cardiac myosin‐targeting agents, mavacamten and aficamten, were approved by the U.S. Food and Drug Administration (FDA) for treating hypertrophic cardiomyopathy. Very recently potential efficacy of these drugs on ischaemia–reperfusion injury was reported (Yusof et al., 2026). Our result theoretically supports this experimental result. Collectively our simulation analysis proposes that short‐term suppression of SERCA or contraction upon reperfusion is a promising strategy for mitigating reperfusion injury.

Activation of NaK to relieve Na^+^ overload during reperfusion is also an effective method in this study. Thus far no clear experimental evidence has been found on the protective effect of NaK activation. Zhang et al. reported that a NaK‐activating antibody, DRRSAb, protected the heart against ischaemic injury in both cardiomyocytes and isolated heart (Zhang et al., 2024). However this antibody has a positive inotropic effect, and stimulation of Src/PI3K/Akt/ERK1/2 pathways was proposed for the underlying mechanism. Further studies are needed to examine the protective role of NaK activation on reperfusion injury.

### Advantage of our integrated model for understanding ischaemia–reperfusion injury

With respect to pH_i_ regulation our integrated model of cardiac excitation‐contraction‐energetics coupling has several unique characteristics. Firstly, H^+^ fluxes between extracellular space and cytosol, and between cytosol and mitochondria, are calculated. Secondly, H^+^ production and consumption by ATPases, mitochondrial ATP synthase and metabolism are included. Thirdly, H^+^ transport by OXPHOS is included. Because OXPHOS is driven by H^+^ gradient and ΔΨ across the mitochondrial inner membrane, precise numerical reproduction of H^+^ fluxes across sarcolemma and across the mitochondrial inner membrane as well as reproduction of ΔΨ is inevitable to simulate ischaemia–reperfusion injury. Thus far no such mathematical model has been reported. Finally, pH_i_ dependence of major proteins responsible for E–C coupling, such as RyR, SERCA, NCX, NaK and contraction (Fig. 2), was included. To the best of our knowledge, this is the most comprehensive mathematical model, although not perfect, of cardiac excitation‐contraction‐energetics coupling and pH regulation.

### Limitations of the study

This study has several limitations. Firstly, a detailed process of mitochondrial NADH production and NAD^+^ synthesis is not involved in this model. We opted for a simpler model as our initial step instead. It would be possible to identify specific processes of substrate dehydrogenation by incorporating each process of citrate cycle (Saito et al., 2016; Takeuchi & Matsuoka, 2025), β‐oxidation (Cortassa et al., 2017; van Eunen et al., 2013) and detailed glycolysis (Hashemzadeh et al., 2020). Secondly, because this model is a model of single cardiomyocyte, heterogeneity of ischaemic and subischaemic regions is not considered. Tissue‐level simulation is beyond the scope of this study. For the same reason coronary blood flow and myocardial perfusion (Sturgess et al., 2024) were not incorporated. Finally, ROS metabolism and ROS‐induced effects are not implemented in this model. Though accumulated experimental evidences have demonstrated that ROS produced within mitochondria considerably contribute to ischaemia–reperfusion injury (Bugger & Pfeil, 2020; Cadenas, 2018), detailed mechanisms of ROS‐mediated ischaemia–reperfusion injury are still under research, and there are several controversial points, for example, sites of ROS production (Bugger & Pfeil, 2020; Cadenas, 2018).

## Additional information

## Competing interests

None declared.

## Author contributions

T.S.: methodology, software, validation, formal analysis, data curation, funding acquisition. A.T.: methodology, data curation, writing – original draft, writing – review and editing, funding acquisition. S.M.: conceptualization, methodology, software, validation, formal analysis, investigation, resources, data curation, writing – original draft, writing – review and editing, visualization, funding acquisition. All authors approved the final version of the manuscript and agreed to be accountable for all aspects of the work in ensuring that questions related to the accuracy or integrity of any part of the work are appropriately investigated and resolved, and confirmed that all persons designated as authors qualify for authorship, and all those who qualify for authorship are listed.

## Funding

This study was supported by JSPS KAKENHI grant numbers 20K12064 (T.S.), 22K06841 (A.T.) and 22H02803 (S.M.), and by the ‘Initiative for Realizing Diversity in the Research Environment’ from MEXT (A.T.).

## Author's present address

S. Matsuoka: Department of Community Health Science, Faculty of Medical Sciences, University of Fukui, 23‐3 Matsuokashimoaizuki, Eiheiji‐cho, Yoshida‐gun, Fukui 910‐1193, Japan.

## Generative AI statement

No generative AI tools were used in the preparation of this manuscript.

## Supporting information




Peer Review History

Supporting Information

## Data Availability

The source code is available at https://github.com/atakeuti/TS‐AT‐SM‐2026‐IRmodel. The original contributions presented in the study are included in the article and in the Supplementary Material; further inquiries can be directed to the corresponding author.

## Abbreviations

ACMSD, amino‐carboxy‐muconate‐semialdehyde decarboxylase; ADP_i_, total cytosolic ADP; AE, sarcolemmal Cl^−^‐HCO_3_^−^ exchange (anion exchange); AF, amplitude factor; AK, adenylate kinase; ANT, adenine nucleotide antiport; APD_90_, action potential duration at 90% repolarization; ATP_i_, total cytosolic ATP; ATP_Mg_, cytosolic ATP bound to Mg^2+^; C1, complex I; C3, complex III; C4, complex IV; Ca^2+^_i_, cytosolic Ca^2+^; Caleak_SR_, Ca^2+^ leak from SR to cytosol; Ca^2+^_mit_, mitochondrial Ca^2+^; CaUni, Ca^2+^ uniporter; CHE, sarcolemmal Cl^−^‐OH^−^ exchange; CK, creatine kinase; Cntr, contraction; CO_2o_, extracellular CO_2_; CO_2_hyd, CO_2 _hydration reaction; DH, mitochondrial substrate dehydrogenation; dpH, pH difference between mitochondria and cytosol; ΔΨ, mitochondrial membrane potential; E–C, excitation–contraction; *F*, Faraday's constant; Gly, glycolysis; Hleak, proton leak; hsmL, half sarcomere length; *I*_CaL_, L‐type Ca^2+^ current; *I*_Cab_, background Ca^2+^ current; *I*_CaT_, T‐type Ca^2+^ current; *I*cCl, total Cl^−^ current; *I*cK, total K^+^ current; *I*_Clb_, background Cl^−^ current; *I*cNa, total Na^+^ current; *I*_K1_, inward rectifier K^+^ current; *I*_KATP_, ATP‐sensitive K^+^ current; *I*_Kpl_, voltage‐dependent K^+^ current; *I*_Kr_, delayed rectifier K^+^ current, rapid component; *I*_Ks_, delayed rectifier K^+^ current, slow component; *I*_l(Ca)_, Ca^2+^‐activated background cation current; *I*_Na_, Na^+^ current; *I*_NaL_, late Na^+^ current; *I*_NSC_, background non‐selective cation current; *I*_to_, transient outward current; *J*_AE_, ion flux of anion exchange; *J*_CHE_, ion flux of sarcolemmal Cl^−^‐OH^−^ exchange; *J*_CO2_, diffusive flux of CO_2_ across sarcolemmal membrane; *J*_NBC_, ion flux of sarcolemmal Na^+^‐HCO_3_^−^ cotransporter; *J*_NHE_, ion flux of sarcolemmal Na^+^‐H^+^ exchange; *J*_NKCC1_, ion flux of Na^+^/K^+^/2Cl^−^ cotransporter 1; *k*_DH_, rate constant of substrate dehydrogenation; KHE, K^+^‐H^+^ exchange; K^+^_o_, extracellular K^+^; KUni, K^+^ uniporter; mPTP, mitochondrial permeability transition pore; Na^+^_i_, cytosolic Na^+^; NaK, Na^+^/K^+^ pump; NAMPT, nicotinamide phosphoribosyltransferase; NAPRT, nicotinic acid phosphoribosyltransferase; NBC, sarcolemmal Na^+^‐HCO_3_^−^ cotransporter; NCX, sarcolemmal Na^+^‐Ca^2+^ exchange; NCX_mit_, mitochondrial Na^+^‐Ca^2+^ exchange; NHE, sarcolemmal Na^+^‐H^+^ exchanger; NHE_mit_, mitochondrial Na^+^/H^+^ exchange; NKCC1, Na^+^/K^+^/2Cl^−^ cotransporter 1; NMN, nicotinamide mononucleotide; O_2o_, extracellular O_2_; [O_2_]_o_i_, extracellular oxygen concentration at the end of ischaemia; OXPHOS, mitochondrial oxidative phosphorylation; PCr, phosphocreatine; pH_i_, cytosolic pH; pH_mit_, mitochondrial pH; pH_o_, extracellular pH; Pi, inorganic phosphate; PiC, phosphate translocator; PMCA, plasma membrane Ca^2+^‐ATPase, *P*(O)_mPTP_, probability of mitochondrial permeability transition pore in open state; ROS, reactive oxygen species; RyR, ryanodine receptor; SERCA, sarcoplasmic/endoplasmic reticulum Ca^2+^ pump; SN, ATP synthase; T, free troponin C; T^*^, free troponin C with crossbridge attached; TCa, troponin C bound to Ca^2+^; TCa^*^, troponin C bound to Ca^2+^ with crossbridge attached; τ_i_, time constants for extracellular oxygen decrease during ischaemia; τ_r_, time constants for extracellular oxygen recovery during reperfusion; *t*_r_, time when reperfusion begins; *v*_C1_, rate of complex I; *v*_DH_, rate of substrate dehydrogenation; *V*_m_, membrane potential; *V*_o_, extracellular volume
