# Supplementary material for: Integrative simulation analysis of myocardial ischaemia–reperfusion injury
Source: J Physiol. 2026 Aug 20;604(18):7640–62. doi: 10.1113/JP290440 (PMC13577699; doi:10.1113/JP290440)
Supplement: Supplementary file 1 [file TJP-604-7640-s001.pdf]

Online Supplementary Material

Integrative simulation analysis of myocardial ischemia-reperfusion injury

Takao Shimayoshi, Ayako Takeuchi, Satoshi Matsuoka

I. Model equations

Constant and Abbreviations

R	Gas constant, 8.3143 C mV K^{-1} $mmol^{-1}$
T	Absolute temperature, 310.15 K
F	Faraday's constant, 96.4867 C $mmol^{-1}$
C_m	Membrane capacitance, 211.2 pF
P_x	A convert factor (pA pF^{-1} mM^{-1}) for ion x in Goldman–Hodgkin–Katz flux equation
G_X	Conductance (pA mV^{-1})
V_m	Membrane potential (mV)
E_K	Equilibrium potential for K^+ (mV) $= \frac{R \cdot T}{F} \cdot \ln \frac{[K^+]_o}{[K^+]_i}$
E_{Na}	Equilibrium potential for Na^+ (mV) $= \frac{R \cdot T}{F} \cdot \ln \frac{[Na^+]_o}{[Na^+]_i}$
$P(X)_Y$	Probability of state X in a multiple state transition model of Y
CF_X	Dependency of ion flux on V_m (constant field equation)
$[X_{tot}]$	Total concentration of X (mM)
$[X]_i$	Intracellular concentration of substance X (mM)
$[X]_o$	Extracellular concentration of substance X (mM)
$[X]_{mit}$	Mitochondrial concentration of substance X (mM)
$[Mg^+]_i$	Cytosolic Mg^{2+} concentration, 1.145 mM
$[SPM]_i$	Cytosolic spermine concentration, 0.005 mM

Extracellular space

Volume		
	Model adjusted based on Scully et al. (Scully *et al.*, 2018)	
	V_o (μm^3)	Volume of the restricted extracellular space (μm^3)<br>$V_o = Vcell \cdot 0.34$ during ischemia and 0 during normoxia and reperfusion.
Ions and gasses		
	Adopted from Kuzumoto et al.(Kuzumoto *et al.*, 2008) and Crampin and Smith (Crampin & Smith, 2006).	
	$[Na^+]_o$	Extracellular Na^+ concentration, 140. 0 mM
	$[K^+]_o$	Extracellular K^+ concentration, 5.4 mM

	$[Ca^{2+}]_o$	Extracellular Ca^{2+} concentration, 1.8 mM
	$[Cl^-]_o$	Extracellular Cl^- concentration, 140.0 mM
	$[HCO_3^-]_o$	Extracellular HCO_3^- concentration, 23.5542 mM
	pH_o	Extracellular pH
	$[H^+]_o$	Extracellular H^+ concentration (mM)
	$[CO_2]_o$	Extracellular CO_2 concentration (mM)
	α_{Co2}	Solubility of CO_2 at atmospheric pressure of 760 mmHg, 0.03253 mM $mmHg^{-1}$
	$[O_2]_o$	Extracellular O_2 concentration (mM), 0.24 mM. See "Simulation of Ischemia and reperfusion" section for ischemia and reperfusion protocol.
$pH_o = pK_a + log\left(\frac{[HCO_3^-]_o}{[CO_2]_o}\right), \quad pK_a = 6.12$<br>$[CO_2]_o = \alpha_{Co2} \cdot 760 \cdot \frac{5}{100} \quad = 1.236, assuming\ 5\%\ CO_2$<br>$[H^+]_o = 10^{-pH_o} \cdot 1000$		

Cell volume

Adapted from Kuzumoto et al. (Kuzumoto *et al.*, 2008)		
	V_{cell}	Cell volume, 100.0 · 20.0 · 8.0 = 16,000 μm^3
	V_i	Cytosolic volume, $V_{cell} \cdot 0.8$ μm^3
	V_{jSR}	Volume of junctional SR, $V_{cell} \cdot 0.02$ μm^3
	V_{nSR}	Volume of network SR, $V_{cell} \cdot 0.05$ μm^3
	V_{mit}	Volume of mitochondria, $V_{cell} \cdot 0.23$ μm^3
	R_{mc}	Ratio of mitochondrial volume to cytosolic volume, $= \frac{V_{mit}}{V_i}$
	A_i	Cell surface area, $(100.0 \cdot 20.0 + 20.0 \cdot 8.0 + 100.0 \cdot 8.0) \cdot 2 = 5{,}920\ \mu m^2$

Ca^{2+}-binding proteins

CaM : Calmodulin		
	Adopted from Kuzumoto et al. (Kuzumoto *et al.*, 2008).	
	K_dCaM	Dissociation constant of calmodulin, $2.38 \cdot 10^{-3}$ mM
	$B_{max}CaM$	Total amount of calmodulin, 0.05 mM
TroponinC		
	Adopted from Kuzumoto et al. (Kuzumoto *et al.*, 2008).	
	$TrnTotal$	Total amount of TroponinC, 0.07 mM
CSQN : Calsequestrin		
	Adopted from Kuzumoto et al. (Kuzumoto *et al.*, 2008).	
	K_dCSQN	Dissociation constant of calsequestrin, 0.8 mM

	$B_{max}CSQN$	Total amount of calsequestrin, 10 mM

Goldman–Hodgkin–Katz flux equation

	Adopted from Kuzumoto et al. (Kuzumoto *et al.*, 2008)
	Ion current (pA/pF) is expressed as $I = P_x \cdot CF_x$, where (P_x is a convert factor (pA/pF/mM) for ion x, and *CFx* is determined Goldman-Hodgkin-Katz flux equation (in mM)
	For ions exist in cytosol and extracellular space,<br>$CF_X = \frac{z_X \cdot F \cdot V_m}{R \cdot T} \frac{([X]_i - [X]_o \cdot e^{\left(-\frac{z_X \cdot F \cdot V_m}{R \cdot T}\right)})}{(1 - e^{\left(-\frac{z_X \cdot F \cdot V_m}{R \cdot T}\right)})}$<br>For ions exist in cytosol and mitochondria,<br>$CFmit_X = \frac{z_X \cdot F \cdot dPsi}{R \cdot T} \frac{([X]_{mit} - [X]_i \cdot e^{\left(-\frac{z_X \cdot F \cdot dPsi}{R \cdot T}\right)})}{(1 - e^{\left(-\frac{z_X \cdot F \cdot dPsi}{R \cdot T}\right)})}$<br>$z_X =$ the valence of ion X

Membrane currents: Inward currents

I_{Na} : Na^+ current (pA pF^{-1})		
	Adopted from Kuzumoto et al. (Kuzumoto *et al.*, 2008).	
	$I_{Na}Na$	Na^+ ion current through I_{Na}
	$I_{Na}K$	K^+ ion current through I_{Na}
	Voltage-dependent gate	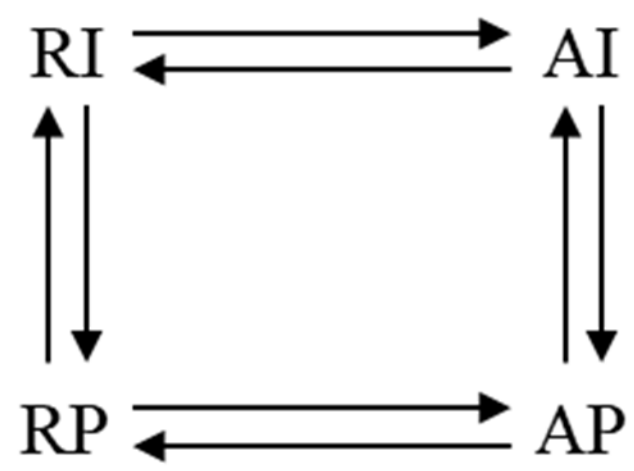
	$P_{INa} = 21.67$<br>$I_{Na}Na = P_{INa} \cdot CF_{Na} \cdot P(AP)_{INa} \cdot P(Is)_{INa}$<br>$I_{Na}K = 0.1 \cdot P_{INa} \cdot CF_K \cdot P(AP)_{INa} \cdot P(Is)_{INa}$<br>$I_{Na} = I_{Na}Na + I_{Na}K$<br>$k_{RP,AP} = \frac{1.0}{0.1027 \cdot e^{\left(-\frac{V_m}{8.0}\right)} + 0.25 \cdot e^{\left(-\frac{V_m}{50.0}\right)}}$<br>$k_{AP,RP} = \frac{1.0}{26.0 \cdot e^{\left(\frac{V_m}{17.0}\right)} + 0.02 \cdot e^{\left(\frac{V_m}{800.0}\right)}}$<br>$k_{AP,AI} = \frac{1.0}{0.8 \cdot e^{\left(-\frac{V_m}{400.0}\right)}}$<br>$k_{AI,AP} = 0.0000875$	

	$k_{AI,RI} = \frac{1.0}{1300 \cdot e^{\left(\frac{V_m}{20.0}\right)} + 0.04 \cdot e^{\left(\frac{V_m}{800.0}\right)}}$<br>$k_{RI,AI} = \frac{1.0}{0.0001027 \cdot e^{\left(-\frac{V_m}{8.0}\right)} + 5.0 \cdot e^{\left(-\frac{V_m}{400.0}\right)}}$<br>$k_{RP,RI} = \frac{0.01}{1.0 + \frac{k_{AI,AP} \cdot k_{AP,RP} \cdot k_{RI,AI}}{k_{AP,AI} \cdot k_{RP,AP} \cdot k_{AI,RI}}}$<br>$k_{RI,RP} = 0.01 - k_{RP,RI}$<br>$P(RI)_{INa} = 1.0 - P(RP)_{INa} - P(AP)_{INa} - P(AI)_{INa}$<br>$\frac{\mathrm{d}P(AP)_{INa}}{\mathrm{dt}} = k_{RP,AP} \cdot P(RP)_{INa} - \left(k_{AP,RP} + k_{AP,AI}\right) \cdot P(AP)_{INa} + k_{AI,AP} \cdot P(AI)_{INa}$<br>$\frac{\mathrm{d}P(RP)_{INa}}{\mathrm{dt}} = k_{AP,RP} \cdot P(AP)_{INa} - \left(k_{RP,AP} + k_{RP,RI}\right) \cdot P(RP)_{INa} + k_{RI,RP} \cdot P(RI)_{INa}$<br>$\frac{\mathrm{d}P(AI)_{INa}}{\mathrm{dt}} = k_{AP,AI} \cdot P(AP)_{INa} - \left(k_{AI,AP} + k_{AI,RI}\right) \cdot P(AI)_{INa} + k_{RI,AI} \cdot P(RI)_{INa}$
	Ultra-slow gate: $(1-\mathrm{P(Is)}) \underset{\beta_{si}}{\overset{\alpha_{si}}{\rightleftharpoons}} \mathrm{P(Is)}$
	$\alpha_{si} = \frac{1.0}{9000000000.0 \cdot e^{\left(\frac{V_m}{5.0}\right)} + 8000.0 \cdot e^{\left(\frac{V_m}{100.0}\right)}}$<br>$\beta_{si} = \frac{1.0}{0.014 \cdot e^{\left(-\frac{V_m}{5.0}\right)} + 4000.0 \cdot e^{\left(-\frac{V_m}{100.0}\right)}}$<br>$\frac{\mathrm{d}P(Is)_{INa}}{dt} = \alpha_{si} \cdot (1 - P(Is)_{INa}) - \beta_{si} \cdot P(Is)_{INa}$
I_{NaL} : late Na^{+} current (pA pF^{-1})	
	Adapted from Gaur et al. (Gaur *et al.*, 2009). Rate constants, α and β, were decreased to increase the amplitude.
	$I_{NaL}Na$ — Na^{+} ion current through I_{NaL}
	$P_{Na} = 0.00007$<br>$I_{NaL} = P_{INaL} \cdot P\left(INaL_y\right)^3 \cdot (V_m - E_{Na})$<br>$I_{NaL}Na = I_{NaL}$<br>$\alpha = 1.9 \cdot e^{\left(\frac{V_m}{16.5}\right)}$<br>$\beta = 0.02 \cdot e^{\left(-\frac{V_m}{20.0}\right)}$<br>$\frac{\mathrm{d}P(y)_{INaL}}{dt} = \alpha \cdot (1 - P(y)_{INaL}) - \beta \cdot P(y)_{INaL}$
I_{CaL} : L-type Ca^{2+} current (pA pF^{-1})	
	Adapted from Kuzumoto et al. (Kuzumoto *et al.*, 2008): PKA activation is omitted.
	$I_{CaL}Ca$ — Ca^{2+} ion current through I_{CaL}
	$I_{CaL}Na$ — Na^{+} ion current through I_{CaL}

	$I_{CaL}K$	K^+ ion current through I_{CaL}
	$ISOfactor_{ICaL}$	β adrenergic receptor activation factor for I_{CaL}
	$ATPfacotr_{ICaL}$	ATP activation factor
	$P_{CaL} = 45.0$<br>$V_{shift} = 0$<br>$ISOfactor_{ICaL} = 1.0$<br>$ATPfacotr_{ICaL} = \frac{1.0}{1.0 + \left(\frac{1.4}{[ATPitot]}\right)^3}$<br>$P(OpenCaL) = P(AP)_{ICaL} \cdot (P(U)_{ICaL} + P(UCa)_{ICaL}) \cdot P(si)_{ICaL}$<br>$I_{CaL}Ca = P_{CaL} \cdot CF_{Ca} \cdot P(OpenCaL) \cdot ATPfacotr_{ICaL} \cdot ISOfactor_{ICaL}$<br>$I_{CaL}Na = 0.0000185 \cdot P_{CaL} \cdot CF_{Na} \cdot P(OpenCaL) \cdot ATPfacotr_{ICaL} \cdot ISOfactor_{ICaL}$<br>$I_{CaL}K = 0.000365 \cdot P_{CaL} \cdot CF_K \cdot P(OpenCaL) \cdot ATPfacotr_{ICaL} \cdot ISOfactor_{ICaL}$<br>$I_{CaL} = I_{CaL}Ca + I_{CaL}Na + I_{CaL}K$	
	Voltage-dependent gate<br>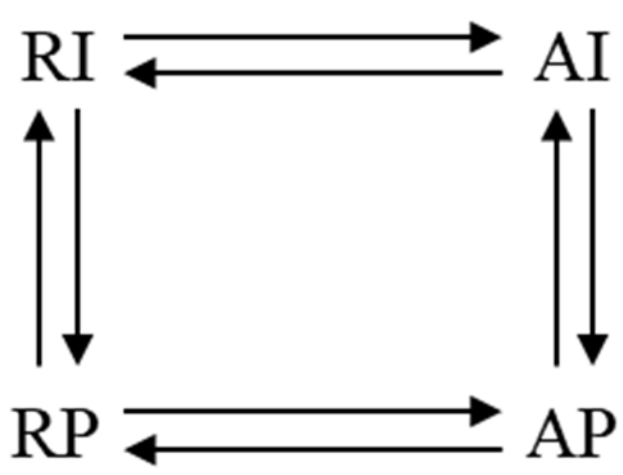 	
	$k_{RP,AP} = \frac{1.0}{0.27 \cdot e^{\left(-\frac{(V_m - V_{shift})}{5.9}\right)} + 1.5 \cdot e^{\left(-\frac{(V_m - V_{shift})}{65.0}\right)}}$<br>$k_{AP,RP} = \frac{1.0}{480.0 \cdot e^{\left(\frac{(V_m - V_{shift})}{7.0}\right)} + 2.2 \cdot e^{\left(\frac{(V_m - V_{shift})}{65.0}\right)}}$<br>$k_{AP,AI} = 0.004$<br>$k_{AI,AP} = 0.001$<br>$k_{AI,RI} = \frac{1.0}{2200000 \cdot e^{\left(\frac{(V_m - V_{shift})}{7.4}\right)} + 11.0 \cdot e^{\left(\frac{(V_m - V_{shift})}{100.0}\right)}}$<br>$k_{RI,AI} = \frac{1.0}{0.0018 \cdot e^{\left(-\frac{(V_m - V_{shift})}{7.4}\right)} + 2.0 \cdot e^{\left(-\frac{(V_m - V_{shift})}{100.0}\right)}}$<br>$k_{RP,RI} = \frac{0.04}{1 + \frac{k_{AI,AP} \cdot k_{AP,RP} \cdot k_{RI,AI}}{k_{AP,AI} \cdot k_{RP,AP} \cdot k_{AI,RI}}}$<br>$k_{RI,RP} = 0.04 - k_{RP,RI}$<br>$P(RI)_{ICaL} = 1 - \mathrm{P}(RP)_{ICaL} - \mathrm{P}(AP)_{ICaL} - \mathrm{P}(AI)_{ICaL}$<br>$\frac{\mathrm{d}P(AP)_{ICaL}}{\mathrm{dt}} = k_{RP,AP} \cdot P(RP)_{ICaL} - \left(k_{AP,RP} + k_{AP,AI}\right) \cdot P(AP)_{ICaL} + k_{AI,AP} \cdot P(AI)_{ICaL}$	

$$\frac{\mathrm{d}P(RP)_{ICaL}}{\mathrm{dt}} = k_{AP,RP} \cdot P(AP)_{ICaL} - \left(k_{RP,AP} + k_{RP,RI}\right) \cdot P(RP)_{ICaL} + k_{RI,RP} \cdot P(RI)_{ICaL}$$

$$\frac{\mathrm{d}P(AI)_{ICaL}}{\mathrm{dt}} = k_{AP,AI} \cdot P(AP)_{ICaL} - \left(k_{AI,AP} + k_{AI,RI}\right) \cdot P(AI)_{ICaL} + k_{RI,AI} \cdot P(RI)_{ICaL}$$

Ca^{2+}-dependent gate

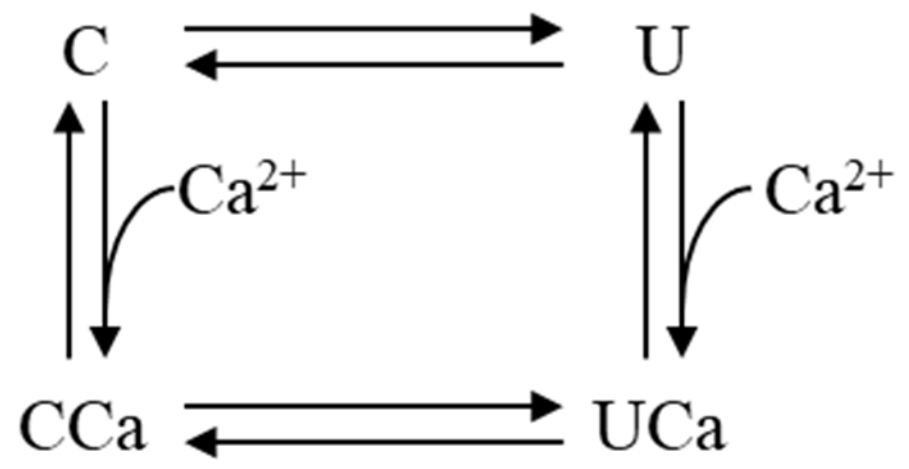

$k_{C,U} = 0.143$

$k_{U,C} = 0.35$

$k_{CCa,UCa} = 0.0003$

$k_{UCa,CCa} = 0.35$

$k_{C,CCa} = 6.954$

$k_{C,CaC} = 0.0042$

$k_{U,UCa} = k_{C,CCa}$

$$k_{UCa,U} = k_{CCa,C} \cdot k_{C,U} \cdot \frac{k_{UCa,CCa}}{k_{U,C} \cdot k_{CCa,UCa}}$$

$\mathrm{CaBindingSiste} = ([Ca^{2+}]_i - 0.3 \cdot 0.0676 \cdot CF_{Ca})$

$\mathrm{CaEffU} = \mathrm{CaBindingSiste} \cdot P(AP)_{ICaL} + [Ca^{2+}]_i \cdot (1.0 - P(AP)_{ICaL})$

$\mathrm{CaEffC} = \mathrm{CaBindingSiste} \cdot P(AP)_{ICaL}$

$P(CCa)_{ICaL} = 1 - P(U)_{ICaL} - P(UCa)_{ICaL} - P(C)_{ICaL}$

$$\frac{\mathrm{d}P(U)_{ICaL}}{\mathrm{dt}} = k_{C,U} \cdot P(C)_{ICaL} - \left(k_{U,C} + k_{U,UCa} \cdot \mathrm{CaEffU}\right) \cdot P(U)_{ICaL} + k_{UCa,U} \cdot P(UCa)_{ICaL}$$

$$\frac{\mathrm{d}P(UCa)_{ICaL}}{\mathrm{dt}} = k_{U,UCa} \cdot \mathrm{CaEffU} \cdot P(U)_{ICaL} - \left(k_{UCa,U} + k_{UCa,CCa}\right) \cdot P(UCa)_{ICaL} + k_{CCa,UCa} \cdot P(CCa)_{ICaL}$$

$$\frac{\mathrm{d}P(C)_{ICaL}}{\mathrm{dt}} = k_{U,C} \cdot P(U)_{ICaL} - \left(k_{C,U} + k_{C,CCa} \cdot \mathrm{CaEffC}\right) \cdot \mathrm{P(C)_{ICaL}} + k_{CCa,C} \cdot P(CCa)_{ICaL}$$

Ultra-slow gate

$$(1-\mathrm{P(Is)}) \underset{\beta_{si}}{\overset{\alpha_{si}}{\rightleftharpoons}} \mathrm{P(Is)}$$

$$\alpha_{si} = \frac{1.0}{250000.0 \cdot e^{\left(\frac{V_m}{9.0}\right)} + 58.0 \cdot e^{\left(\frac{V_m}{65.0}\right)}}$$

$$\beta_{si} = \frac{1.0}{1800.0 \cdot e^{\left(-\frac{V_m}{14.0}\right)} + 66.0 \cdot e^{\left(-\frac{V_m}{65.0}\right)}}$$

	$$\frac{\mathrm{d}P(Is)_{ICaL}}{\mathrm{dt}} = \alpha_{si} \cdot (1 - P(Is)_{ICaL}) - \beta_{si} \cdot P(Is)_{ICaL}$$

I_{CaT} : T-type Ca^{2+} current (pA pF^{-1})		
	Adopted from Kuzumoto et al. (Kuzumoto *et al.*, 2008).	
	$I_{CaT}Ca$	Ca^{2+} ion current through I_{CaT}
	$P_{CaT} = 4.636$<br>$I_{CaT} = P_{CaT} \cdot CF_{Ca} \cdot P(y_1)_{ICaT} \cdot P(y_2)_{ICaT}$<br>$I_{CaT}Ca = I_{CaT}$<br>$\alpha_{y1} = \frac{1.0}{0.019 \cdot e^{\left(-\frac{V_m}{5.6}\right)} + 0.82 \cdot e^{\left(-\frac{V_m}{250.0}\right)}}$<br>$\beta_{y1} = \frac{1.0}{40.0 \cdot e^{\left(\frac{V_m}{6.3}\right)} + 1.5 \cdot e^{\left(\frac{V_m}{10000}\right)}}$<br>$\alpha_{y2} = \frac{1.0}{62000 \cdot e^{\left(\frac{V_m}{10.1}\right)} + 30.0 \cdot e^{\left(\frac{V_m}{3000.0}\right)}}$<br>$\beta_{y2} = \frac{1.0}{0.0006 \cdot e^{\left(-\frac{V_m}{6.7}\right)} + 1.2 \cdot e^{\left(-\frac{V_m}{25.0}\right)}}$<br>$\frac{\mathrm{d}P(y_1)_{ICaT}}{\mathrm{dt}} = \alpha_{y1} \cdot (1.0 - P(y_1)_{ICaT}) - \beta_{y1} \cdot P(y_1)_{ICaT}$<br>$\frac{\mathrm{d}P(y_2)_{ICaT}}{\mathrm{dt}} = \alpha_{y2} \cdot (1.0 - P(y_2)_{ICaT}) - \beta_{y2} \cdot P(y_2)_{ICaT}$	

Membrane currents: Outward currents

I_{K1} : inward rectifier K^+ current (pA pF^{-1})		
	Adopted from Kuzumoto et al. (Kuzumoto *et al.*, 2008).	
	G_{IK1}	Conductance of I_{K1}
	A_{Kdep}	Extracellular K^+-dependent factor
	$P(OpenMode1)$	Open probability of mode1
	$P(OpenMode2)$	Open probability of mode2
	$P(O)_{IK1}$	Open Probability of I_{K1} in open state
	α_{Mg}	Rate constant of Mg^{2+} unbinding (ms^{-1})
	β_{Mg}	Rate constant of Mg^{2+} binding ($mM^{-1}ms^{-1}$)
	$fracO$	Mg^{2+}–blocked fraction
	$fracB$	Mg^{2+}–unblocked fraction
	α_{SPM}	Rate constant of SPM binding (ms^{-1})
	β_{SPM}	Rate constant of SPM unbinding ($\mu M^{-1}ms^{-1}$)
	$I_{K1}K$	K^+ ion current through I_{K1}
	$I_{K1} = G_{IK1} \cdot (V_m - E_K) \cdot (P(OpenMode1) + P(OpenMode2))$<br>$I_{K1}K = I_{K1}$	

$$A_{Kdep} = \frac{\left(\frac{[K^+]_o}{5.4}\right)^{0.6}}{1 + e^{\left(-\frac{([K^+]_o - 3.1)}{0.6}\right)}}$$

$$G_{IK1} = 2.6 \cdot A_{Kdep}$$

Mg^{2+} block

$$\alpha_{Mg} = 12.0 \cdot e^{(-0.025 \cdot (V_m - E_K))}$$

$$\beta_{Mg} = 28.0 \cdot [Mg^{2+}]_i \cdot e^{(0.025 \cdot (V_m - E_K))}$$

$$fracO = \frac{\alpha_{Mg}}{(\alpha_{Mg} + \beta_{Mg})}$$

$$fracB = \frac{\beta_{Mg}}{(\alpha_{Mg} + \beta_{Mg})}$$

$$PoMg2 = 3 \cdot fracO \cdot fracB \cdot fracB$$

$$PoMg1 = 3 \cdot fracO \cdot fracO \cdot fracB$$

$$Po = fracO \cdot fracO \cdot fracO$$

Spermine block

$$\alpha_{SPM} = \frac{0.17 \cdot e^{-0.07 \cdot \left((V_m - E_K) + 8.0 \cdot [Mg^{2+}]_i\right)}}{1.0 + 0.01 \cdot e^{0.12 \cdot ((V_m - E_K) + 8.0 \cdot [Mg^{2+}]_i)}}$$

$$\beta_{SPM} = \frac{[SPM]_i \cdot 280.0 \cdot e^{0.15 \cdot \left((V_m - E_K) + 8.0 \cdot [Mg^{2+}]_i\right)}}{1.0 + 0.01 \cdot e^{0.13 \cdot ((V_m - E_K) + 8.0 \cdot [Mg^{2+}]_i)}}$$

$$\frac{dP(O)_{IK1}}{dt} = \alpha_{SPM} \cdot (1 - P(O)_{IK1}) - \beta_{SPM} \cdot P(O)_{IK1} \cdot Po$$

$$phi = 0.9$$

$$P(OpenMode1) = phi \cdot P(O)_{IK1} \cdot \left(Po + \tfrac{2}{3} \cdot PoMg1 + \tfrac{1}{3} \cdot PoMg2\right)$$

$$P(OpenMode2) = \frac{1 - phi}{1.0 + \frac{[SPM]_i \cdot 1000}{40.0 \cdot e^{-\frac{(V_m - E_K)}{9.1}}}}$$

I_{Kr} : delayed rectifier K^+ current, rapid component (pA pF^{-1})

Adopted from Kuzumoto et al. (Kuzumoto *et al.*, 2008).

G_{IKr}	Conductance of I_{Kr}
A_{Kdep}	Extracellular K^+-dependent factor
$I_{Kr}K$	K^+ ion current through I_{Kr}

$$I_{Kr} = G_{IKr} \cdot (V_m - E_K) \cdot (0.6 \cdot P(y_1)_{IKr} + 0.4 \cdot P(y_2)_{IKr}) \cdot P(y_3)_{IKr}$$

$$I_{Kr}K = I_{Kr}$$

$$A_{Kdep} = \left(\frac{[K^+]_o}{5.4}\right)^{0.2}$$

$$G_{IKr} = 0.035 \cdot A_{Kdep}$$

Fast activation:

$$\alpha_{fa} = \frac{1.0}{20.0 \cdot e^{\left(-\frac{V_m}{11.5}\right)} + 5.0 \cdot e^{\left(-\frac{V_m}{300.0}\right)}}$$

$$\beta_{fa} = \frac{1.0}{160.0 \cdot e^{\left(\frac{V_m}{28.0}\right)} + 200 \cdot e^{\left(\frac{V_m}{1000.0}\right)}} + \frac{1.0}{2500.0 \cdot e^{\left(\frac{V_m}{20.0}\right)}}$$

$$\frac{dP(y_1)_{IKr}}{dt} = \alpha_{fa} \cdot (1.0 - P(y_1)_{IKr}) - \beta_{fa} \cdot P(y_1)_{IKr}$$

Slow activation:

$$\alpha_{sa} = \frac{1.0}{200.0 \cdot e^{\left(-\frac{V_m}{13.0}\right)} + 20.0 \cdot e^{\left(-\frac{V_m}{300.0}\right)}}$$

$$\beta_{sa} = \frac{1.0}{1600.0 \cdot e^{\left(\frac{V_m}{28.0}\right)} + 2000.0 \cdot e^{\left(\frac{V_m}{1000.0}\right)}} + \frac{1.0}{10000.0 \cdot e^{\left(\frac{V_m}{20.0}\right)}}$$

$$\frac{dP(y_2)_{IKr}}{dt} = \alpha_{sa} \cdot (1.0 - P(y_2)_{IKr}) - \beta_{sa} \cdot P(y_2)_{IKr}$$

Inactivation:

$$\alpha_{i} = \frac{1.0}{10.0 \cdot e^{\left(\frac{V_m}{17.0}\right)} + 2.5 \cdot e^{\left(\frac{V_m}{300.0}\right)}}$$

$$\beta_{i} = \frac{1.0}{0.35 \cdot e^{\left(-\frac{V_m}{17.0}\right)} + 2.0 \cdot e^{\left(-\frac{V_m}{150.0}\right)}}$$

$$\frac{dP(y_3)_{IKr}}{dt} = \alpha_{i} \cdot (1.0 - P(y_3)_{IKr}) - \beta_{i} \cdot P(y_3)_{IKr}$$

I_{Ks} : delayed rectifier K^+ current, slow component (pA pF^{-1})

Adapted from Kuzumoto et al. (Kuzumoto *et al.*, 2008): PKA activation is omitted.

$I_{Ks}K$	K^+ ion component of I_{Ks}
$I_{Ks}Na$	Na^+ ion component of I_{Ks}
$ISOfactor_{IKs}$	β adrenergic receptor activation factor for I_{Ks}

$I_{Ks} = I_{Ks}K + I_{Ks}Na$

$P_{IKs} = 0.025$ (This value is the one in Kuzumoto et al. (Kuzumoto *et al.*, 2008) without β adrenergic receptor stimulation.)

$ISOfactor_{IKs} = 1.0$

$I_{Ks}K = P_{IKs} \cdot CF_K \cdot {P(y_1)_{IKs}}^2 \cdot (0.9 \cdot P(y_2)_{IKs} + 0.1) \cdot ISOfactor_{IKs}$

$I_{Ks}Na = 0.04 \cdot P_{IKs} \cdot CF_{Na} \cdot {P(y_1)_{IKs}}^2 \cdot (0.9 \cdot P(y_2)_{IKs} + 0.1) \cdot ISOfactor_{IKs}$

Activation:

$$\alpha_{aIKs} = \frac{1.0}{85.0 \cdot e^{\left(-\frac{V_m}{10.5}\right)} + 370.0 \cdot e^{\left(-\frac{V_m}{62.0}\right)}}$$

$$\beta_{aIKs} = \frac{1}{1450.0 \cdot e^{\left(\frac{V_m}{20.0}\right)} + 300.0 \cdot e^{\left(\frac{V_m}{210.0}\right)}}$$

$$\frac{dP(y_1)_{IKs}}{dt} = \alpha_{aIKs} \cdot (1.0 - P(y_1)_{IKs}) - \beta_{aIKs} \cdot P(y_1)_{IKs}$$

Ca^{2+}-dependent gate:

$$\mathrm{y}_2 \underset{\alpha_{y2}}{\overset{\beta_{y2}}{\rightleftarrows}} \mathrm{y}_{C2} \underset{\alpha_{yC2}}{\overset{\beta_{yC2}}{\rightleftarrows}} (1.0 - \mathrm{y}_2 - \mathrm{y}_{C2})$$

$\alpha_{y2} = 4.0 \cdot [Ca^{2+}]_i$

$\beta_{y2} = 0.000148$

$\alpha_{yC2} = 0.005$

$\beta_{yC2} = 0.03$

$$\frac{dP(y_2)_{IKs}}{dt} = \alpha_{y2} \cdot y_{C2} - \beta_{y2} \cdot P(y_2)_{IKs}$$

$$\frac{dP(y_{C2})_{IKs}}{dt} = \alpha_{yC2} \cdot (1.0 - P(y_2)_{IKs} - P(y_{C2})_{IKs}) - \beta_{yC2} \cdot P(y_{C2})_{IKs} + \beta_{y2} \cdot P(y_2)_{IKs} - \alpha_{y2} \cdot P(y_{C2})_{IKs}$$

I_{to} : transient outward current (pA pF^{-1})

Adopted from Kuzumoto et al. (Kuzumoto *et al.*, 2008).

$I_{to}K$	K^+ ion component of I_{to}
$I_{to}Na$	Na^+ ion component of I_{to}

$I_{to} = I_{to}K + I_{to}Na$

$P_{Ito}K = 0.00025$

$I_{to}K = P_{Ito}K \cdot CF_K \cdot {P(y_1)_{Ito}}^3 \cdot P(y_2)_{Ito}$

$I_{to}Na = 0.09 \cdot P_{Ito}K \cdot CF_{Na} \cdot {P(y_1)_{Ito}}^3 \cdot P(y_2)_{Ito}$

Activation:

$$\alpha_1 = \frac{1.0}{11.0 \cdot e^{\left(-\frac{V_m}{28.0}\right)} + 0.2 \cdot e^{\left(-\frac{V_m}{400.0}\right)}}$$

$$\beta_1 = \frac{1.0}{4.4 \cdot e^{\left(\frac{V_m}{16.0}\right)} + 0.2 \cdot e^{\left(\frac{V_m}{500.0}\right)}}$$

$$\frac{dP(y_1)_{Ito}}{dt} = \alpha_1 \cdot (1.0 - P(y_1)_{Ito}) - \beta_1 \cdot P(y_1)_{Ito}$$

Inactivation:

$$\alpha_2 = \frac{0.0038 \cdot e^{\left(-\frac{V_m+13.5}{11.3}\right)}}{1 + 0.051335 \cdot e^{\left(-\frac{V_m+13.5}{11.3}\right)}}, \quad \beta_2 = \frac{0.0038 \cdot e^{\left(\frac{V_m+13.5}{11.3}\right)}}{1 + 0.067083 \cdot e^{\left(\frac{V_m+13.5}{11.3}\right)}}$$

$$\frac{dP(y_2)_{Ito}}{dt} = \alpha_2 \cdot (1 - P(y_2)_{Ito}) - \beta_2 \cdot P(y_2)_{Ito}$$

Membrane currents: Cl⁻ currents

I_{VRCC} : volume regulated Cl⁻ channel current (pA pF^{-1})	
	Omitted from Kuzumoto et al. (Kuzumoto *et al.*, 2008). Cell volume is set constant.
I_{CFTR} : cystic fibrosis transmembrane conductance regulator (CFTR) Cl⁻ channel current (pA pF^{-1})	
	Omitted from Kuzumoto et al. (Kuzumoto *et al.*, 2008).
I_{Clb} : background Cl⁻ current (pA pF^{-1})	
	Adapted from Kuzumoto et al. (Kuzumoto *et al.*, 2008).
	$I_{Clb} = -P_{Clb} \cdot CF_{Cl}$<br>$P_{Clb} = 0.0015625$: (changed to maintain cytosolic Cl⁻ concentration.)

Background currents

I_{NSC} : background non-selective cation current (pA pF^{-1})	
	Adopted from Kuzumoto et al. (Kuzumoto *et al.*, 2008).
	$I_{NSC} = I_{NSC}Na + I_{NSC}K$<br>$I_{NSC}Na = P_{NSC} \cdot CF_{Na}$<br>$I_{NSC}K = 0.4 \cdot P_{NSC} \cdot CF_K$<br>$P_{NSC} = 0.000569$
I_{Kpl} : voltage-dependent K$^+$ current (plateau current) (pA pF^{-1})	
	Adopted from Kuzumoto et al. (Kuzumoto *et al.*, 2008).
	$P_{IKpl} = 8.333 \cdot 10^{-7} \cdot \left(\frac{[K^+]_o}{5.4}\right)^{0.16}$<br>$if\ V_m \neq -3.0$<br>$I_{Kpl}K = P_{IKpl} \cdot CF_K \cdot \frac{V_m + 3.0}{1 - e^{\left(-\frac{V_m+3.0}{13.0}\right)}}$<br>$if\ V_m = -3.0$<br>$I_{Kpl}K = P_{IKpl} \cdot CF_K \cdot 13.0077$<br>$I_{Kpl} = I_{Kpl}K$
$I_{l(Ca)}$: Ca^{2+}-activated background cation current (pA pF^{-1})	
	Adopted from Kuzumoto et al. (Kuzumoto *et al.*, 2008).
	$I_{l(Ca)} = I_{l(Ca)}Na + I_{l(Ca)}K$<br>$I_{l(Ca)}Na = P_{Il(Ca)} \cdot CF_{Na} \cdot P(open)$<br>$I_{l(Ca)}K = P_{Il(Ca)} \cdot CF_K \cdot P(open)$<br>$P_{Il(Ca)} = 0.00675$<br>$P(open) = \frac{1.0}{1.0 + \left(\frac{0.0012}{[Ca^{2+}]_i}\right)^3}$
I_{KATP} : ATP-sensitive K$^+$ current (pA pF^{-1})	
	Adopted from Kuzumoto et al. (Kuzumoto *et al.*, 2008).

	$I_{KATP} = 0.4171 \cdot [K^+]_o{}^{0.24} \cdot (V_m - E_K) \cdot P(Open)$<br>$I_{KATP}K = I_{KATP}$<br>$P(Open) = \frac{0.8}{1.0 + \left(\frac{[ATP]_i}{0.1}\right)^2}$
I_{Cab} : background Ca^{2+} current (pA pF^{-1})	
	Adopted from Kuzumoto et al. (Kuzumoto *et al.*, 2008).
	$I_{Cab} = P_{Cab} \cdot CF_{Ca}$<br>$I_{Cab}Ca = I_{Cab}$<br>$P_{Cab} = 3.03 \cdot 10^{-4}$

Sarcolemmal membrane Transporters

NCX : Na^+/Ca^{2+} exchange		
	Adapted from Kuzumoto et al. (Kuzumoto *et al.*, 2008).	
	I_{NCX}	*NCX* current (pA pF^{-1})
	K_dNa_o	Dissociation constant for Na^+ at the outer side of the membrane. 87.5 mM
	K_dCa_o	Dissociation constant for Ca^{2+} at the outer side of the membrane. 1.38 mM
	K_dNa_i	Dissociation constant for Na^+ at the inner side of the membrane. 20.7 mM
	K_dCa_i	Dissociation constant for Ca^{2+} at the inner side of the membrane. 0.0184 mM
	K_dCa_{act}	Dissociation constant for Ca^{2+} activation at the inner side of the membrane. 0.004 mM
	$I_{NCX} = 110.7955 \cdot \left(k_1 \cdot P(E_1total) - k_2 \cdot P(E_2total)\right) \cdot pHFacor$<br>$I_{NCX}Ca = -2.0 \cdot I_{NCX}$<br>$I_{NCX}Na = 3.0 \cdot I_{NCX}$<br>$P(E_1Na) = \frac{1.0}{1.0 + \left(\frac{K_dNa_i}{[Na^+]_i}\right)^3 \cdot \left(1.0 + \frac{[Ca^{2+}]_i}{K_dCa_i}\right)}, \qquad P(E_2Na) = \frac{1.0}{1.0 + \left(\frac{K_dNa_o}{[Na^+]_o}\right)^3 \cdot \left(1.0 + \frac{[Ca^{2+}]_o}{K_dCa_o}\right)}$<br>$P(E_1Ca) = \frac{1.0}{1.0 + \left(\frac{K_dCa_i}{[Ca^{2+}]_i}\right) \cdot \left(1.0 + \frac{[Na^+]_i}{K_dNa_i}\right)^3}, \qquad P(E_2Ca) = \frac{1.0}{1.0 + \left(\frac{K_dCa_o}{[Ca^{2+}]_o}\right) \cdot \left(1.0 + \frac{[Na^+]_o}{K_dNa_o}\right)^3}$<br>$k_1 = e^{0.32 \cdot \frac{V_m \cdot F}{R \cdot T}} \cdot P(E_1Na), \qquad k_2 = e^{(0.32-1) \cdot \frac{V_m \cdot F}{R \cdot T}} \cdot P(E_2Na), \qquad k_3 = P(E_1Ca), \qquad k_4 = P(E_2Ca)$<br>$f_{Ca} = \frac{[Ca^{2+}]_i}{[Ca^{2+}]_i + K_dCa_{act}}$<br><br>I1 inactivation gate (Na-dependent);<br>$\alpha_1 = P(E_1Na) \cdot (f_{Ca} \cdot 0.002 + (1 - f_{Ca}) \cdot 0.0015), \qquad \beta_1 = f_{Ca} \cdot 0.0012 + (1 - f_{Ca}) \cdot 0.0000005$<br>I2 inactivation gate (Ca-dependent);<br>$\alpha_2 = f_{Ca} \cdot 0.00003 + (1 - f_{Ca}) \cdot 0.01, \qquad \beta_2 = f_{Ca} \cdot 0.09 + (1 - f_{Ca}) \cdot 0.0001$	

$$\frac{dP(E_1 total)}{dt} = P(E_2 total) \cdot (k_2 + k_4) + \beta_1 \cdot P(I_1) + \beta_2 \cdot P(I_2) - P(E_1 total) \cdot (k_1 + k_3 + \alpha_1 + \alpha_2)$$

$$\frac{dP(I_1)}{dt} = \alpha_1 \cdot P(E_1 total) - \beta_1 \cdot P(I_1)$$

$$\frac{dP(I_2)}{dt} = \alpha_2 \cdot P(E_1 total) - \beta_2 \cdot P(I_2)$$

$$P(E_2 total) = 1.0 - \left(P(E_1 total) + P(I_1) + P(I_2)\right)$$

$$pHFactor = \frac{1 + 10^{0.991 \cdot (-7.15 + pK_{NCX})}}{1 + 10^{0.991 \cdot (-pH_i + pK_{NCX})}}, \qquad pK_{NCX} = 7.37$$

NaK : Na^+/K^+ pump

Adapted from Oka et al. (Oka *et al.*, 2010).

I_{NaK}	*NaK* current (pA pF^{-1})
$K_d X$	Dissociation constant for ion X (Na^+_i, Na^+_o, K^+_o, K^+_i)
$K_d^0 X$	Dissociation constant for ion X (Na^+_i, Na^+_o, K^+_o, K^+_i) at Vm = 0
$K_d MgATP_i$	Dissociation constant for MgATP, 0.1 mM. Decreased from 0.6 to 0.1.
ΔX	Coefficient of ion X (Na^+_i, Na^+_o, K^+_o, K^+_i) for voltage dependence
$\alpha_1^+, \alpha_2^+, \alpha_3^+, \alpha_4^+$	Apparent rate constants for forward step
$\alpha_1^-, \alpha_2^-, \alpha_3^-, \alpha_4^-$	Apparent rate constants for backward step
v_{cyc}	Steady state cycle rate
N_{Avog}	Avogadro's number, $6.02 \cdot 10^{23}$ mol^{-1}
ρ	Na^+/K^+ pump density, 1560.0. Model adjusted.
$dATPuse_{NaK}$	ATP consumption by I_{NaK} per ms
$dADP_{NaK}$	ADP consumption by I_{NaK} per ms
dPi_{NaK}	Pi consumption by I_{NaK} per ms

$$K_d Na_o = K_d^0 Na_o \cdot e^{\frac{\Delta Na_o \cdot F \cdot V_m}{R \cdot T}}, \qquad K_d^0 Na_o = 26.8 \text{ mM}, \Delta Na_o = 0.44$$
$$K_d Na_i = K_d^0 Na_i \cdot e^{\frac{\Delta Na_i \cdot F \cdot V_m}{R \cdot T}}, \qquad K_d^0 Na_i = 5.0 \text{ mM}, \Delta Na_i = -0.14$$
$$K_d K_o = K_d^0 K_o \cdot e^{\frac{\Delta K_o \cdot F \cdot V_m}{R \cdot T}}, \qquad K_d^0 K_o = 0.8 \text{ mM}, \Delta K_o = 0.23$$
$$K_d K_i = K_d^0 K_i \cdot e^{\frac{\Delta K_o \cdot F \cdot V_m}{R \cdot T}}, \qquad K_d^0 K_i = 18.8 \text{ mM}, \Delta K_i = -0.14$$

Apparent rate constants;

$$\alpha_1^+ = \frac{k_1^+ \cdot \overline{Na_i}^3}{\left(1.0 + \overline{Na_i}\right)^3 + \left(1.0 + \overline{K_i}\right)^2 - 1.0}, \qquad \alpha_1^- = k_1^- \cdot [MgADP]_i$$

$$\alpha_2^+ = k_2^+, \qquad \alpha_2^- = \frac{k_2^- \cdot \overline{Na_o}^3}{\left(1.0 + \overline{Na_o}\right)^3 + \left(1.0 + \overline{K_o}\right)^2 - 1.0}$$

$$\alpha_3^+ = \frac{k_3^+ \cdot \overline{K_o}^2}{\left(1.0 + \overline{Na_o}\right)^3 + \left(1.0 + \overline{K_o}\right)^2 - 1.0}, \qquad \alpha_3^- = \frac{k_3^- \cdot [Pi_{tot}]_i \cdot [H^+]_i}{1.0 + \overline{MgATP_i}}$$

$$\alpha_4^+ = \frac{k_4^+ \cdot \overline{MgATP_i}}{1.0 + \overline{MgATP_i}}, \qquad \alpha_4^- = \frac{k_4^- \cdot \overline{K_i}^2}{\left(1.0 + \overline{Na_i}\right)^3 + \left(1.0 + \overline{K_i}\right)^2 - 1.0}$$

where

$$\overline{Na_o} = \frac{[Na^+]_o}{K_d Na_o}, \quad \overline{Na_i} = \frac{[Na^+]_i}{K_d Na_i}, \quad \overline{K_o} = \frac{[K^+]_o}{K_d K_o}, \quad \overline{K_i} = \frac{[K^+]_i}{K_d K_i}, \quad \overline{MgATP_i} = \frac{[MgATP]_i}{K_d MgATP_i}$$

$k_1^+ = 1.2528\, ms^{-1}$, $k_1^- = 0.1392\, ms^{-1} mM^{-1}$, $k_2^+ = 0.1392\, ms^{-1}$, $k_2^- = 0.01392\, ms^{-1}$

$k_3^+ = 6.96\, ms^{-1}$, $k_3^- = 13920\, ms^{-1} mM^{-2}$, $k_4^+ = 0.522\, ms^{-1}$, $k_4^- = 0.348\, ms^{-1}$

$$v_{cyc} = \frac{\alpha_1^+ \cdot \alpha_2^+ \cdot \alpha_3^+ \cdot \alpha_4^+ - \alpha_1^- \cdot \alpha_2^- \cdot \alpha_3^- \cdot \alpha_4^-}{\Sigma}$$

where

$$X1 = \alpha_2^- \cdot \alpha_3^- \cdot (\alpha_4^- + \alpha_1^+) + \alpha_4^+ \cdot \alpha_1^+ \cdot (\alpha_2^- + \alpha_3^+)$$

$$X2 = \alpha_2^+ \cdot \alpha_1^+ \cdot (\alpha_3^- + \alpha_4^+) + \alpha_3^- \cdot \alpha_4^- \cdot (\alpha_2^+ + \alpha_1^-)$$

$$X3 = \alpha_2^+ \cdot \alpha_3^+ \cdot (\alpha_4^- + \alpha_1^+) + \alpha_4^- \cdot \alpha_1^- \cdot (\alpha_2^- + \alpha_3^+)$$

$$X4 = \alpha_2^- \cdot \alpha_1^- \cdot (\alpha_3^- + \alpha_4^+) + \alpha_3^+ \cdot \alpha_4^+ \cdot (\alpha_2^+ + \alpha_1^-)$$

$$\Sigma = X1 + X2 + X3 + X4$$

$$I_{NaK} = \frac{\rho \cdot 10^{20} \cdot F}{N_{Avog}} \cdot v_{cyc} \cdot pHFactor$$

$$I_{NaK}Na = 3.0 \cdot I_{NaK}$$

$$I_{NaK}K = -2.0 \cdot I_{NaK}$$

$$dATPuse_{NaK} = coeff \cdot (\alpha_1^+ \cdot P_{1\_6} - \alpha_1^- \cdot P_7)$$

$$dADP_{NaK} = coeff \cdot (-\alpha_4^+ \cdot P_{14\_15} + \alpha_4^- \cdot P_{1\_6})$$

$$dPi_{NaK} = dADP_{NaK}$$

where

$$pHFactor = \frac{1.0 + 10^{0.3457 \cdot (-7.15 + pK1)}}{(1.0 + 10^{0.3457 \cdot (-pHi + pK1)}) \cdot \left(1.0 + 10^{1.5556 \cdot (-(-pHi + pK2))}\right)}$$

$pK1 = 9.2167,\ pK2 = 8.5574$

$$coeff = \frac{\rho \cdot C_m \cdot 10^5}{N_{Avog} \cdot V_i \cdot 10^{-15}}$$

$$P_{1\_6} = \frac{X4}{\Sigma}, \quad P_7 = \frac{X1}{\Sigma}, \quad P_{8\_13} = \frac{X2}{\Sigma}, \quad P_{14\_15} = \frac{X3}{\Sigma}$$

PMCA : Plasma membrane Ca^{2+}-ATPase

Adapted from Kuzumoto et al. (Kuzumoto *et al.*, 2008). The condition of no beta stimulation was used for $K_d Ca_i$ and A_{PMCA}.

$$\begin{array}{ccccc} E_2Ca & \xleftrightarrow{K_d Ca_o} & E_2 & & 1-y \\ k_1 \uparrow\downarrow k_2 & & k_3 \uparrow\downarrow k_4 & & \beta \uparrow\downarrow \alpha \\ E_1Ca & \xleftrightarrow{K_d Ca_i} & E_1 & & y \end{array}$$

	I_{PMCA}	*PMCA* current (pA pF^{-1})
	$K_d X$	Dissociation constant for ion X (Ca^{2+}_o, Ca^{2+}_i)

	$CaMCa$	Ca^{2+}-bound calmodulin
	k_1, k_2, k_3, k_4	Rate constants
	α, β	Rate constants in the reduced model
	A_{PMCA}	Amplitude converting factor of *PMCA*
	$dATP_{PMCA}$	ATP consumption by *PMCA* per ms

$$I_{PMCA} = A_{PMCA} \cdot \left(k_1 \cdot P(E_1Ca) \cdot P(y)_{PMCA} - k_2 \cdot P(E_2Ca) \cdot (1 - P(y)_{PMCA})\right)$$

$$I_{PMCA}Ca = I_{PMCA}$$

$$dATPuse_{PMCA} = \frac{I_{PMCA}}{2.0 \cdot F \cdot V_i}$$

where

$$A_{PMCA} = \frac{0.08}{1.0 + \frac{0.00005}{[CaMCa]}} - 0.00909$$

$$K_dCa_o = 2.0, \qquad K_dCa_i = \frac{180 - 6.4}{100000 \cdot \left(1.0 + \frac{[CaMCa]}{0.00005} + 6.4\right)}$$

$$k_1 = \frac{1.0}{1.0 + \frac{0.1}{[ATP]}}, \qquad k_2 = 0.01, \qquad k_3 = 0.01, \qquad k_4 = 1.0$$

$$P(E_1Ca) = \frac{1.0}{1.0 + \left(\frac{K_dCa_i}{[Ca^{2+}]_i}\right)^3}, \qquad P(E_1) = 1.0 - P(E_1Ca)$$

$$P(E_2Ca) = \frac{1.0}{1.0 + \left(\frac{K_dCa_o}{[Ca^{2+}]_o}\right)^3}, \qquad P(E_2) = 1.0 - P(E_2Ca)$$

In the reduced two-state model,

$$\alpha = k_2 \cdot P(E_2Ca) + k_4 \cdot P(E_2)$$

$$\beta = k_1 \cdot P(E_1Ca) + k_3 \cdot P(E_1)$$

$$\frac{dP(y)_{PMCA}}{dt} = \alpha \cdot (1.0 - P(y)_{PMCA}) - \beta \cdot P(y)_{PMCA}$$

NKCC1 : $Na^+/K^+/2Cl^-$ cotransporter 1

Adapted from Kuzumoto et al. (Kuzumoto *et al.*, 2008). Assuming rapid equilibrium.

$$\begin{array}{ccccccccccc} & E_1 & \xleftrightarrow{K_aNa} & E_1Na & \xleftrightarrow{K_aCl} & E_1NaCl & \xleftrightarrow{K_aK} & E_1NaClK & \xleftrightarrow{K_aCl} & E_1NaClKCl & y \\ k_{fEmpty} \uparrow\downarrow k_{bEmpty} & & & & & & & & & k_{bFull} \uparrow\downarrow k_{fFull} & \beta \uparrow\downarrow \alpha \\ & E_2 & \xleftrightarrow{K_aCl} & E_2Cl & \xleftrightarrow{K_aK} & E_2ClK & \xleftrightarrow{K_aCl} & E_2ClKCl & \xleftrightarrow{K_aNa} & E_2ClKClNa & (1-y) \end{array}$$

	J_{NKCC1}	Ion flux through *NKCC1* (amol ms^{-1})
	K_aX	Dissociation constant for ion X (Na^+, K^+, Cl^-)
	$k_{fFull}, k_{bFull}, k_{fEmpty}$	Rate constants
	α, β	Rate constants in the reduced model

$K_aNa = 0.08445, \qquad K_aK = 1.16 \cdot 10^{-3}, \qquad K_aCl = 0.05735$

$k_{fFull} = 3.065, \qquad k_{bFull} = 1.456, \qquad k_{fEmpty} = 37.767, \qquad k_{bEmpty} = 79.522$

$$P(E_1) = 1 \Big/ \left(\begin{array}{c} 1 + K_aNa \cdot [Na^+]_o \\ +K_aNa \cdot [Na^+]_o \cdot K_aCl \cdot [Cl^-]_o \\ +K_aNa \cdot [Na^+]_o \cdot K_aCl \cdot [Cl^-]_o \cdot K_aK \cdot [K^+]_o \\ +K_aNa \cdot [Na^+]_o \cdot K_aCl \cdot [Cl^-]_o \cdot K_aK \cdot [K^+]_o \cdot K_aCl \cdot [Cl^-]_o \end{array} \right)$$

$$P(E_1NaClKCl) = K_aNa \cdot [Na^+]_o \cdot K_aCl \cdot [Cl^-]_o \cdot K_aK \cdot [K^+]_o \cdot K_aCl \cdot [Cl^-]_o \cdot P(E_1)$$

$$P(E_2) = 1 \Big/ \left(\begin{array}{c} 1 + K_aCl \cdot [Cl^-]_i \\ +K_aK \cdot [K^+]_i \cdot K_aCl \cdot [Cl^-]_i \\ +K_aCl \cdot [Cl^-]_i \cdot K_aK \cdot [K^+]_i \cdot K_aCl \cdot [Cl^-]_i \\ +K_aNa \cdot [Na^+]_i \cdot K_aCl \cdot [Cl^-]_i \cdot K_aK \cdot [K^+]_i \cdot K_aCl \cdot [Cl^-]_i \end{array} \right)$$

$$P(E_2NaClKCl) = K_aNa \cdot [Na^+]_i \cdot K_aCl \cdot [Cl^-]_i \cdot K_aK \cdot [K^+]_i \cdot K_aCl \cdot [Cl^-]_i \cdot P(E_2)$$

In the reduced two-state model,

$$\alpha = k_{fFull} \cdot P(E_1NaClKCl) + k_{bEmpty} \cdot P(E_1)$$

$$\beta = k_{bFull} \cdot P(E_2NaClKCl) + k_{fEmpty} \cdot P(E_2)$$

Assuming rapid equilibrium,

$$y = \frac{\beta}{\alpha + \beta}$$

$$J_{NKCC1} = 0.0359 \cdot \left(k_{fFull} \cdot P(E_1NaClKCl) \cdot y - k_{bFull} \cdot P(E_2NaClKCl) \cdot (1 - y) \right)$$

AE : anion exchanger (Cl^--HCO_3^- exchange, AE) (amol ms^{-1})

Adapted from Crampin and Smith (Crampin & Smith, 2006).

J_{AE}	Ion flux of anion exchanger. (amol ms^{-1})
k_1^+AE	Rate constant, 0.625 ms^{-1}. Model adjusted.
k_1^-AE	Rate constant, 0.347 ms^{-1}
k_2^+AE	Rate constant, 0.354 ms^{-1}
k_2^-AE	Rate constant, 0.360 ms^{-1}
$K_{Cl}AE$	Dissociation constant for Cl^-, 984.0 mM
$K_{HCO_3}AE$	Dissociation constant for HCO_3^-, 111.0 mM
pK_iAE	Cytosolic acidity constant, 7.573
pK_oAE	Extracellular acidity constant, 6.506
V_i	Cell volume accessible for ion diffusion (μm^3). See "Cell volume" section.

$$\beta_1^+AE = \frac{k_1^+AE \cdot K_{Cl}AE \cdot [HCO_3^-]_i}{K_{HCO_3}AE \cdot K_{Cl}AE + K_{Cl}AE \cdot [HCO_3^-]_i + K_{HCO_3}AE \cdot [Cl^-]_i}$$

$$\beta_1^-AE = \frac{k_1^-AE \cdot K_{Cl}AE \cdot [HCO_3^-]_o}{K_{HCO_3}AE \cdot K_{Cl}AE + K_{Cl}AE \cdot [HCO_3^-]_o + K_{HCO_3}AE \cdot [Cl^-]_o}$$

$$\beta_2^+AE = \frac{k_2^+AE \cdot K_{HCO_3}AE \cdot [Cl^-]_o}{K_{HCO_3}AE \cdot K_{Cl}AE + K_{Cl}AE \cdot [HCO_3^-]_o + K_{HCO_3}AE \cdot [Cl^-]_o}$$

$$\beta_2^-AE = \frac{k_2^-AE \cdot K_{HCO_3}AE \cdot [Cl^-]_i}{K_{HCO_3}AE \cdot K_{Cl}AE + K_{Cl}AE \cdot [HCO_3^-]_i + K_{HCO_3}AE \cdot [Cl^-]_i}$$

	$J_{AE} = \frac{\beta_1^+ AE \cdot \beta_2^+ AE - \beta_1^- AE \cdot \beta_2^- AE}{\beta_1^+ AE + \beta_1^- AE + \beta_2^+ AE + \beta_2^- AE} \cdot \frac{1}{\left(1 + \left(\frac{[H^+]_i}{10^{3-pK_i AE}}\right)^{5.11}\right) \cdot \left(1 + \left(\frac{10^{3-pK_o AE}}{[H^+]_o}\right)^{1.44}\right)} \cdot V_i$	
NBC : Na^+-HCO_3^- cotransporter (amol ms^{-1})		
	Adopted from Crampin and Smith (Crampin & Smith, 2006).	
	J_{NBC}	Ion flux of *NBC* (amol ms^{-1})
	$k_1^+ NBC$	Rate constant, 0.00997 ms^{-1}
	$k_1^- NBC$	Rate constant, 0.056 ms^{-1}
	$k_2^+ NBC$	Rate constant, $8.53 \cdot 10^{-5}$ ms^{-1}
	$k_2^- NBC$	Rate constant, $1.42 \cdot 10^{-5}$ ms^{-1}
	$K_{Na} NBC$	Dissociation constant for Na^+, 4870.0 mM
	$K_{HCO_3} NBC$	Dissociation constant for HCO_3^-, 0.00802 mM
	$pK_i NBC$	Cytosolic acidity constant, 6.738
	$pK_o NBC$	Extracellular acidity constant, 7.185
	V_i	Cell volume accessible for ion diffusion (μm^3). See "Cell volume" section.
	$\alpha_1^+ NBC = \frac{k_1^+ NBC \cdot K_{Na} NBC \cdot K_{HCO_3} NBC}{K_{Na} NBC \cdot K_{HCO_3} NBC + K_{HCO_3} NBC \cdot [Na^+]_i + [Na^+]_i \cdot [HCO_3^-]_i}$<br>$\alpha_1^- NBC = \frac{k_1^- NBC \cdot K_{Na} NBC \cdot K_{HCO_3} NBC}{K_{Na} NBC \cdot K_{HCO_3} NBC + K_{HCO_3} NBC \cdot [Na^+]_o + [Na^+]_o \cdot [HCO_3^-]_o}$<br>$\alpha_2^+ NBC = \frac{k_2^+ NBC \cdot [Na^+]_o \cdot [HCO_3^-]_o}{K_{Na} NBC \cdot K_{HCO_3} NBC + K_{HCO_3} NBC \cdot [Na^+]_o + [Na^+]_o \cdot [HCO_3^-]_o}$<br>$\alpha_2^- NBC = \frac{k_2^- NBC \cdot [Na^+]_i \cdot [HCO_3^-]_i}{K_{Na} NBC \cdot K_{HCO_3} NBC + K_{HCO_3} NBC \cdot [Na^+]_i + [Na^+]_i \cdot [HCO_3^-]_i}$<br>$J_{NBC} = \frac{\alpha_1^+ NBC \cdot \alpha_2^+ NBC - \alpha_1^- NBC \cdot \alpha_2^- NBC}{\alpha_1^+ NBC + \alpha_1^- NBC + \alpha_2^+ NBC + \alpha_2^- NBC} \cdot \frac{1}{\left(1 + \left(\frac{10^{3-pK_i NBC}}{[H^+]_i}\right)^{2.91}\right) \cdot \left(1 + \left(\frac{[H^+]_o}{10^{3-pK_o NBC}}\right)^{2.18}\right)} \cdot V_i$	
CHE : Cl^--OH^- exchanger		
	Adopted from Crampin and Smith (Crampin & Smith, 2006).	
	J_{CHE}	Ion flux of *CHE* (amol ms^{-1})
	$k_1^+ CHE$	Rate constant, 0.00429 ms^{-1}
	$k_1^- CHE$	Rate constant, 0.25 ms^{-1}
	$k_2^+ CHE$	Rate constant, 0.0681 ms^{-1}
	$k_2^- CHE$	Rate constant, 0.00117 ms^{-1}
	$K_{Cl} CHE$	Dissociation constant for Cl^-, 18000.0 mM
	$K_{OH} CHE$	Dissociation constant for OH^-, $8.91251 \cdot 10^{-4}$ mM
	V_i	Cell volume accessible for ion diffusion (μm^3). See "Cell volume" section.

	$[OH^-]_i = \frac{10^{-8}}{[H^+]_i}, \quad [OH^-]_o = \frac{10^{-8}}{[H^+]_o}$<br>$\beta_1^+ CHE = \frac{k_1^+ CHE \cdot K_{Cl}CHE \cdot [OH^-]_i}{K_{OH}CHE \cdot K_{Cl}CHE + K_{Cl}CHE \cdot [OH^-]_i + K_{OH}CHE \cdot [Cl^-]_i}$<br>$\beta_1^- CHE = \frac{k_1^- CHE \cdot K_{Cl}CHE \cdot [OH^-]_o}{K_{OH}CHE \cdot K_{Cl}CHE + K_{Cl}CHE \cdot [OH^-]_o + K_{OH}CHE \cdot [Cl^-]_o}$<br>$\beta_2^+ CHE = \frac{k_2^+ CHE \cdot K_{OH^-}CHE \cdot [Cl^-]_o}{K_{OH}CHE \cdot K_{Cl}CHE + K_{Cl}CHE \cdot [OH^-]_o + K_{OH}CHE \cdot [Cl^-]_o}$<br>$\beta_2^- CHE = \frac{k_2^- CHE \cdot K_{OH^-}CHE \cdot [Cl^-]_i}{K_{OH}CHE \cdot K_{Cl}CHE + K_{Cl}CHE \cdot [OH^-]_i + K_{OH}CHE \cdot [Cl^-]_i}$<br>$J_{CHE} = \frac{\beta_1^+ CHE \cdot \beta_2^+ CHE - \beta_1^- CHE \cdot \beta_2^- CHE}{\beta_1^+ CHE + \beta_1^- CHE + \beta_2^+ CHE + \beta_2^- CHE} \cdot V_i$	
NHE : Na^+-H^+ exchanger		
	Adopted from Crampin and Smith (Crampin & Smith, 2006).	
	J_{NHE}	Ion flux of *NHE* (amol ms^{-1})
	$k_1^+ NHE$	Rate constant, 0.0121 ms^{-1}
	$k_1^- NHE$	Rate constant, 0.00329 ms^{-1}
	$k_2^+ NHE$	Rate constant, $7.33 \cdot 10^{-4}$ ms^{-1}
	$k_2^- NHE$	Rate constant, 0.00269 ms^{-1}
	$K_{Na}NHE$	Dissociation constant for Na^+, 33.6 mM
	$K_H NHE$	Dissociation constant for H^+, $1.65 \cdot 10^{-4}$ mM
	$pK_i NHE$	Cytosolic acidity constant, 6.464
	V_i	Cell volume accessible for ion diffusion (μm^3). See "Cell volume" section.
	$\beta_1^+ NHE = \frac{k_1^+ NHE \cdot K_{Na}NHE \cdot [H^+]_i}{K_H NHE \cdot K_{Na}NHE + K_{Na}NHE \cdot [H^+]_i + K_H NHE \cdot [Na^+]_i}$<br>$\beta_1^- CHE = \frac{k_1^- NHE \cdot K_{Na}NHE \cdot [H^+]_o}{K_H NHE \cdot K_{Na}NHE + K_{Na}NHE \cdot [H^+]_o + K_H NHE \cdot [Na^+]_o}$<br>$\beta_2^+ CHE = \frac{k_2^+ CHE \cdot K_H NHE \cdot [Na^+]_o}{K_H NHE \cdot K_{Na}NHE + K_{Na}NHE \cdot [H^+]_o + K_H NHE \cdot [Na^+]_o}$<br>$\beta_2^- CHE = \frac{k_2^- NHE \cdot K_H NHE \cdot [Na^+]_i}{K_H NHE \cdot K_{Na}NHE + K_{Na}NHE \cdot [H^+]_i + K_H NHE \cdot [Na^+]_i}$<br>$J_{NHE} = \frac{\beta_1^+ NHE \cdot \beta_2^+ NHE - \beta_1^- NHE \cdot \beta_2^- NHE}{\beta_1^+ NHE + \beta_1^- NHE + \beta_2^+ NHE + \beta_2^- NHE} \cdot \frac{1}{1 + \left(\frac{10^{3-pK_i NHE}}{[H^+]_i}\right)^{3.18}} \cdot V_i$	

Sarcoplasmic reticulum

SERCA : sarcoplasmic reticulum Ca^{2+} pump		
	Adapted from Kuzumoto et al. (Kuzumoto *et al.*, 2008). pH dependence was added.	
	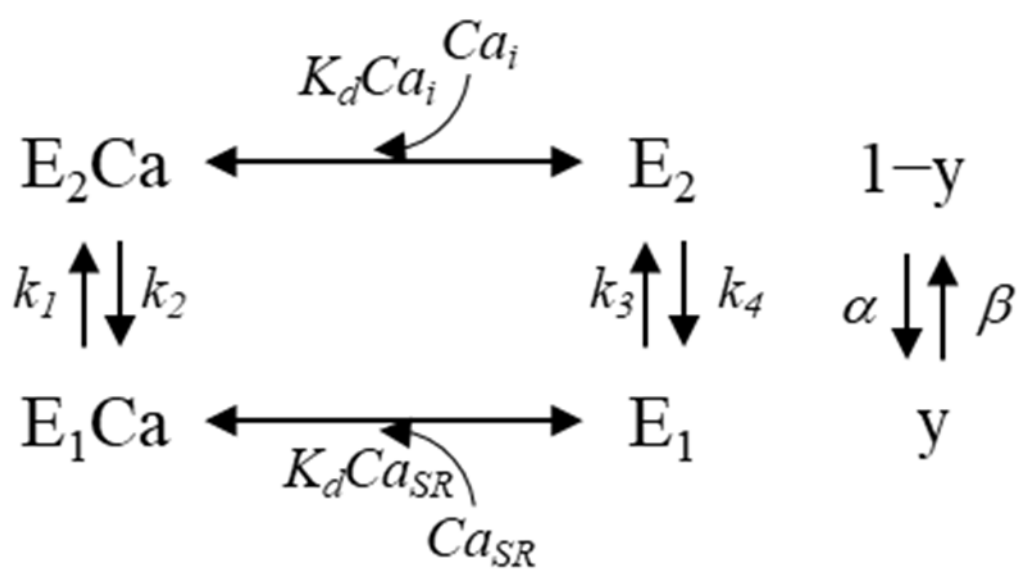	
	I_{SERCA}	Current through *SERCA* (pA pF^{-1})
	$[Ca^{2+}]_{nSR}$	Free Ca^{2+} concentration in network SR
	K_dX	Dissociation constant for ion X ($Ca^{2+}{}_{nSR}$, $Ca^{2+}{}_i$)
	pK_{SERCA}	pK of *SERCA*, 7.53

$K_dCa_{SR} = 3, \qquad K_dCa_i = 6.5387 \cdot 10^{-4}$

$$k_1 = 0.01, \qquad k_2 = \frac{1.0}{1 + \frac{0.1}{[ATP]}}, \qquad k_3 = 1.0, \qquad k_4 = 0.01$$

$$P(E_1Ca) = \frac{1}{1 + \left(\frac{K_dCa_{SR}}{[Ca^{2+}]_{nSR}}\right)^2}, \qquad P(E_1) = 1 - P(E_1Ca)$$

$$P(E_2Ca) = \frac{1}{1 + \left(\frac{K_dCa_i}{[Ca^{2+}]_i}\right)^2}, \qquad P(E_2) = 1 - P(E_2Ca)$$

In the reduced two-state model,

$$\alpha = k_2 \cdot P(E_2Ca) + k_4 \cdot P(E_2)$$

$$\beta = k_1 \cdot P(E_1Ca) + k_3 \cdot P(E_1)$$

$$\frac{dP(y)_{SERCA}}{dt} = \alpha \cdot (1 - P(y)_{SERCA}) - \beta \cdot P(y)_{SERCA}$$

$$pHFactor = \frac{1 + 10^{1.14 \cdot (-7.14 + pK_{SERCA})}}{1 + 10^{1.14 \cdot (-pH_i + pK_{SERCA})}}$$

$$I_{SERCA} = -19.0 \cdot \left(k_1 \cdot P(E_1Ca) \cdot P(y)_{SERCA} - k_2 \cdot P(E_2Ca) \cdot (1 - P(y)_{SERCA})\right) \cdot pHFacor$$

RyR : ryanodine receptor

Adapted from Kuzumoto et al. (Kuzumoto *et al.*, 2008). pH dependence was added.

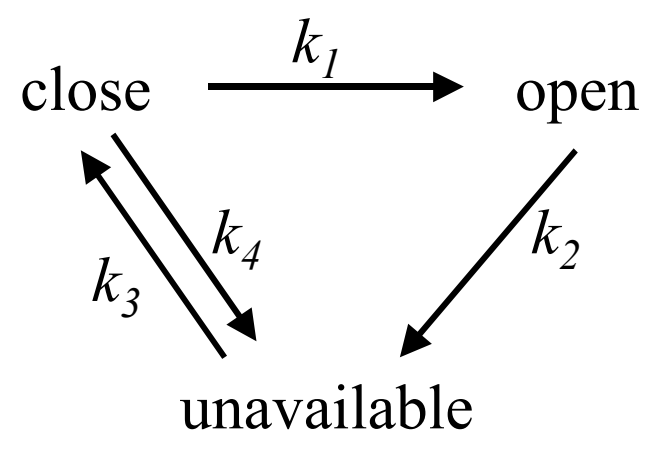

I_{RyR}	Current through *RyR* (pA pF^{-1})
k_1, k_2, k_3, k_4	Rate constants
pK_{RyR}	pK of *RyR*, 6.64
$[Ca^{2+}_{total}]_{jSR}$	Total Ca^{2+} concentration in junctional SR
$[Ca^{2+}]_{jSR}$	Free Ca^{2+} concentration in junctional SR

$$CICRfacor = -150 \cdot 0.0676 \cdot CF_{Ca} \cdot P(OpenCaL)$$

$$k_1 = \frac{0.15}{1 + \left(\frac{0.001}{[Ca^{2+}]_i}\right)^{3.15}} + \frac{CICRfacor}{1 + exp\left(\frac{(0.15 - CICRfacor)}{0.01}\right)}, \qquad k_2 = \frac{0.008}{1 + \frac{0.36}{[Ca^{2+}]_{jSR}}}$$

$$k_3 = 0.000377 \cdot [Ca^{2+}]_{jSR}{}^2, \qquad k_4 = 0.000849$$

$$\frac{dP(close)_{RyR}}{dt} = k_3 \cdot \left(1 - P(close)_{RyR} - P(open)_{RyR}\right) - (k_1 + k_4) \cdot P(close)_{RyR}$$

$$\frac{dP(open)_{RyR}}{dt} = k_1 \cdot P(close)_{RyR} - k_2 \cdot P(open)_{RyR}$$

$$pHFactor = \frac{1 + 10^{1.87\cdot(-7.15 + pK_{RyR})}}{1 + 10^{1.87\cdot(-pH_i + pK_{RyR})}}$$

$$I_{RyR} = 200.0 \cdot P(open) \cdot \left([Ca^{2+}]_{jSR} - [Ca^{2+}]_i\right) \cdot pHFacor$$

Ca^{2+} in sarcoplasmic reticulum

Adapted from Kuzumoto et al. (Kuzumoto *et al.*, 2008).

T_r	Ca^{2+} transfer from network SR to junctional SR (pA pF^{-1}). Conductance was increased tenfold to decrease Ca^{2+} in network SR.
$Caleak_{SR}$	Ca^{2+} leak from SR to cytosol (pA pF^{-1})
$[Ca^{2+}_{tot}]_{jSR}$	Total Ca^{2+} concentration in junctional SR (mM)
$[Ca^{2+}]_{jSR}$	Free Ca^{2+} concentration in junctional SR (mM)

$$T_r = 5.0 \cdot \left([Ca^{2+}]_{nSR} - [Ca^{2+}]_{jSR}\right)$$

$$Caleak_{SR} = 0.3 \cdot ([Ca^{2+}]_{nSR} - [Ca^{2+}]_i)$$

$$\frac{d[Ca^{2+}_{tot}]_{jSR}}{dt} = \frac{\left(T_r - I_{RyR}\right)}{2 \cdot V_{jSR} \cdot F} \cdot C_m, \qquad \frac{d[Ca^{2+}]_{nSR}}{dt} = \frac{(I_{SERCA} - T_r - Caleak_{SR})}{2 \cdot V_{nSR} \cdot F} \cdot C_m$$

$$[Ca^{2+}]_{jSR} = \frac{\left(-b + \sqrt{b^2 + 4 \cdot c}\right)}{2}$$

	$where\ b = B_{max}CSQN + K_dCSQN - [Ca^{2+}_{tot}]_{jSR},\quad c = K_dCSQN \cdot [Ca^{2+}_{tot}]_{jSR}$

Contraction

	Adapted from Kuzumoto et al. (Kuzumoto *et al.*, 2008) and Shim et al. (Shim *et al.*, 2007). pH dependence was added.	
	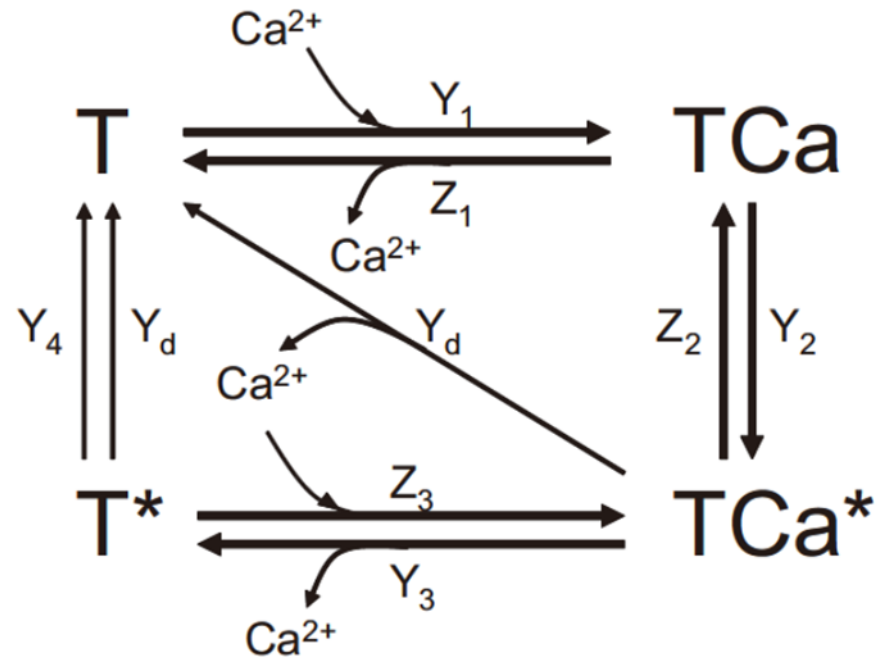	
	$Y_1, Z_1, Y_2, Z_2, Y_3, Z_3, Y_4, Y_d$	Rate constants, in mM^{-1} ms^{-1}, ms^{-1}, ms^{-1}, ms^{-1}, ms^{-1}, mM^{-1} ms^{-1}, ms^{-1}, ms^{-1}
	r_1, r_2, r_3	Adjustment factors; 0.8, 2.0 and 3.0, respectively
	$hsmX$	Length composed of half of the thick filament and the free portion of the thin filament, μm);
	$hsmL$	Half sarcomere length (μm)
	h_c	Equilibrium length of the cross bridge, 0.005 μm
	B	Rate Constant of $dhsmX/dt$, 1.2 μm ms^{-1}
	F_b	Cross-bridge force (mN mm^{-2})
	A_{Fb}	Coefficient of Fb, $1.8 \cdot 10^6$ mN mm^{-2} μm^{-1} mM^{-1}
	F_p	Force of parallel elastic component (mN mm^{-2}); F_p was calculated according to Shim et al. (Shim *et al.*, 2007).
	K_{PE}	Rate Constant of F_p , 3.0 mN mm^{-2}
	D	Coefficient of F_p, 10.0
	L_0	Constant, 0.97 μm
	K_dPI_i	Inhibitory constant of PI_i, 1.83 mM
	K_mATP	Association constant of ATP_i (mM)
	$TrnCaFlux$	Troponin Ca^{2+} releasing,
	$vATP_{CNTR}$	ATP consumption rate by contraction
	$ExternalLoad$	External load, 5.0
	$Y_1 = 39 \cdot r_1, \quad Z_1 = 0.03 \cdot r_2$<br>$Y_2 = 0.0013 \cdot r_3, \quad Z_2 = 0.013 \cdot r_3$<br>$Y_3 = 0.03 \cdot r_2, \quad Z_3 = 1560 \cdot r_1$<br>$Y_4 = 0.04 \cdot r_3, \quad Y_d = 8000$	

Mechanics

$$\frac{dhsmX}{dt} = B \cdot (hsmL - hsmX - h_c)$$

$$F_b = A_{Fb} \cdot ([TCa^*] + [T^*]) \cdot (hsmL - hsmX)$$

$$F_p = \begin{cases} K_{PE} \cdot (e^{D \cdot \left(\frac{hsmL}{L_0} - 1\right)} - 1), & hsmX > L_0 \\ K_{PE} \cdot D \cdot \left(\frac{hsmL}{L_0} - 1\right), & hsmX \le L_0 \end{cases}$$

Troponin

$$pHFactor1 = \frac{1 + \left(\frac{10^{(3-7.15)}}{10^{(3-6.79)}}\right)^{1.65}}{1 + \left(\frac{[H^+]_i}{10^{(3-6.79)}}\right)^{1.65}}$$

$$pHFactor2 = \frac{1 + \left(\frac{10^{(3-7.15)}}{41.6 \cdot 10^{-6}}\right)^{1.47}}{1 + \left(\frac{[H^+]_i}{41.6 \cdot 10^{-6}}\right)^{1.47}} \cdot (1 - 0.414) + 0.414$$

$$PiFactor = 0.79 \cdot \frac{K_d PI_i}{K_d PI_i + [Pi_{tot}]_i} + 0.21$$

$$ATPFactor = \frac{[ATPMg]_i^3}{[ATPMg]_i^3 + K_m ATP^3}$$

$$K_m ATP = 0.464 + 10 \cdot \frac{0.1^2}{[PCr]_i^2 + 0.1^2}$$

$$Y_1 eff = Y_1 \cdot pHFactor1 \cdot [Ca^{2+}]_i, \qquad Z_1 eff = Z_1$$

$$Y_2 eff = Y_2 \cdot PIFactor \cdot pHFactor2 \cdot e^{\left(-20 \cdot (hsmL - 1.17)^2\right)}, \qquad Z_2 eff = Z_2 \cdot ATPFactor$$

$$Y_4 eff = Y_4 \cdot ATPFactor, \qquad Z_3 eff = Z_3 \cdot pHFactor1 \cdot [Ca^{2+}]_i$$

$$Y_d eff = \begin{cases} Y_d \cdot \frac{\left(\frac{dhsmX}{dt}\right)^2}{50}, & \frac{dhsmX}{dt} > 0 \\ Y_d \cdot \left(\frac{dhsmX}{dt}\right)^2, & \frac{dhsmX}{dt} \le 0 \end{cases}$$

$$[T] = TrnTotal - ([TCa] + [TCa^*] + [T^*])$$

$$\frac{d[TCa]}{dt} = Y_1 eff \cdot [T] + Z_2 eff \cdot [TCa^*] - (Z_1 eff + Y_2 eff) \cdot [TCa]$$

$$\frac{d[TCa^*]}{dt} = Y_2 eff \cdot [TCa] + Z_3 eff \cdot [T^*] - (Z_2 eff + Y_3 + Y_d eff) \cdot [TCa^*]$$

$$\frac{d[T^*]}{dt} = Y_3 \cdot [TCa^*] - (Z_3 eff + Y_4 eff + Y_d eff) \cdot [T^*]$$

$$TrnCaFlux = Z_1 eff \cdot [TCa] - Y_1 eff \cdot [T] + Y_3 \cdot [TCa^*] - Z_3 eff \cdot [T^*] + Y_d eff \cdot [TCa^*]$$

$$dATPuse_{CNTR} = 20 \cdot (Z_2 eff \cdot [TCa^*] + Y_4 eff \cdot [T^*])$$

	$\frac{dhsmL}{dt} = \frac{ExternalLoad - (F_b + F_p)}{10.0}$

Mitochondria

	Adapted from Kuzumoto et al. (Kuzumoto *et al.*, 2008), and modified according to Dzbek and Korzeniewski (Dzbek & Korzeniewski, 2008) and Saito et al. (Saito *et al.*, 2016).	
	E_mNAD	NAD redox potential
	E_mUQ	Ubiquinone redox potential
	E_mCytc	Cytochrome c redox potential
	E_mCyta	Cytochrome a redox potential
	E_mNAD_0	NAD standard redox potential, −320 mV
	E_mUQ_0	Ubiquinone standard redox potential, 40 mV
	$[\mathrm{NAD}_{tot}]$	Total NAD concentration, 2.97 mM
	[NADH]	NADH concentration (mM)
	[NAD]	NAD concentration (mM)
	$[UQ_{tot}]$	Total ubiquinone concentration, 0.135 mM
	$[UQH_2]$	Ubiquinol concentration (mM)
	$[UQ]$	Ubiquinone concentration (mM)
	$[Cytc_r]$	Reduced cytochrome c concentration (mM)
	E_mCytc_0	Cytochrome c standard redox potential, 250 mV
	E_mCyta_0	Cytochrome a standard redox potential, 540 mV
	$[Cyta_{tot}]$	Total cytochrome a concentration, 0.135 mM
	$[Cyta_r]$	Reduced cytochrome a concentration (mM)
	$[Cyta_o]$	Oxidized cytochrome a concentration (mM)
	$[Adenine_{mit,tot}]$	Total concentration of adenine nucleotides in mitochondria, 16.26 mM
	$[ADP_{tot}]_{mit}$	Total concentration of ADP in mitochondria (mM)
	$[ADP_{free}]_{mit}$	Concentration of free ADP in mitochondria (mM)
	$[ATP_{tot}]_{mit}$	Total concentration of ATP in mitochondria (mM)
	$[ATP_{free}]_{mit}$	Concentration of free ATP in mitochondria (mM)
	K_dATPMg_{mit}	Dissociation constant for ATP in mitochondria for Mg^{2+}, $17.0 \cdot 10^{-3}$ mM
	K_dADPMg_{mit}	Dissociation constant for ADP in mitochondria for Mg^{2+}, $282.0 \cdot 10^{-3}$ mM
	$[Mg^{2+}]_{mit}$	Concentration of free Mg^{2+} in mitochondria, 0.38 mM
	$Rbuf_{mit}$	H^+ buffering capacity coefficient in mitochondria
	n_{ATP}	ATP synthase proton stoichiometry, 2.5
	$[Pi_{tot}]_{mit}$	Total concentration of inorganic phosphate in mitochondria (mM)

	$[Pi_{tot}]_i$	Total concentration of inorganic phosphate in cytosol (mM)
	C_{mit}	Inner membrane electrical capacitance, 0.001 mM mV^{-1}
	$[Ca^{2+}_{tot}]_{mit}$	Total concentration of Ca^{2+} in mitochondria (mM)
	$[Ca^{2+}]_{mit}$	Free concentration of Ca^{2+} in mitochondria (mM)
	B_{total}	Mitochondrial total Ca^{2+} buffering capacity, 0.6 mM
	K_dCa_{mit}	Dissociation constant of mitochondrial Ca^{2+} buffer for Ca^{2+}, 0.001 mM
	$\Delta\Psi$	Mitochondrial membrane potential (mV)
	ΔpH	Difference between cytosolic and mitochondrial pH
	ΔP	Protonmotive force (mV)
	SF_{mit}	Scaling factor; 1.0 for non-ischemia and ischemia, and 0.5 for reperfusion
Calculations		
	$\Delta pH = \ln(10) \cdot \frac{R \cdot T}{F} \cdot (pH_{mit} - pH_i)$<br>$\Delta P = \Delta pH - \Delta\Psi$<br>$E_mNAD = E_mNAD_0 + \frac{R \cdot T}{2 \cdot F} \cdot \ln\left(\frac{[NAD]}{[NADH]}\right)$<br>$E_mUQ = E_mUQ_0 + \frac{R \cdot T}{2 \cdot F} \cdot \ln\left(\frac{[UQ]}{[UQH_2]}\right)$<br>$E_mCytc = E_mCytc_0 + \frac{R \cdot T}{F} \cdot \ln\left(\frac{[Cytc_0]}{[Cytc_r]}\right)$<br>$E_mCyta = E_mCytc + \Delta P - \Delta\Psi$<br>$[NAD] = [NAD_{tot}] - [\mathrm{NADH}]$<br>$[UQ] = [UQ_{tot}] - [\mathrm{UQH_2}]$<br>$[Cyta_r] = \frac{[Cyta_{tot}]}{1 + e^{\frac{F}{R \cdot T}(E_mCyta - E_mCyta_0)}}$<br>$[Cyta_o] = [Cyta_{tot}] - [Cyta_r]$<br>$[ADP_{tot}]_{mit} = [Adenine_{tot}]_{mit} - [ATP_{tot}]_{mit}$<br>$[ATP_{free}]_{mit} = \frac{[ATP_{tot}]_{mit}}{1 + \left(\frac{[Mg^{2+}]_{mit}}{K_dATPMg_{mit}}\right)}$<br>$[ADP_{free}]_{mit} = \frac{[ADP_{tot}]_{mit}}{1 + \left(\frac{[Mg^{2+}]_{mit}}{K_dADPMg_{mit}}\right)}$<br>$Rbuf_{mit} = \frac{0.022}{\frac{10^{-pH_{mit}} - 10^{-pH_{mit} - 0.001}}{0.001}}$	
Substrate dehydrogenation (DH)		
	v_{DH}	Rate of substrate dehydrogenation (mM ms^{-1})
	k_{DH}	Rate constant of substrate dehydrogenation, $3.12 \cdot 10^{-4}$ mM ms^{-1}. Model adjusted

	K_{mDH}	Association constant of substrate dehydrogenation, 1.0. Model adjusted
	$v_{DH} = SF_{mit} \cdot k_{DH} \cdot \frac{1}{1 + \left(\frac{K_{mDH}}{\frac{[NAD]}{[NADH]}} \right)^{0.8}}$	
Complex I (C1)		
	v_{C1}	Rate of complex I (mM ms^{-1})
	k_{C1}	Rate constant of complex I, $3.27844 \cdot 10^{-5}$ mM ms^{-1} mV^{-1}. Model adjusted.
	ΔG_{C1}	Thermodynamic span of complex I (mV)
	$v_{C1} = SF_{mit} \cdot k_{C1} \cdot \Delta G_{C1}$<br>$\Delta G_{C1} = E_m UQ - E_m NAD - 2 \cdot \Delta P$	
Complex III (C3)		
	v_{C3}	Rate of complex III (mM ms^{-1})
	k_{C3}	Rate constant of complex III, 1.8716×10^{-5} mM ms^{-1} mV^{-1}. Model adjusted.
	ΔG_{C3}	Thermodynamic span of complex III (mV)
	$v_{C3} = SF_{mit} \cdot k_{C3} \cdot \Delta G_{C3}$<br>$\Delta G_{C3} = E_m Cytc - E_m UQ - 2 \cdot \Delta P - \Delta \Psi$	
Complex IV (C4)		
	v_{C4}	Rate of complex IV (mM ms^{-1})
	k_{C4}	Rate constant of complex IV, 0.98784 mM ms^{-1}. Model adjusted
	$K_m C1O_2$	Association constant of C4 for oxygen, 0.150 mM
	$v_{C4} = SF_{mit} \cdot k_{C4} \cdot [Cytc_r] \cdot [Cyta_r] \cdot \frac{1}{1 + \frac{K_m C1O_2}{[O_2]_i}}$	
Proton leak (HLeak)		
	v_{HLeak}	Rate of proton leak (mM ms^{-1})
	k_{HLeak}	Rate constant of proton leak, 4.00204×10^{-7} mM ms^{-1}. Model adjusted.
	$v_{HLeak} = SF_{mit} \cdot k_{HLeak} \cdot (e^{0.038 \cdot dP} - 1)$	
ATP synthase (SN)		
	v_{SN}	Rate of ATP synthase (mM ms^{-1})
	k_{SN}	Rate constant of ATP synthase, $4.70824 \cdot 10^{-3}$ mM ms^{-1}. Model adjusted.
	ΔG_{SN0}	Standard thermodynamic span of ATP synthase, 31.9 kJ mol^{-1}
	$v_{SN} = SF_{mit} \cdot k_{SN} \cdot \frac{\gamma_{SN} - 1}{\gamma_{SN} + 1}$<br>$\gamma_{SN} = e^{\left(n_{ATP} \cdot \frac{F \cdot \Delta p}{R \cdot T} - \left(\frac{\Delta G_{SN0} \cdot 1000}{R \cdot T} + \ln\left(\frac{[ATP_{tot}]_{mit} \cdot 1000}{[ADP_{tot}]_{mit} \cdot [PI_{tot}]_{mit}} \right) \right) \right)}$	

Adenine nucleotides antiport (ANT)		
	v_{ANT}	Rate of Adenine nucleotides antiport (mM ms^{-1})
	k_{ANT}	Rate constant of Adenine nucleotides antiport, 7.4874×10^{-3} mM ms^{-1}. Model adjusted.
	$K_mANTADP$	Association constant of ANT for ADP, 0.0035 mM
	$v_{ANT} = SF_{mit} \cdot k_{ANT} \cdot \frac{1}{1+\frac{K_mANTADP}{[ADP_{free}]_i}} \cdot \left(\frac{[ADP_{free}]_i}{[ADP_{free}]_i + [ATP_{free}]_i \cdot e^{\frac{F}{R\cdot T}\cdot(1-f_{ANT})\cdot\Delta\Psi}} - \frac{[ADP_{free}]_{mit}}{[ADP_{free}]_{mit} + [ATP_{free}]_{mit} \cdot e^{-\frac{F}{R\cdot T}\cdot f_{ANT}\cdot\Delta\Psi}} \right)$ $f_{ANT} = 0.65$	
Phosphate translocator (PiC)		
	v_{PiC}	Rate of phosphate translocator (mM ms^{-1})
	k_{PiC}	Rate constant of phosphate translocator, 9.5244 mM ms^{-1}. Model adjusted.
	pK_{PiC}	Dissociation constant for inorganic phosphate, 6.8
	$v_{PiC} = SF_{mit} \cdot k_{PiC} \cdot ([Pi_j]_i \cdot [H^+]_i - [Pi_j]_{mit} \cdot [H^+]_{mit})$ $[Pi_j]_{mit} = \frac{[Pi_{tot}]_{mit}}{1+10^{(pH_{mit}-pK_{PiC})}}$ $[Pi_j]_i = \frac{[Pi_{tot}]_i}{1+10^{(pH_i-pK_{PiC})}}$	
K^+ uniporter (KUni)		
	v_{KUni}	Rate of potassium uniporter (mM ms^{-1})
	k_{KUni}	Rate constant of potassium uniporter, 3.3333×10^{-10} ms^{-1}. Model adjusted.
	$v_{KUni} = SF_{mit} \cdot k_{KUni} \cdot \left([K^+]_i \cdot e^{\frac{F}{R\cdot T}\cdot\frac{-\Delta\Psi}{2}} - [K^+]_{mit} \cdot e^{\frac{F}{R\cdot T}\cdot\frac{\Delta\Psi}{2}} \right)$	
K^+-H^+ exchanger (KHE)		
	v_{KHE}	Rate of K^+/H^+ exchanger (mM ms^{-1})
	k_{KHE}	Rate constant of K^+/H^+ exchanger, 0.0011507 $mM^{-1}ms^{-1}$. Model adjusted to balance pH_{mit}.
	$v_{KHE} = SF_{mit} \cdot k_{KHE} \cdot ([K^+]_{mit} \cdot [H^+]_i - [K^+]_i \cdot [H^+]_{mit})$	
Mitochondrial Na^+-Ca^{2+} exchange (NCX_{mit})		
	Adapted from Saito et al. (Saito *et al.*, 2016)	
	I_{NCXmit}	Current through mitochondrial Na^+/Ca^{2+} exchanger (pA pF^{-1})
	A_{NCXmit}	Amplitude factor of I_{NCXmit}, 0.34735212 pA pF^{-1} (normalized with membrane capacitance)
	K_dNa_{mit}	Dissociation constant for Na^+ in mitochondria. 38.0 mM
	K_dCa_{mit}	Dissociation constant for Ca^{2+} in mitochondria. 0.020932007 mM
	K_dNa_i	Dissociation constant for Na^+ in cytosol. 32.0 mM

	K_dCa_i	Dissociation constant for Ca^{2+} in cytosol. 0.0125 mM
	α, β	Rate constants in the reduced model
	$I_{NCXmit} = -SF_{mit} \cdot A_{NCXmit} \cdot ((1.0 - P(E_1total)) \cdot k_2 - P(E_1total) \cdot k_1)$ $P(E_2Na) = \frac{1}{1 + \left(\frac{K_dNa_i}{[Na^+]_i}\right)^3 \cdot \left(1 + \frac{[Ca^{2+}]_i}{K_dCa_i}\right)}, \quad P(E_1Na) = \frac{1}{1 + \left(\frac{K_dNa_{mit}}{[Na^+]_{mit}}\right)^3 \cdot \left(1 + \frac{[Ca^{2+}]_{mit}}{K_dCa_{mit}}\right)}$ $P(E_2Ca) = \frac{1}{1 + \left(\frac{K_dCa_i}{[Ca^{2+}]_i}\right) \cdot \left(1 + \frac{[Na^+]_i}{K_dNa_i}\right)^3}, \quad P(E_1Ca) = \frac{1}{1 + \left(\frac{K_dCa_{mit}}{[Ca^{2+}]_{mit}}\right) \cdot \left(1 + \frac{[Na^+]_{mit}}{K_dNa_{mit}}\right)^3}$ $k_1 = exp\left(0.2 \cdot \frac{\Delta\Psi \cdot F}{R \cdot T}\right) \cdot P(E_1Na), \quad k_2 = exp\left((0.2 - 1) \cdot \frac{\Delta\Psi \cdot F}{R \cdot T}\right) \cdot P(E_2Na)$ $k_3 = P(E_1Ca), \quad k_4 = P(E_2Ca)$ $\alpha = k_2 + k_4, \quad \beta = k_1 + k_3$ Assuming rapid equilibrium, $P(E_1total) = \frac{\alpha}{\alpha + \beta}$	
Ca^{2+} uniporter (CaUni)		
	Adapted from Saito et al. (Saito *et al.*, 2016) and Nguyen et al. (Nguyen *et al.*, 2007).	
	I_{CaUni}	Current through mitochondrial Ca^{2+} uniporter (pA pF^{-1}) (normalized with membrane capacitance)
	A_{CaUni}	Amplitude factor of I_{CaUni}, 21.5165341 pA pF^{-1}
	a_{mit}	0.2
	a_i	0.341
	$I_{CaUni} = -SF_{mit} \cdot A_{CaUni} \cdot \frac{2 \cdot \Delta\Psi \cdot F}{R \cdot T} \cdot \left(\frac{a_{mit} \cdot [Ca^{2+}]_{mit} - a_i \cdot [Ca^{2+}]_i \cdot e^{\left(\frac{-2 \cdot \Delta\Psi \cdot F}{R \cdot T}\right)}}{e^{\left(\frac{-2 \cdot \Delta\Psi \cdot F}{R \cdot T}\right)} - 1}\right)$	
Mitochondrial Na^+-H^+ exchanger (NHE_{mit})		
	Adapted from Saito et al. (Saito *et al.*, 2016) and Nguyen et al. (Nguyen *et al.*, 2007).	
	v_{NHEmit}	Flux through mitochondrial Na^+/H^+ exchanger (mM ms^{-1} pF^{-1}) (normalized with membrane capacitance)
	$v_{max}NHE_{mit}$	Maximum flux, 0.0228375 mM ms^{-1} pF^{-1}
	K_{Namit}	Mitochondrial Na^+ binding constant, 6.0 mM
	K_{Nai}	Cytosolic Na^+ binding constant, 20.0 mM
	K_H	H^+ binding constant, 0.0000001 mM
	K_Hreg	Allosteric H^+ binding constant, 0.0000757 mM

$$v_{NHEmit} = SF_{mit} \cdot v_{max}NHE_{mit} \cdot \frac{\frac{[H^+]_{mit}}{[H^+]_{mit} + K_H reg} \cdot \left(\frac{[Na^+]_{mit} \cdot [H^+]_i}{K_{Namit} \cdot K_H} - \frac{[Na^+]_i \cdot [H^+]_{mit}}{K_{Nai} \cdot K_H} \right)}{1 + \frac{[H^+]_i}{K_H} + \frac{[Na^+]_{mit}}{K_{Namit}} + \frac{[H^+]_i \cdot [Na^+]_{mit}}{K_H \cdot K_{Namit}} + \frac{[H^+]_{mit}}{K_H} + \frac{[Na^+]_i}{K_{Nai}} + \frac{[H^+]_{mit} \cdot [Na^+]_i}{K_H \cdot K_{Nai}}}$$

Mitochondrial permeability transition pore (mPTP)

Modified Wacquier et al. (Wacquier *et al.*, 2020).

$P(O)_{mPTP}$	Probability of mPTP in open state.
V_{op}	Rate constant of mPTP opening, $0.07 \cdot 10^{-3}$ ms^{-1}
q_{op}	Voltage and mitochondrial Ca^{2+} dependence coefficient of PTP opening, 12000 mV mM^{-1}
q_{11}	Voltage dependence coefficient of PTP opening, 18 mV. Model adjusted.
k_{cl}	Rate constant for mPTP closure, $0.02 \cdot 10^{-3}$ ms^{-1}
$I_{mPTP}Ca$	Ca^{2+} current through mPTP (pA pF^{-1})
$I_{mPTP}H$	H^+ current through mPTP (pA pF^{-1})
$P_{mPTP}Ca$	Permeability of Ca^{2+} through mPTP, 3.551 pA pF^{-1} mM^{-1}. Model adjusted.
$P_{mPTP}H$	Permeability of H^+ through mPTP, 3196 pA pF^{-1} mM^{-1}. Model adjusted.

$$\frac{dP(O)_{mPTP}}{dt} = V_{op} \cdot \frac{1}{1 + e^{\frac{-\Delta\Psi - q_{op} \cdot [Ca^{2+}]_{mit}}{q_{11}}}} \cdot (1 - P(O)_{mPTP}) - k_{cl} \cdot P(O)_{mPTP}$$

$$I_{mPTP}Ca = SF_{mit} \cdot P_{mPTP}Ca \cdot P(O)_{mPTP} \cdot CFmit_{Ca}$$

$$I_{mPTP}H = SF_{mit} \cdot P_{mPTP}H \cdot P(O)_{mPTP} \cdot CFmit_H$$

Differential equations

Adapted from Dzbek and Korzeniewski (Dzbek & Korzeniewski, 2008) and Saito et al. (Saito *et al.*, 2016).

Buf_{NAD}	NAD buffering capacity coefficient, 5.0.
$[Ca^{2+}_{tot}]_{mit}$	Total concentration of mitochondrial Ca^{2+} (mM)

$$\frac{d[NADH]}{dt} = \frac{v_{DH} - v_{C1}}{R_{mc} \cdot Buf_{NAD}}$$

$$\frac{d[UQH_2]}{dt} = \frac{v_{C1} - v_{C3}}{R_{mc}}$$

$$\frac{d[Cytc_r]}{dt} = \frac{v_{C3} - v_{C4}}{R_{mc}}$$

$$\frac{d[H^+]_{mit}}{dt} = \left(\frac{-5 \cdot v_{C1} - 2 \cdot v_{C3} - 4 \cdot v_{C4} + v_{DH} + (n_{ATP} - 1) \cdot \ v_{SN} + v_{PiC} + v_{HLeak} + v_{KHE} + v_{ANT} + v_{NHEmit}}{R_{mc}} - \frac{I_{mPTP}H}{F \cdot V_{mit}} \right) \cdot \frac{1}{Rbuf_{mit}}$$

$$\frac{d[K^+]_{mit}}{dt} = \frac{v_{KUni} - v_{KHE}}{R_{mc}}$$

$$\frac{d[ATP_{tot}]_{mit}}{dt} = \frac{v_{SN} - v_{ANT}}{R_{mc}}$$

$$\frac{d[Pi_{tot}]_{mit}}{dt} = \frac{v_{PiC} - v_{SN}}{R_{mc}}$$

$$\frac{d\Delta\Psi}{dt} = \left(\frac{-4 \cdot v_{C1} - 2 \cdot v_{C3} - 4 \cdot v_{C4} + n_{ATP} \cdot v_{SN} + v_{ANT} + v_{HLeak} + v_{KUni}}{R_{mc}} + \frac{-I_{CaUni} - I_{NCXmit} - I_{mPTP}H - I_{mPTP}Ca}{F \cdot V_{mit}}\right) \cdot \frac{1}{C_{mit}}$$

$$\frac{d[Ca_{tot}^{2+}]_{mit}}{dt} = \frac{-I_{CaUni} + 2 \cdot I_{NCXmit} - I_{mPTP}Ca}{2 \cdot F \cdot V_{mit}}$$

$$[Ca^{2+}]_{mit} = \frac{\sqrt{b^2 + 4 \cdot c} - b}{2}$$

$$b = B_{total} - [Ca_{tot}^{2+}]_{mit} + K_dCa_{mit}, \qquad c = K_dCa_{mit} \cdot [Ca_{tot}^{2+}]_{mit}$$

$$\frac{d[Na^{+}]_{mit}}{dt} = \frac{-3 \cdot I_{NCXmit}}{F \cdot V_{mit}} - \frac{v_{NHEmit}}{R_{mc}}$$

Energy metabolism balance in cytosol

CK : Creatine kinase		
	Adopted from Kuzumoto et al. (Kuzumoto *et al.*, 2008).	
	$phosphocreatine + ADP + H^{+} \underset{k_{bCK}}{\overset{k_{fCK}}{\rightleftharpoons}} creatine + ATP$	
	K_{CK}	Equilibrium constant of CK, $1.6600 \cdot 10^{6}$ mM^{-1}
	k_{fCK}	Forward rate constant of CK, 16.0483 $mM^{-2}\,ms^{-1}$
	k_{bCK}	Backward rate constant of CK, $= k_{f\_CK}/K_{CK} =$ $9.6677 \cdot 10^{-6}$ $mM^{-1}\,ms^{-1}$
	J_{CK}	Flux of CK (mM ms^{-1})
	$[ATP_{tot}]_i$	Total concentration of cytosolic ATP (mM)
	$[ADP_{tot}]_i$	Total concentration of cytosolic ADP (mM)
	$[\mathrm{PCr}]_i$	Concentration of cytosolic phosphocreatine (mM)
	$[\mathrm{Cr}]_i$	Concentration of cytosolic creatine (mM)
	$J_{CK} = [PCr]_i \cdot [ADP_{tot}]_i \cdot [H^{+}]_i \cdot k_{fCK} - [Cr]_i \cdot [ATP_{tot}]_i \cdot k_{bCK}$<br>$\mathrm{dATP}_{CK} = J_{CK}$, $\mathrm{dADP}_{CK} = -J_{CK}$, $\mathrm{dCr}_{CK} = J_{CK}$	
AK : Adenylate kinase		
	Adopted from Kuzumoto et al. (Kuzumoto *et al.*, 2008).	
	$ADP_{free} + ADP_{Mg} \underset{k_{bAK}}{\overset{k_{fAK}}{\rightleftharpoons}} ATP_{Mg} + AMP$	
	k_{fAK}	Forward rate constant of AK, 0.7833 (mM^{-1} ms^{-1}). Adopted from Thuma et al. (Thuma *et al.*, 1972).
	k_{bAK}	Backward rate constant of AK, 0.6833 (mM^{-1} ms^{-1}). Adopted from Thuma et al. (Thuma *et*

		al., 1972).
	$J_{AK} = [ADP_{free}]_i \cdot [ADP_{Mg}]_i \cdot k_{f_{AK}} - [ATP_{Mg}]_i \cdot [AMP]_i \cdot k_{bAK}$<br>$\mathrm{dATP}_{AK} = J_{AK}, \quad \mathrm{dADP}_{AK} = -2 \cdot J_{AK}$	
Gly : Glycolysis		
	Newly added	
	J_{Gly}	Flux of glycolysis, $1.2 \cdot 10^{-5}$ mM ms^{-1}
	$\mathrm{dATP}_{Gly} = J_{Gly}, \quad \mathrm{dADP}_{Gly} = -J_{Gly}$	
Balance of ATP consumption and production		
	$K_d ATP_i Mg$	Dissociation constant of ATP in cytosol for Mg^{2+}. 0.087 mM. Adopted from Michailova & McCulloch (Michailova & McCulloch, 2001).
	$K_d ATP_i Ca$	Dissociation constant of ATP in cytosol for Ca^{2+}. 0.1698 mM. Adopted from Michailova & McCulloch (Michailova & McCulloch, 2001).
	$K_d ADP_i Mg$	Dissociation constant of ADP in cytosol for Mg^{2+}. 0.676 mM. Adopted from Michailova & McCulloch (Michailova & McCulloch, 2001).
	$K_d ADP_i Ca$	Dissociation constant of ADP in cytosol for Ca^{2+}. 1.548 mM. Adopted from Michailova & McCulloch (Michailova & McCulloch, 2001).
	$[ATP_{Mg}]_i$	Concentration of cytosolic ATP bound to Mg^{2+} (mM)
	$[ATP_{free}]_i$	Concentration of cytosolic free ATP (mM)
	$[ADP_{Mg}]_i$	Concentration of cytosolic ADP bound to Mg^{2+} (mM)
	$[ADP_{free}]_i$	Concentration of cytosolic free ADP (mM)
	$[AMP]_i$	Concentration of AMP in cytosol (mM)
	$[Adenine_{tot}]_i$	Total concentration of adenine nucleotides in cytosol, 7.0 mM<br>$= [AMP]_i + [ADP_{tot}]_i + [ATP_{tot}]_i$
	$[Cre_{tot}]_i$	Total creatine concentrations in cytosol, 25.0 mM<br>$= [phosphocreatine]_i + [Creatine]_i$
	$[Phosph_{tot}]_i$	Total concentrations of phosphate in cytosol, 46.0 mM
	$[Pi_{tot}]_i$	Total concentration of cytosolic inorganic phosphate (mM)
	$dATPuse = -(dATPuse_{NaK} + dATPuse_{SERCA} + dATPuse_{PMCA} + dATPuse_{CNTR})$<br>$dADPuse = -dATPuse$<br>$\frac{d[ATP_{tot}]_i}{dt} = v_{ANT} + \mathrm{dATP}_{CK} + \mathrm{dATP}_{AK} + \mathrm{dATP}_{Gly} + dATPuse$<br>$\frac{d[ADP_{tot}]_i}{dt} = -v_{ANT} + \mathrm{dADP}_{CK} + \mathrm{dADP}_{AK} + \mathrm{dADP}_{Gly} + dADPuse$<br>$\frac{d[PCr]_i}{dt} = -\mathrm{dCr}_{CK}$<br>$[AMP]_i = [Adenine_{tot}]_i - [ADP_{tot}]_i - [ATP_{tot}]_i$<br>$[Cr]_i = [Cre_{tot}]_i - [PCr]_i$<br>$[Pi_{tot}]_i = [Phosph_{tot}]_i - ([PCr]_i + 3.0 \cdot [ATP_{tot}]_i + 2.0 \cdot [ADP_{tot}]_i + [AMP]_i)$	

	$-(3.0 \cdot [ATP_{tot}]_{mit} + 2.0 \cdot [ADP_{tot}]_{mit} + [Pi_{tot}]_{mit}) \cdot R_{mc})$ $[ATP_{free}]_i = \frac{[ATP_{tot}]_i}{1 + \frac{[Mg^{2+}]_i}{K_d ATP_i Mg} + \frac{[Ca^{2+}]_i}{K_d ATP_i Ca}}$ $[ATP_{Mg}]_i = \frac{[Mg^{2+}]_i \cdot [ATP_{free}]_i}{K_d ATP_i Mg}$ $[ADP_{free}]_i = \frac{[ADP_{tot}]_i}{1 + \frac{[Mg^{2+}]_i}{K_d ADP_i Mg} + \frac{[Ca^{2+}]_i}{K_d ADP_i Ca}}$ $[ADP_{Mg}]_i = \frac{[Mg^{2+}]_i \cdot [ADP_{free}]_i}{K_d ADP_i Mg}$

Oxygen consumption and buffering

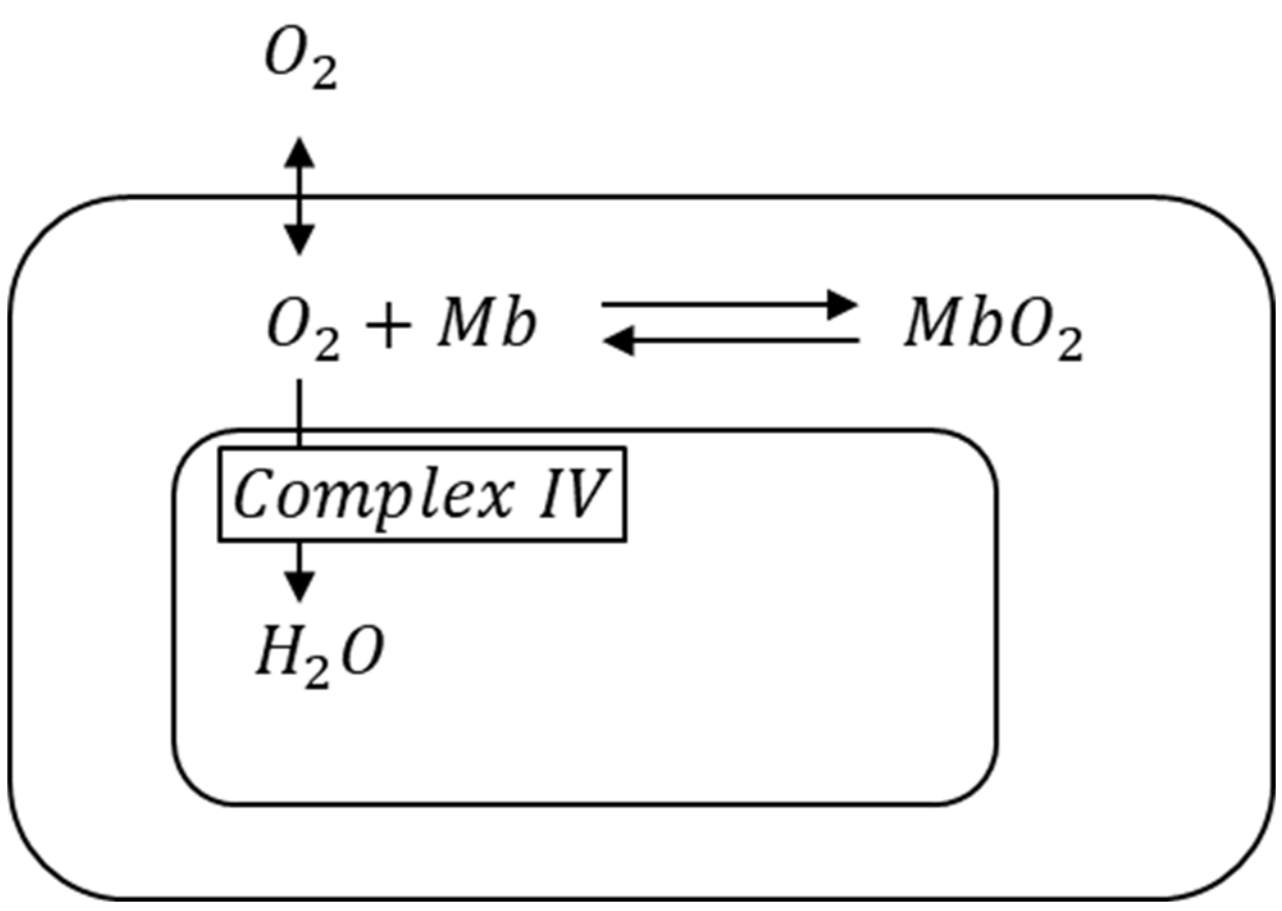

	α_{O2}	Solubility of O_2, $2.4 \cdot 10^{-3}$ mM mmHg^{-1}. Adopted from Hendgen-Cotta et al. (Hendgen-Cotta *et al.*, 2014).
	C_{Mb}	Concentration of myoglobin (Mb), 0.3 mM. Adopted from Hendgen-Cotta et al. (Hendgen-Cotta *et al.*, 2014).
	$P_{50}Mb$	Half saturation of Mb for O_2, 2.8 mmHg. Adopted from Hendgen-Cotta et al. (Hendgen-Cotta *et al.*, 2014).
	K_dMb	Half saturation of Mb for O_2 (mM) $= P_{50}Mb \cdot \alpha_{O2}$
	$[O_2]_i$	Oxygen concentration in cytosol (mM)
	P_{O2}	Membrane permeability to O_2, 0.0985 µm ms^{-1} ($= P_{CO2}/20$)
	J_{O2}	Diffusive flux of O_2 across the sarcolemmal membrane (mM ms^{-1})
	$factor_{Mb} = 1 + C_{Mb} \cdot \frac{K_dMb}{([O_2]_i + K_dMb)^2}$ $J_{O_2} = P_{O2} \cdot \frac{A_i}{V_i} \cdot ([O_2]_o - [O_2]_i)$	

	$$\frac{d[O_2]_i}{dt} = \frac{(J_{O_2} - v_{C4}) \cdot V_i}{(V_i + V_{mit}) \cdot factor_{Mb}}$$

Cytosolic pH buffering

CO2 hydration reaction (CO_2hyd)		
	Adapted from Crampin and Smith (Crampin & Smith, 2006). CO_2 production via mitochondrial substrate hydration was newly added.	
	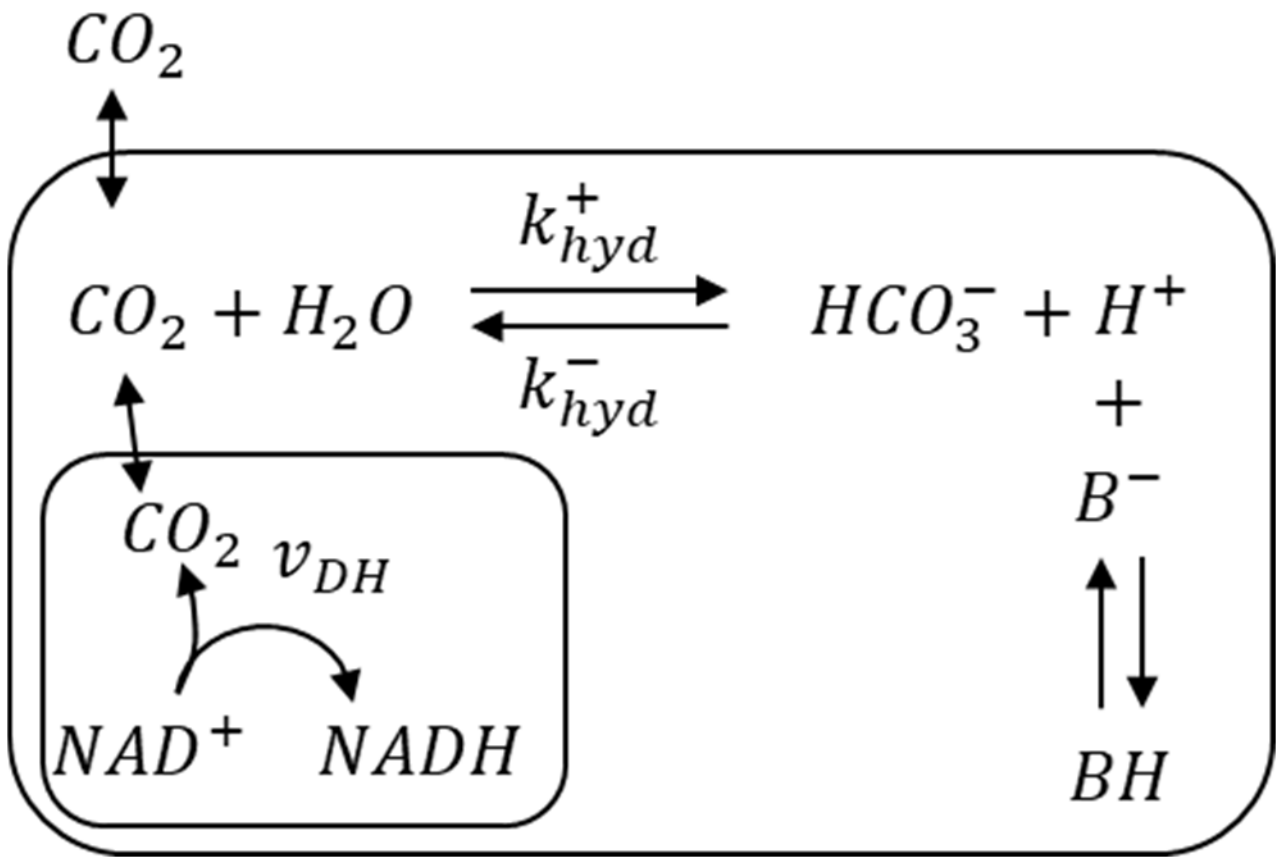	
	k^+_{hyd}	CO_2 hydration forward rate constant, $3.65 \cdot 10^{-4}$ ms^{-1}
	k^-_{hyd}	CO_2 hydration backward rate constant, 0.480 $mM^{-1}ms^{-1}$
	P_{CO2}	Membrane permeability to CO_2, 1.97 μm ms^{-1}
	J_{CO2}	Diffusive flux of CO_2 across the sarcolemmal membrane (mM ms^{-1})
	J_{CO_2hyd}	Flux of CO_2 hydration (mM ms^{-1})
	$J_{CO_2hyd} = k^+_{hyd} \cdot [CO_2]_i - k^-_{hyd} \cdot [HCO_3^-]_i \cdot [H^+]_i$<br>$J_{CO_2} = P_{CO2} \cdot \frac{A_i}{V_i} \cdot ([CO_2]_o - [CO_2]_i)$<br>$\frac{d[CO_2]_i}{dt} = J_{CO_2} - J_{CO_2hyd} + 0.75 \cdot v_{DH}$<br>$CO_2$ production via substrate dehydrogenation ($0.75 \cdot v_{DH}$) was model-adjusted to maintain pH_i and pH_{mit} within reported ranges.	
Intrinsic pH buffering		
	Adapted from Crampin and Smith (Crampin & Smith, 2006).	
	$B1$	Total concentration of intrinsic buffer species 1, 84.2 mM. Adopted from Crampin and Smith (Crampin & Smith, 2006).
	$pK1cyt$	Dissociation constant of intrinsic buffer species 1 for H^+, 6.03. Adopted from Crampin and Smith (Crampin & Smith, 2006).
	$B2$	Total concentration of intrinsic buffer species 2, 29.4 mM. Adopted from Crampin and

		Smith (Crampin & Smith, 2006).
	$pK2cyt$	Dissociation constant of intrinsic buffer species 2 for H^+, 7.57. Adopted from Crampin and Smith (Crampin & Smith, 2006).
	$buffers$	H^+ buffer in cytosol
	$HfromMit$	H^+ flux from mitochondria (mM ms^{-1})
	$VHeff$	H^+ efflux from cytosol (mM ms^{-1})
	n_{ATP}	ATP synthase proton stoichiometry, 2.5. See “Mitochondria” section.
	$dATP_{CK}$	See “Energy metabolism balance in cytosol” section.
	$dATP_{Gly}$	See “Energy metabolism balance in cytosol” section.
	$dATP_{use}$	See “Energy metabolism balance in cytosol” section.
	$\frac{d[H^+]_i}{dt} = \frac{\frac{(J_{CHE} - J_{NHE})}{V_i} + J_{CO_2hyd} - (dATP_{CK} + dATP_{use} + HfromMit + VHeff)}{buffers}$<br>$buffers = 1 + B1 \cdot \frac{1000 \cdot 10^{-pK1cyt}}{(1000 \cdot 10^{-pK1cyt} + [H^+]_i)^2} + B2 \cdot \frac{1000 \cdot 10^{-pK2cyt}}{(1000 \cdot 10^{-pK2cyt} + [H^+]_i)^2}$<br>$HfromMit = -4 \cdot v_{C1} - 4 \cdot v_{C3} - 2 \cdot v_{C4} + n_{ATP} \cdot v_{SN} + v_{PiC} + v_{HLeak} + v_{KHE} + v_{ANT} + v_{NHEmit} - \frac{I_{mPTP}H}{F \cdot V_i}$<br>$VHeff = \begin{cases} 0.0 & (\mathrm{pH_i} > \mathrm{pH_o}) \\ 0.37635 \cdot ([\mathrm{H}^+]_i - [\mathrm{H}^+]_o) & (\mathrm{pH_i} \le \mathrm{pH_o}) \end{cases}$	

Calculation of the membrane potential and cytosolic ion concentrations

Membrane potential (mV)		
	I_{tot}	Total current of ion channels and ion transporters (pA pF^{-1})
	I_{stim}	Stimulation current, 28.409 pA pF^{-1}
	$I_{tot} = I_{Na} + I_{NaL} + I_{CaL} + I_{CaT} + I_{K1} + I_{Kr} + I_{Ks} + I_{to} + I_{NCX} + I_{NaK} + I_{Cab} + I_{l(Ca)} + I_{NSC} + I_{Kpl} + I_{PMCA} + I_{KATP} + I_{Clb}$<br>$I_cNa = I_{Na}Na + I_{NaL}Na + I_{CaL}Na + I_{Ks}Na + I_{to}Na + I_{NCX}Na + I_{NaK}Na + I_{l(Ca)}Na + I_{NSC}Na$<br>$I_cK = I_{Na}K + I_{CaL}K + I_{K1}K + I_{Kr}K + I_{Ks}K + I_{to}K + I_{NaK}K + I_{l(Ca)}K + I_{NSC}K + I_{Kpl}K + I_{KATP}K$<br>$I_cCa = I_{CaL}Ca + I_{CaT}Ca + I_{NCX}Ca + I_{Cab}Ca + I_{PMCA}Ca$<br>$I_cCl = I_{Clb}Cl$<br>$\frac{dV_m}{dt} = -(I_{tc} + I_{stim})$	
Internal ion concentrations		
	$[Ca^{2+}_{tot}]_i$	total concentration of cytosolic Ca^{2+} (mM)
	$\frac{d[Na^+]_i}{dt} = -\frac{I_cNa \cdot C_m}{F \cdot V_i} + \frac{(J_{NKCC1} + J_{NHE} + J_{NBC})}{V_i} - \left(-3.0 \cdot \frac{I_{NCXmit}}{F \cdot V_i} - v_{NHEmit}\right)$<br>$\frac{d[K^+]_i}{dt} = -\frac{(I_cK + I_{stim}) \cdot C_m}{F \cdot V_i} + \frac{J_{NKCC1}}{V_i} - (v_{KUni} - v_{KHE})$	

	$$\frac{d[Ca^{2+}_{tot}]_i}{dt} = -\frac{\left(I_cCa + I_{SERCA} - Caleak_{SR} - I_{RyR}\right)\cdot C_m}{2.0\cdot F\cdot V_i} + TrnCaFlux - \frac{(-I_{CaUni} + 2.0\cdot I_{NCXmit} - I_{mPTP}Ca)}{2.0\cdot F\cdot V_i}$$ $$[Ca^{2+}]_i = \frac{\sqrt{b^2 + 4\cdot c} - b}{2}$$ $$b = B_{max}CaM + K_dCaM - [Ca^{2+}_{tot}]_i, \quad c = K_dCaM\cdot[Ca^{2+}_{tot}]_i$$ $$\frac{d[Cl^-]_i}{dt} = \frac{I_cCl\cdot C_m}{F\cdot V_i} + \frac{(2\cdot J_{NKCC1} + J_{CHE} + J_{AE})}{V_i}$$ $$\frac{d[HCO_3^-]_i}{dt} = J_{CO_2hyd} + \frac{(J_{NBC} - J_{AE})}{V_i}$$

Simulation of ischemia and reperfusion

Extracellular oxygen concentration $[O_2]_o$ (mM)		
	τ_{i1}	Time constant for extracellular O_2 decline during ischemia, fast component, 8,000 ms
	τ_{i2}	Time constant for extracellular O_2 decline during ischemia, slow component, 8,000,000 ms
	τ_r	Time constant for extracellular O_2 recovery during reperfusion, 60,000 ms
	t_i	Time when ischemia begins, 180,000 ms (= 3 min)
	t_r	Time when reperfusion begins, 1,080,000 ms (= 18 min)
	Normoxia: $[O_2]_o = 0.24$<br>Ischemia: $[O_2]_o = 0.24\cdot\left(0.99\cdot e^{\left(\frac{t_i - t}{\tau i1}\right)} + 0.01\cdot e^{\left(\frac{t_i - t}{\tau i2}\right)}\right) \quad t: time\ (ms)$<br>Reperfusion: $[O_2]_o = [O_2]_{o\_i} + \left(0.24 - [O_2]_{o\_i}\right)\cdot\left(1.0 - e^{\left(\frac{t_r - t}{\tau_r}\right)}\right) \quad [O_2]_{o\_i}: [O_2]_o\ at\ the\ end\ of\ ischemia$	
Extracellular ion and carbon dioxide concentration $[CO_2]_o$ (mM)		
	Normoxia:<br>$$\frac{d[Na^+]_o}{dt} = 0.0$$ $$\frac{d[K^+]_o}{dt} = 0.0$$ $$\frac{d[Ca^{2+}]_o}{dt} = 0.0$$ $$\frac{d[Cl^-]_o}{dt} = 0.0$$ $$\frac{d[CO_2]_o}{dt} = 0.0$$ Ischemia: extracellular ion and CO_2 accumulation (+)<br>$$\frac{d[Na^+]_o}{dt} = \frac{\frac{IcNa}{F} - (J_{NKCC1} + J_{NHE} + J_{NBC})}{V_o}$$	

$$\frac{d[K^+]_o}{dt} = \frac{\frac{IcK}{F} - J_{NKCC1}}{V_o}$$

$$\frac{d[Ca^{2+}]_o}{dt} = \frac{\frac{IcCa}{2 \cdot F}}{V_o}$$

$$\frac{d[Cl^-]_o}{dt} = \frac{\frac{-IcCl}{F} - (2 \cdot J_{NKCC1} + J_{CHE} + J_{AE})}{V_o}$$

$$\frac{d[CO_2]_o}{dt} = \frac{J_{CO_2} \cdot V_i}{V_o}$$

Reperfusion:

$$\frac{d[Na^+]_o}{dt} = \frac{(140.0 - [Na^+]_o)}{\tau_r}$$

$$\frac{d[K^+]_o}{dt} = \frac{(5.4 - [K^+]_o)}{\tau_r}$$

$$\frac{d[Ca^{2+}]_o}{dt} = \frac{(1.8 - [Ca^{2+}]_o)}{\tau_r}$$

$$\frac{d[Cl^-]_o}{dt} = \frac{(140.0 - [Cl^-]_o)}{\tau_r}$$

$$\frac{d[CO_2]_o}{dt} = \frac{(1.236 - [CO_2]_o)}{\tau_r}$$

Intracellular H^+ concentration $[H^+]_i$ (mM)

	$HMetab$	H^+ production during ischemia (mM ms^{-1})

$$\frac{d[H^+]_i}{dt} = \frac{\frac{(J_{CHE} - J_{NHE})}{V_i} + J_{CO_2hyd} - (dATP_{CK} + dATP_{use} + HfromMit + VHeff - HMetab)}{buffers}$$

Normoxia: $HMetab = 0$

Ischemia: $HMetab = 7.2 \cdot 10^{-6} \cdot \left(1 - 0.99 \cdot e^{\left(\frac{t_i - t}{\tau i1}\right)} + 0.01 \cdot e^{\left(\frac{t_i - t}{\tau i2}\right)}\right)$

Reperfusion: $HMetab = HMetab_{\_i} \cdot e^{\left(\frac{t_r - t}{\tau_r}\right)}, (HMetab_{\_i}: HMetab\ at\ the\ end\ of\ ischemia)$

Scaling factor of mitochondrial component (SF_{mit})

$$SF_{mit} = \begin{cases} 1.0 & normoxia\ and\ ischemia \\ 0.5 & reperfusion \end{cases}$$

Simulation of respiratory acidosis

Adopted from Crampin and Smith (Crampin & Smith, 2006)

$$[CO_2]_o = \alpha_{Co2} \cdot 760 \cdot \frac{\%CO_2}{100}, \quad \begin{cases} \%CO_2 = 5\%\ (t < 300{,}000\ and\ t > 900{,}000) \\ \%CO_2 = 30\%\ (300{,}000 \le t \le 900{,}000) \end{cases}$$

II. Initial values of variables; stimulus every 400 ms

	$P(RP)_{INa}$	0.4363232829102012
	$P(AP)_{INa}$	$1.815342743824861 \cdot 10^{-5}$
	$P(AI)_{INa}$	0.3474111552452
	$P(Is)_{INa}$	0.6896076955238272
	$P(y)_{INaL}$	0.006790707336817402
	$P(RP)_{ICaL}$	0.9997223583020974
	$P(AP)_{ICaL}$	$1.009391398951725 \cdot 10^{-66}$
	$P(AI)_{ICaL}$	$6.617178702369459 \cdot 10^{-5}$
	$P(U)_{ICaL}$	0.2081195087970697
	$P(UCa)_{ICaL}$	$9.14629662694451 \cdot 10^{-5}$
	$P(C)_{ICaL}$	0.511528387140029
	$P(Is)_{ICaL}$	0.9998941027561881
	$P(y_1)_{ICaT}$	$1.603385040803366 \cdot 10^{-5}$
	$P(y_2)_{ICaT}$	0.867489248065955
	$P(O)_{IK1}$	0.4312446488200912
	$P(y_1)_{IKr}$	0.000846413992283203
	$P(y_2)_{IKr}$	0.09412869764184509
	$P(y_3)_{IKr}$	0.9684289852394083
	$P(y_1)_{IKs}$	0.09722919852729853
	$P(y_2)_{IKs}$	0.4839159979592927
	$P(y_{C2})_{IKs}$	0.07481435026531641
	$P(y_1)_{Ito}$	0.000785486173894794
	$P(y_2)_{Ito}$	0.9999153092686555
	$P(E_1 total)$	0.1469501296927564
	$P(I_1)$	0.2604815445486064
	$P(I_2)$	0.5920362478286904
	$P(y)_{PMCA}$	0.3792758289141482
	$[TCa]$	0.00306359169376361
	$[TCa^*]$	0.0001512192536252197
	$[T^*]$	$3.920470776404132 \cdot 10^{-5}$
	$hsmL$	1.04165083799362
	$hsmX$	1.036631816396675
	$[ADP_{tot}]_i$	0.07204085139306565
	$[ATP_{tot}]_i$	6.927743454112318
	$[PCr]_i$	10.83213188661045
	V_m	−86.24537229578506

	$[Ca^{2+}_{tot}]_i$	0.001917069404003856
	$[Na^+]_i$	7.626279644335617
	$[K^+]_i$	138.8203589557546
	$[Cl^-]_i$	34.47152461406506
	$[H^+]_i$	$7.164586119063776 \cdot 10^{-5}$
	$[CO_2]_i$	1.236265119202441
	$[HCO_3^-]_i$	13.09881579113231
	$[Na^+]_o$	140
	$[K^+]_o$	5.4
	$[Ca^{2+}]_o$	1.8
	$[Cl^-]_o$	140
	$[CO_2]_o$	1.23614
	$P(y)_{SERCA}$	0.0323021120978837
	$[Ca^{2+}_{tot}]_{jSR}$	7.391095291193319
	$[Ca^{2+}]_{nSR}$	1.28507101369781
	$P(close)_{RyR}$	0.1327254395928346
	$P(open)_{RyR}$	0.0001587380845201006
	$[Cytc_r]$	0.05460038292272422
	$[UQH_2]$	0.2640547922514951
	$[NADH]$	1.520471041697276
	$[K^+]_{mit}$	51.79423459280966
	$[ATP_{tot}]_{mit}$	6.388451955125213
	$[Pi_{tot}]_{mit}$	4.186345597324941
	$[H^+]_{mit}$	$2.147309402406703 \cdot 10^{-5}$
	$\Delta\Psi$	−155.3202955072871
	$[Ca^{2+}_{tot}]_{mit}$	0.2458640916093168
	$[O_2]_i$	0.236673868547168
	$P(O)_{mPTP}$	0.001000343804167244
	$[Na^+]_{mit}$	0.6961721454607275

III. References

Crampin, E.J. & Smith, N.P. (2006). A dynamic model of excitation-contraction coupling during acidosis in cardiac ventricular myocytes. *Biophys J* **90,** 3074-3090.

Dzbek, J. & Korzeniewski, B. (2008). Control over the contribution of the mitochondrial membrane potential (ΔΨ) and proton gradient (ΔpH) to the protonmotive force (Δp). In silico studies. *J Biol Chem* **283,** 33232-33239.

Gaur, N., Rudy, Y. & Hool, L. (2009). Contributions of ion channel currents to ventricular action potential changes and induction of early afterdepolarizations during acute hypoxia. *Circ Res* **105,** 1196-1203.

Hendgen-Cotta, U.B., Kelm, M. & Rassaf, T. (2014). Myoglobin functions in the heart. *Free Radic Biol Med* **73,** 252-

259.

Kuzumoto, M., Takeuchi, A., Nakai, H., Oka, C., Noma, A. & Matsuoka, S. (2008). Simulation analysis of intracellular Na^+ and Cl^- homeostasis during β1-adrenergic stimulation of cardiac myocyte. *Prog Biophys Mol Biol* **96,** 171-186.

Michailova, A. & McCulloch, A. (2001). Model study of ATP and ADP buffering, transport of Ca^{2+} and Mg^{2+}, and regulation of ion pumps in ventricular myocyte. *Biophys J* **81,** 614-629.

Nguyen, M.H., Dudycha, S.J. & Jafri, M.S. (2007). Effect of Ca^{2+} on cardiac mitochondrial energy production is modulated by Na^+ and H^+ dynamics. *Am J Physiol Cell Physiol* **292,** C2004-2020.

Oka, C., Cha, C.Y. & Noma, A. (2010). Characterization of the cardiac Na^+/K^+ pump by development of a comprehensive and mechanistic model. *J Theor Biol* **265,** 68-77.

Saito, R., Takeuchi, A., Himeno, Y., Inagaki, N. & Matsuoka, S. (2016). A simulation study on the constancy of cardiac energy metabolites during workload transition. *J Physiol* **594,** 6929-6945.

Scully, P.R., Bastarrika, G., Moon, J.C. & Treibel, T.A. (2018). Myocardial extracellular volume quantification by cardiovascular magnetic resonance and computed tomography. *Curr Cardiol Rep* **20,** 15.

Shim, E.B., Amano, A., Takahata, T., Shimayoshi, T. & Noma, A. (2007). The cross-bridge dynamics during ventricular contraction predicted by coupling the cardiac cell model with a circulation model. *J Physiol Sci* **57,** 275-285.

Thuma, E., Schirmer, R.H. & Schirmer, I. (1972). Preparation and characterization of a crystalline human ATP:AMP phosphotransferase. *Biochim Biophys Acta* **268,** 81-91.

Wacquier, B., Combettes, L. & Dupont, G. (2020). Dual dynamics of mitochondrial permeability transition pore opening. *Sci Rep* **10,** 3924.
