# Supplementary material for: Integrative simulation analysis of myocardial ischaemia–reperfusion injury
Source: J Physiol. 2026 Aug 20;604(18):7640–62. doi: 10.1113/JP290440 (PMC13577699; doi:10.1113/JP290440)
Supplement: Supplementary file 2 [file TJP-604-7640-s002.pdf]

The Journal of Physiology

Integrative simulation analysis of myocardial ischemia-reperfusion injury

Takao Shimayoshi, Ayako Takeuchi, and Satoshi Matsuoka
DOI: 10.1113/JP290440

Corresponding author(s): Ayako Takeuchi (a-takeuchi@okayama-u.ac.jp), Takao Shimayoshi (simayosi@s.okayama-u.ac.jp)

The referees have opted to remain anonymous.

Review Timeline:

Submission Date:	02-Nov-2025
Editorial Decision:	08-Dec-2025
Revision Received:	09-Jun-2026
Editorial Decision:	24-Jun-2026
Revision Received:	25-Jun-2026
Accepted:	01-Jul-2026

Senior Editor: Natalia Trayanova

Reviewing Editor: T Alexander Quinn

Transaction Report:

(Note: With the exception of the correction of typographical or spelling errors that could be a source of ambiguity, letters and reports are not edited. Depending on transfer agreements, referee reports obtained elsewhere may or may not be included in this compilation. Referee reports are anonymous unless the Referee chooses to sign their reports.)

Re: JP-RP-2025-290440 "Integrative simulation analysis of myocardial ischemia-reperfusion injury" by Takao Shimayoshi, Ayako Takeuchi, and Satoshi Matsuoka

Dear Dr Takeuchi,

Thank you for submitting your manuscript to The Journal of Physiology. It has been assessed by a Reviewing Editor and by 2 expert referees and we are pleased to tell you that it is potentially acceptable for publication following satisfactory major revision.

Please advise your co-authors of this decision as soon as possible.

The referee reports are copied at the end of this email.

Please address all the points raised and incorporate all requested revisions or explain in your Response to Referees why a change has not been made. We hope you will find the comments helpful and that you will be able to return your revised manuscript within 2 months. If your article is NOT for a Special Issue, you may have 9 months to revise. If you require an extension, please contact journal staff: jp@physoc.org. Please note that this letter does not constitute a guarantee for acceptance of your revised manuscript.

Your revised manuscript should be submitted online using the link in your Author Tasks: Link Not Available. This link is accessible via your account as Corresponding Author; it is not available to your co-authors. If this presents a problem, please contact journal staff (jp@physoc.org). Image files from the previous version are retained on the system. Please ensure you replace or remove any files that are being revised.

If you do not wish to submit a revised version of your manuscript, you must inform our journal staff (jp@physoc.org) or reply to this email to request withdrawal. Please note that a manuscript must be formally withdrawn from the peer review process at one journal before it may be submitted to another journal.

TRANSPARENT PEER REVIEW POLICY: To improve the transparency of its peer review process, The Journal of Physiology publishes online as supporting information the peer review history of all articles accepted for publication. Readers will have access to decision letters, including Editors' comments and referee reports, for each version of the manuscript, as well as any author responses to peer review comments. Referees can decide whether or not they wish to be named on the peer review history document.

ABSTRACT FIGURES: Authors are expected to use The Journal's premium BioRender account to create/redraw their Abstract Figures. Information on how to access this account is here: https://physoc.onlinelibrary.wiley.com/journal/14697793/biorender-access.

This will enable Authors to create and download high-resolution figures. If authors have used the free BioRender service, they can use the instructions provided in the link above to download a high-resolution version suitable for publication.

The link provided should only be used for the purposes of this submission. Authors will be charged for figures created on this account if they are not related to this manuscript submission.

LANGUAGE EDITING AND SUPPORT FOR PUBLICATION: If you would like help with English language editing, or other article preparation support, Wiley Editing Services offers expert help, including English Language Editing, as well as translation, manuscript formatting, and figure formatting at www.wileyauthors.com/eeo/preparation. You can also find resources for Preparing Your Article for general guidance about writing and preparing your manuscript at www.wileyauthors.com/eeo/prepresources.

RIGOUR AND REPRODUCIBILITY: We recommend authors consult The Journal's Rigour and Reproducibility requirements to ensure that their manuscript is suitable for publication: https://physoc.onlinelibrary.wiley.com/pb-assets/hub-assets/physoc/documents/TJP-Rigour-and-Reproducibility-Requirements.pdf

REVISION CHECKLIST:

Check that your Methods section conforms to journal policy: https://jp.msubmit.net/cgi-bin/main.plex?form_type=display_requirements#methods.

Check that data presented conforms to the statistics policy: https://jp.msubmit.net/cgi-bin/main.plex?form_type=display_requirements#statistics.

Upload a full Response to Referees file. To create your 'Response to Referees': copy all the reports, including any comments from the Senior and Reviewing Editors, into a Microsoft Word, or similar, file and respond to each point, using font or background colour to distinguish comments and responses and upload as the required file type.

Please upload two versions of your manuscript text: one with all relevant changes highlighted and one clean version with no changes tracked. The manuscript file should include all tables and figure legends, but each figure/graph should be uploaded as separate, high-resolution files.

You may also upload:

- 'Potential Cover Art' for consideration as the issue's cover image
- Appropriate Supporting Information (Video, audio or data set: see https://jp.msubmit.net/cgi-bin/main.plex?form_type=display_requirements#supp).

We look forward to receiving your revised submission.

If you have any queries, please reply to this email and we will be pleased to advise.

Yours sincerely,

Natalia Trayanova
Senior Editor
The Journal of Physiology

REQUIRED ITEMS FOR REVISION

- Author photo and profile. First or joint first authors are asked to provide a short biography (no more than 100 words for one author or 150 words in total for joint first authors) and a portrait photograph. These should be uploaded and clearly labelled together in a Word document with the revised version of the manuscript. See Information for Authors for further details.

- Please upload separate high-quality figure files via the submission form.

- Your paper contains Supporting Information of a type that we no longer publish, including supplementary tables and figures. Any information essential to an understanding of the paper must be included as part of the main manuscript and figures. The only Supporting Information that we publish are video and audio, 3D structures, program codes and large data files. Your revised paper will be returned to you if it does not adhere to our Supporting Information Guidelines.

- Please include an Abstract Figure file and an Abstract Figure legend. An appropriate figure legend, which should not exceed 150 words in length, should be included in the main manuscript file. The Abstract Figure is a piece of artwork designed to give readers an immediate understanding of the research and should summarise the main conclusions. If possible, the image should be easily 'readable' from left to right or top to bottom. It should show the physiological relevance of the manuscript so readers can assess the importance and content of its findings. Abstract Figures should not merely recapitulate other figures in the manuscript. Please try to keep the diagram as simple as possible and without superfluous information that may distract from the main conclusion(s). Abstract Figures must be provided by authors no later than the revised manuscript stage and should be uploaded as a separate file during online submission labelled as File Type 'Abstract Figure'. Please also ensure that you include the figure legend in the main article file. All Abstract Figures should be created using BioRender. Authors should use The Journal's premium BioRender account to export high-resolution images. Details on how to use and access the premium account are included as part of this email.

- Please include a full title page as part of your main article (Word) file, which should contain the following: title, authors, affiliations, corresponding author name and contact details, keywords, and running title.

EDITOR COMMENTS

Reviewing Editor:

You paper has been reviewed by two experts in the field, who felt it is novel and addresses an important area, making it a potentially valuable scientific contribution. There were, however, significant limitations identified that must be addressed, as outlined in the Comments for the Author. In particular, there were concerns related to: (i) how changes in oxidative phosphorylation were modelled; (ii) important physiological effects that were not captured by the model; (ii) the timing of ischaemic effects and the magnitude of some physiological changes compared to experimental evidence; and (iv) what species was being modelled, which is important as effects of ischaemia vary between species. In addition, it was felt the paper would be stronger if novel hypotheses generated by the model for future experimental work were discussed, along with a need for further clarification of some aspects of the study and other minor suggestions. If the authors feel they can adequately address the reviewers concerns they are encouraged to submit a revised version of the paper, along with a point-by-point response.

REFEREE COMMENTS

Referee #1:

The computational model presented in the manuscript of Shimayoshi et al. addresses an important challenge that has not been dealt with this type of modeling. Namely the simulation of the electrophysiological, mechanical and metabolic changes occurring during episodes of ischemia followed by reperfusion. The novelty in this modeling instance is given by the pH dependence of various transport processes across the plasma membrane together with a description encompassing mitochondrial energetics. The incorporation of the dynamics of the ROS that are believed to play an important role in the ischemia reperfusion injury is also novel. Previous attempts have failed to incorporate the dynamics of energy supply by mitochondrial activity. I would commend the authors for undertaking this modelling problem.

I have several suggestions to improve the modeling and the presentation thereof:

1. Regarding the modeling, it is never mentioned, which is the cardiac system that the authors are modeling. This is important because there are key differences between rodents E-C Coupling and other mammals (e.g. canine, pig, etc). The percentage of the Ca2+ recycled in each beat is completely different and the contrast with an experimental model result may be largely dependent on such Ca2+ dynamical behavior.

2. The dynamics of contractile activity decline during ischemia (total flow ischemia) in isolated hearts is much faster than 20 minutes (see <2 min in Lyon ... and Akar, 2010, PMID: 20624394; or Laclau et al. 2001 PMID: 11343417 reporting a contracture of the heart after 10 min ischemia) although the action potential decrease in amplitude is slower. Concerning the appearance of metabolic changes, Gudbjarnason et al, (1970) PMID: 5519941 reported that CrP falls after only 1 minute of ischemia whereas ATP remained constant in that temporal window. In this reviewer's opinion, there may be a lack of feedback between the Pi released upon ischemia on mitochondrial ATP synthase, or an incorrect parametrization of the creatine kinase activity, but the authors should find why ATP continues to be synthesized when there should be no inner mitochondrial membrane potential as demonstrated in Lyon et al. 2010. This is a serious shortcoming of the model and should be clarified.

3. This reviewer is interested to know about the quantitative comparison of the burst in cytoplasmic superoxide production at the onset of reperfusion with respect to experimental models of IR injury, since, even if transient, 200mM superoxide sounds awfully high for cardiomyocytes function.

4. Despite the still unresolved issues of the redox role in IR injury and the molecular identity of the mPTP, a model can postulate hypotheses that may contribute to clarifying the controversies and suggest experiments to solve the open questions. I believe that at least this should be discussed as a follow up to these modeling studies.

Minor issues

5. A glossary of symbols and abbreviations should be included to ease understanding of the model variables and parameters.

6. Lines 190-191. The authors should include references to justify those arguments.

7. Related to my point #4 the section on redox changes during and after ischemia and their dynamics (as in appendix Figure A6) should be discussed more in depth.

8. The authors should have included the model ordinary differential equations and rate expressions for the revision of the manuscript.

Referee #2:

The authors describe the development and analysis of a large, new model of myocardial excitation-contraction-energetics coupling. Their analysis concludes that mitochondrial NADH generation is a key aspect of reperfusion injury. The results section contains useful discussion regarding model simulation results and how they align or do not align with our current understanding of the cardiomyocyte response to ischemia/reperfusion injury. They found that if NADH production was maintained during reperfusion, reperfusion injury can be mitigated. This finding has potential to be of profound interest; however, the result appears baked into the model. Specifically, on line 744, the authors state, "mitochondrial components were decreased to 70% as in the standard reperfusion protocol." The finding that undoing this decrease leads to higher NADH levels and better ATP production rates is a trivial result. Moreover, on line 420, authors state that OXPHOS recovery is only partial, but this is set by the user and not a simulated phenomenon. A more useful model would be able to simulate this phenomenon de novo; however, that may be beyond the scope of this study. Simulating quantitative data from cardiomyocyte hypoxia/reperfusion studies would significantly strengthen the impact of this model.

That said, the model qualitatively, and in some cases quantitatively, reproduce some of the major events that occur in an isolated cardiomyocyte during ischemia/reperfusion such as Na+ accumulation; however, it does not fully capture a few important physiological events such as accumulation of succinate, breakdown of purine nucleotides during ischemia, and RET mode of ROS production during reperfusion due to excess succinate.

The mitochondrial pH may be a bit high considering a large cytosolic K+ concentration. I am aware "consensus" values lie between 7.5-8; however, there is evidence that these consensus values are overestimates (see PMID: 27557123). In my opinion, lower matrix pH values make more sense considering 1) pmf = dPsi + dpH, 2) eukaryotic ATP synthase is more sensitive to dPsi vs chloroplast ATP synthase being more sensitive to dpH, and 3) nature is very good at optimization, so eukaryotic mitochondria would have high dPsi and low dpH to maximize ATP production rates. Regardless of my opinion, how would lowering the pH to values closer to cytosolic pH values influence the model outputs?

The +/- 10% perturbation for parameter sensitivity estimation is a bit too large for my comfort. Please use a smaller perturbation. Also, this appears not to be a standard method. It looks like a modified central difference method. Regardless, sensitivity coefficients should be normalized to enable a like-for-like comparison. Alternatively, the complex method is worth looking at considering its simplicity and better accuracy. See https://doi.org/10.1137/S003614459631241X.

In key points and abstract, it would be better to state why preserving NADH improves recovery (i.e., improved ATP production).

On line 278, Ca mito should go down considering mitos loaded with calcium during ischemia and reperfusion opening PTP would dump all calcium into cytosol. I'm comparing Fig 5F for cytosolic Ca and Fig A5B for matrix calcium. Also on this topic, 1 mM matrix Ca concentration is extremely large. I am unfamiliar with any empirical study supporting this model output considering the cytosolic Ca concentration given in Fig 5F. On lines 414-415, the authors state that "dPsi depolarizes due to the opening of mPTP triggered by the increase in Ca2+mit," so this should lead to equilibration between the cytosolic and matrix calcium concentrations. Am I missing something?

Em's are midpoint potentials, redox potential is ambiguous. As it is defined, Em's are Eh's or redox poise and Em0 is the midpoint potential.

Figure A10 shows what appears to be an unrealistically high O2.- concentration in the 100s of mM range.

END OF COMMENTS

岡山大学
OKAYAMA UNIVERSITY

National University Corporation OKAYAMA UNIVERSITY

1-1-1Tsushima-naka, Kita-ku, Okayama
700-8530 Japan
Tel. +81-86-251-7978

June 10th, 2026

Professor Natalia Trayanova
Senior Editor
The Journal of Physiology

Revision of JP-RP-2025-290440

Thank you for your positive reply. We have carefully studied the comments by Editors and Referees, and revised manuscript according to their suggestions. We replied point-by-point to the comments as coloured blue below. In the manuscript, we highlighted in blue any changes, including typos and errors.

Maor revision points are as follows.
1. The model and ischemia-reperfusion protocol were updated so that simulation results better fit existing experimental data of global ischemia of heart. We added new figure comparing simulation results with experimental data (new Figure 7).
2. ROS model was removed according to reviewers' criticism on it.
3. Physiologically new findings that were not "baked" into the model were added and explained carefully.
4. Figures that were included in Appendix have been moved to main text, and presentation of data has been reduced to the bare minimum.

Sincerely yours,
Ayako Takeuchi

REQUIRED ITEMS FOR REVISION

- Author photo and profile. First or joint first authors are asked to provide a short biography (no more than 100 words for one author or 150 words in total for joint first authors) and a portrait photograph. These should be uploaded and clearly labelled together in a Word document with the revised version of the manuscript. See Information for Authors for further details.

We uploaded short biographies and portrait photographs of the joint first authors.

- Please upload separate high-quality figure files via the submission form.

We uploaded high-quality figures with 600 dpi.

- Your paper contains Supporting Information of a type that we no longer publish, including supplementary tables and figures. Any information essential to an understanding of the paper must be included as part of the main manuscript and figures. The only Supporting Information that we publish are video and audio, 3D structures, program codes and large data files. Your revised paper will be returned to you if it does not adhere to our Supporting Information Guidelines.

Any information essential for understanding the paper is included in the main manuscript and figures. In addition, our "Online Supplementary Material" provides a complete set of model equations and parameters in table of 38 pages. We considered it is too extensive for inclusion as an Appendix. Although the source code is publicly available, we believe a full, text-based description of the equations will be useful for readers who are not familiar with programming. We have therefore submitted this material as Supporting Information. Having that said, it would be technically feasible to present it as an Appendix, and we are happy to follow the editor's preference.

- Please include an Abstract Figure file and an Abstract Figure legend. An appropriate figure legend, which should not exceed 150 words in length, should be included in the main manuscript file. The Abstract Figure is a piece of artwork designed to give readers an immediate understanding of the research and should summarise the main conclusions. If possible, the image should be easily 'readable' from left to right or top to bottom. It should show the physiological relevance of the manuscript so readers can assess the importance and content of its findings. Abstract Figures should not merely recapitulate other figures in the manuscript. Please try to keep the diagram as simple as possible and without superfluous information that may distract from the main conclusion(s). Abstract Figures must be provided by authors no later than the revised manuscript stage and should be uploaded as a separate file during online submission labelled as File Type 'Abstract Figure'. Please also ensure that you include the figure legend in the main article file. All Abstract Figures should be created using BioRender. Authors should use The Journal's premium BioRender account to export high-resolution images. Details on how to use and access the premium account are included as part of this email.

We created and uploaded an Abstract Figure. We also included the Abstract Figure legend in the main article file (page 32, lines 6-18).

- Please include a full title page as part of your main article (Word) file, which should contain the following: title, authors, affiliations, corresponding author name and contact details, keywords, and running title.

We included a full title page which contains all required information.

EDITOR COMMENTS

Reviewing Editor:

You paper has been reviewed by two experts in the field, who felt it is novel and addresses an important area, making it a potentially valuable scientific contribution. There were, however, significant limitations identified that must be addressed, as outlined in the Comments for the Author.

Thank you for your evaluation on our work and valuable feedback. Your specific concerns have been very helpful in revising this manuscript. We will respond point-by-point to your and the referees' comments as follows.

In particular, there were concerns related to: (i) how changes in oxidative phosphorylation were modelled;

Thank you for the comments. We employed our previously published guinea-pig ventricular myocyte model with excitation-contraction-energy metabolism coupling (Matsuoka et al., *Prog Biophys Mol Biol*, 2004; Kuzumoto et al., *Prog Biophys Mol Biol*, 2008). The oxidative phosphorylation module was updated based on Dzbek and Korzeniewski (*J Biol Chem*, 2008), which considers K^+ movements across inner membrane to calculate individual contribution of $\Delta\Psi$ and ΔpH to total proton motive force. The model was further extended by incorporating our previously developed model of mitochondrial Ca^{2+} and Na^+ movements (Saito et al., *J Physiol*, 2016). The scaling factors of individual components were adjusted to reproduce oxygen consumption rates reported for the guinea-pig heart. To improve clarity, we have expanded the explanation in the "Methods" section by adding two new subsections, "Modelling mitochondrial function" and "Integration of H^+ and ATP in mitochondria and cytosol" (page 9, lines 1-10 and page 10, line 4-page 11, line 2).

(ii) important physiological effects that were not captured by the model;

Thank you for the comments. In this revised version, we have thoroughly updated the model parameters and structure. In particular, equation for O_2 decline during ischemia was updated so that the time courses of phosphocreatine and NADH changes well fitted experimental data of global ischemia of hearts (Marban et al., *Circ Res*, 1990; Chang et al., *Am J Physiol Heart Circ Physiol,* 2001; Sakamoto et al., *Jpn J Pharmacol*, 2000; Wengrowski et al. *Am J Physiol Heart Circ Physiol,* 2014). In addition, we found an interesting study showing that the ATP_i dependence of contraction

shifts to higher ATP concentrations in the absence of phosphocreatine (Mekhfi et al., *Circ Res*, 1996), and incorporated this phenomenon into the model. The updated model quantitatively reproduces a wide range of independent datasets reported in various literature (see new Figure 7). However, the ROS production component could not be fully resolved within the current framework. In addition to predicting an extremely high concentration of ROS, which had been pointed out by both Referees, the model does not encompass the reverse electron flow driven by succinate accumulation during reperfusion. Therefore, we have decided to omit the ROS component from the revised model. We have added a description of this limitation in the "Discussion" section as follows.

Using the updated model, we found important physiological phenomena: 1) preserving NADH production mitigates the reperfusion injury even though other mitochondrial functions are hampered, 2) activation of NaK is effective for mitigating the reperfusion injury, and 3) short-term suppressions of ATP consumption by SERCA and contraction at the start of reperfusion is effective for mitigating the reperfusion injury. We carefully described these findings in the revised manuscript.

(ii) the timing of ischaemic effects and the magnitude of some physiological changes compared to experimental evidence;

Thank you for the valuable comment. In the update model, the simulated contraction ceased approximately 5 minutes earlier than the cessation of action potential (see new Figure 6), which is consistent with observations in rabbit and guinea-pig hearts (Shine et al., *Am J Physiol*, 1976; Shigematsu et al., *Circulation*, 1995). This earlier cessation is attributable to the above-mentioned shift in ATP_i dependence of contraction in the absence of phosphocreatine. The timing of ischemic effects and the magnitude of several physiological changes are largely comparable to various experimental datasets (see new Figure 7). However, several phenomena exhibited slight deviations from experimental data, such as a relatively sharper time course of ATP_i decline and smaller Pi increase. At present, we have no definitive explanation for this deviation; however, they might be due to the simplified metabolic pathways such as glycolysis, or the fact that this is a single-cell level rather than a tissue-level analysis. This leaves room for further refinement in future studies as discussed in the manuscript.

We have added the description in the "Results" section (page 15, lines 3-8). For the deviations, please refer to the "Discussion" section where they are already addressed (page 24, lines 5-13).

and (iv) what species was being modelled, which is important as effects of ischaemia vary between species.

Thank you for pointing this out. We employed our guinea-pig ventricular myocyte model (Matsuoka et al., *Prog Biophys Mol Biol*, 2004; Kuzumoto et al., *Prog Biophys Mol Biol*, 2008), in which most cellular components were formulated based on experimental data obtained from guinea-pigs. The mitochondrial module, however, represents a hybrid constructed from multiple species and was

modified to reproduce an oxygen consumption rate comparable to that of the guinea-pig heart (Kuzumoto et al., *Prog Biophys Mol Biol*, 2008). The newly incorporated pH regulation model (Crampin and Smith, *Biophys J*, 2006) is based primarily on experimental data from guinea-pigs, with additional data from sheep and rabbits. Since experimental data we refer to are basically obtained using larger mammals than rodents, we believe that our analysis addresses phenomena that are close to human than to rodent physiology.

To clarify these points for the readers, we specified the species to be referenced in the "Methods" section (page 6, line 18; page 6, line 23-page 7, line 1; page 7, line 10).

In addition, it was felt the paper would be stronger if novel hypotheses generated by the model for future experimental work were discussed,

Thank you for the valuable comment. Prompted by the Reviewing Editor's suggestion, we formulated a new working hypothesis. Based on the findings that rescue of NADH production restores ATP_i level and thereby mitigating reperfusion-induced injury in the model cell (old Figures 5, 6), we hypothesized that saving ATP consumption during reperfusion period would be beneficial. To test this idea, we individually inhibited four ATP-consuming processes, i.e., Na^+/K^+ pump, contraction and SERCA, during initial 5 min of reperfusion period and evaluated their effects on reperfusion injury in the model. Among these interventions, suppression of contraction partially and that of SERCA fully restored contractile function at the end of reperfusion (see new Figure 15).

To date, studies on the effects of manipulating SERCA activity on the reperfusion injury have yielded conflicting results. SERCA inhibition in the early stage of reperfusion was suggested to diminish the cell damage by preventing mPTP opening (Abdallah et al., *J Cell Mol Med*, 2011), while SERCA1a over-expression was reported to be beneficial against the reperfusion injury (Talukder et al., *Am J Physiol Heart Circ Physiol*, 2007). Recently, exogenous application of a micropeptide that inhibits SERCA, myoregulin, was reported to attenuate functional impairment caused by ischemia-reperfusion (Appleby et al., *Peptides*, 2024). Given these conflicting experimental data, our computational model provides a mechanistic basis that strongly supports the beneficial role of SERCA regulation.

The strategy to attenuate reperfusion injury by suppressing contraction using 2,3-butanedione monoxime (BDM) is classical and has been explored for decades; however, its efficacy remains debated, probably due to its non-selective and protocol-dependent effects (Armstrong and Ganote, *J Mol Cell Cardiol*, 1991; Habazettl et al., *Cardiovasc Res*, 1998). In the last few years, two cardiac myosin-targeting agents, mavacamten and aficamten, were been approved by FDA for treating hypertrophic cardiomyopathy. Very recently, potential efficacy of these drugs on ischemia-reperfusion injury (Yusof et al., 2026). Our result theoretically supports this promising experimental result.

Activation of NaK to relive Na^+ overload during reperfusion was also found to be effective. So far, no clear experimental evidence was found on the protective effect of NaK activation. Zhang et al. reported that a NaK activating antibody, DRRSAb, protected hearts against ischemic injury in both cardiomyocytes and isolated hearts (Zhang et al., *Signal Transduct Target Ther*, 2024). However, this antibody has a positive inotropic effect and stimulation of Src/PI3K/Akt/ERK1/2 pathways were proposed for the underlying mechanism. Further studies are needed to examine the protective role of NaK activation on reperfusion injury.

Collectively, out simulation analysis proposes that short-term suppression of SERCA or contraction, or activation of NaK upon reperfusion are promising strategies for mitigating reperfusion injury.

We have added the description in the "Discussion" section (page 22, line 12-page 23, line 6).

along with a need for further clarification of some aspects of the study and other minor suggestions. If the authors feel they can adequately address the reviewers concerns they are encouraged to submit a revised version of the paper, along with a point-by-point response.

Thank you for the positive encouragement. We have addressed the Referees' concerns point-by-point and have revised the manuscript accordingly. We hope that the revised version satisfactorily addresses all comments and will be suitable for publication.

REFEREE COMMENTS

Referee #1:

The computational model presented in the manuscript of Shimayoshi et al. addresses an important challenge that has not been dealt with this type of modeling. Namely the simulation of the electrophysiological, mechanical and metabolic changes occurring during episodes of ischemia followed by reperfusion. The novelty in this modeling instance is given by the pH dependence of various transport processes across the plasma membrane together with a description encompassing mitochondrial energetics. The incorporation of the dynamics of the ROS that are believed to play an important role in the ischemia reperfusion injury is also novel. Previous attempts have failed to incorporate the dynamics of energy supply by mitochondrial activity. I would commend the authors for undertaking this modelling problem.

I have several suggestions to improve the modeling and the presentation thereof:

Thank you for your high evaluation on our work and valuable feedback. We have revised the manuscript according to the comments.

1. Regarding the modeling, it is never mentioned, which is the cardiac system that the authors are modeling. This is important because there are key differences between rodents E-C Coupling and other mammals (e.g. canine, pig, etc). The percentage of the Ca2+ recycled in each beat is completely different and the contrast with an experimental model result may be largely dependent on such Ca2+ dynamical behavior.

Thank you for pointing this out. We employed our guinea-pig ventricular myocyte model (Matsuoka et al., *Prog Biophys Mol Biol*, 2004; Kuzumoto et al., *Prog Biophys Mol Biol*, 2008), in which most cellular components were formulated based on experimental data obtained from guinea-pigs. The mitochondrial module, however, represents a hybrid constructed from multiple species and was modified to reproduce an oxygen consumption rate comparable to that of the guinea-pig heart (Kuzumoto et al., *Prog Biophys Mol Biol*, 2008). The newly incorporated pH regulation model (Crampin and Smith, *Biophys J*, 2006) is based primarily on experimental data from guinea-pigs, with additional data from sheep and rabbits. Since experimental data we refer to are basically obtained using larger mammals than rodents, we believe that our analysis addresses phenomena that are close to human than to rodent physiology.

2. The dynamics of contractile activity decline during ischemia (total flow ischemia) in isolated hearts is much faster than 20 minutes (see <2 min in Lyon ... and Akar, 2010, PMID: 20624394; or Laclau et al. 2001 PMID: 11343417 reporting a contracture of the heart after 10 min ischemia) although the action potential decrease in amplitude is slower.

Thank you for pointing this out. Prompted by this valuable comment, we found an interesting study showing that the ATP_i dependence of contraction shifts to higher ATP concentrations in the absence of phosphocreatine (Mekhfi et al., *Circ Res*, 1996), and incorporated this phenomenon into the model. This enabled the model to reproduce the earlier cessation of contraction relative to the action potential, and we greatly appreciate the Referee #1's insight.

In addition to the above improvement, we have thoroughly updated the model parameters and structure. In particular, the equation for O_2 decline during ischemia was updated so that the time courses of phosphocreatine and NADH changes well fit the experimental data of g of global ischemia of hearts (Marban et al., *Circ Res*, 1990; Chang et al., *Am J Physiol Heart Circ Physiol,* 2001; Sakamoto et al., *Jpn J Pharmacol*, 2000; Wengrowski et al. *Am J Physiol Heart Circ Physiol,* 2014).

The updated model quantitatively reproduces a wide range of independent datasets reported in various literature (see new Figure 7). Notably, the simulated contraction ceased approximately 5 minutes earlier than the cessation of action potential (see new Figure 6), which is consistent with observations in rabbit and guinea-pig hearts (Shine et al., *Am J Physiol*, 1976; Shigematsu et al., *Circulation*, 1995). The experimentally observed faster time courses (<2 min), suggested by Referee #1, might be due to species differences, such as between rats and guinea-pig/ferrets.

We have added the new subsection "ATP and PCr dependence of contraction" in the "Methods" section (page 9, lines 11-19), and the description in the "Results" section (page 15, lines 3-8).

Concerning the appearance of metabolic changes, Gudbjarnason et al, (1970) PMID: 5519941 reported that CrP falls after only 1 minute of ischemia whereas ATP remained constant in that temporal window. In this

reviewer's opinion, there may be a lack of feedback between the Pi released upon ischemia on mitochondrial ATP synthase, or an incorrect parametrization of the creatine kinase activity, but the authors should find why ATP continues to be synthesized when there should be no inner mitochondrial membrane potential as demonstrated in Lyon et al. 2010. This is a serious shortcoming of the model and should be clarified.

Thank you for the insightful comment. In both the previous and updated models, phosphocreatine levels begin to decline immediately upon the onset of oxygen deprivation, and once phosphocreatine is completely depleted, ATP_i start to decrease (see old Figure 2I and new Figure 6I). These metabolic alterations are qualitatively similar to those observed in myocardial tissue from coronary artery-ligated canine hearts (Gudbjarnason et al., *J Mol Cell Cardiol*, 1970) and Langendorff-perfused rat hearts (Kay et al., *J Mol Cell Cardiol*, 1997). As noted above, we have updated the model so that the time course of phosphocreatine decrease well fits the experimental data of global ischemia of hearts (Marban et al., *Circ Res*, 1990; Chang et al., *Am J Physiol Heart Circ Physiol,* 2001; Sakamoto et al., *Jpn J Pharmacol*, 2000).

In our model, ATP synthase flux (v_{SN}) is Pi-dependent, as described by the equations below;

$$v_{SN} = SF_{mit} \cdot k_{SN} \cdot \frac{\gamma_{SN} - 1}{\gamma_{SN} + 1}$$

where $\gamma_{SN} = e^{\left(n_{HATP} \cdot \frac{F \cdot \Delta p}{R \cdot T} - \left(\frac{\Delta G_{SN} \cdot 1000}{R \cdot T} + \ln\left(\frac{[ATP_{tot}]_{mit} \cdot 1000}{[ADP_{tot}]_{mit} \cdot [PI_{tot}]_{mit}}\right)\right)\right)}$

this formulation accounts for the feedback regulation of ATP synthase by Pi. This regulation, together with the creatine kinase flux, helps maintain the ATP_i level for a certain period. Regarding the mitochondrial energetics, it should be noted that $\Delta\Psi$ remains partially preserved (depolarized but not lost) at the end of ischemia (old Figure 2L and new Figure 6L), thereby sustaining a minor, residual ATP_i supply. In Langendorff-perfused rat hearts (Lyon et al., *J Mol Cell Cardiol*, 2010), $\Delta\Psi$ returned to baseline upon reperfusion after short-duration (7.5 min) global ischemia, whereas it underwent further depolarization following a longer ischemic insult (15 min). Our model exhibits a hybrid response; i.e., $\Delta\Psi$ partially recovers at the onset of reperfusion, but then re-depolarizes (old Figure 2L and new Figure 6L). Because Lyon et al. (*J Mol Cell Cardiol*, 2010) did not assess ATP_i, the relationship between $\Delta\Psi$ and ATP_i supply cannot be directly evaluated from their experimental data. Please see new Figure 7I, there are large variation in ATP level and at least several % of ATP remains at 15 min of global ischemia. We speculate that capacity of mitochondrial ATP synthesis during ischemia largely depends on species and experimental conditions. Nevertheless, our model analyses showed certain limitations, such as a relatively sharper time course of ATP_i decline and smaller Pi increase. At present, we have no definitive explanation for this deviation; however, they might be due to the simplified metabolic pathways such as glycolysis, or the fact that this is a single-cell level rather than a tissue-level analysis. This leaves room for further refinement in future studies as discussed in the manuscript (page 24, lines 5-13)

3. This reviewer is interested to know about the quantitative comparison of the burst in cytoplasmic superoxide production at the onset of reperfusion with respect to experimental models of IR injury, since, even if transient, 200mM superoxide sounds awfully high for cardiomyocytes function.

Thank you for the critical comments. We agree with Referee #1 that cytosolic ROS concentration of 200 mM is unrealistically high. The ROS metabolism components are derived from Kembro et al. (*Biophys J*, 2013), which was well-validated using isolated mitochondria. However, we found that quantitative description of ROS dynamics by integrating the components into our excitation-contraction-energy metabolism coupling, however, was challenging. Furthermore, the model does not encompass the reverse electron transport driven by succinate accumulation during early reperfusion. Therefore, we have decided to omit the ROS component from the revised model. This allows the model to focus robustly on its primary strengths: the coupling of excitation, contraction, and core energy metabolism. We believe this omission significantly improves the reliability and clarity of the manuscript.

4. Despite the still unresolved issues of the redox role in IR injury and the molecular identity of the mPTP, a model can postulate hypotheses that may contribute to clarifying the controversies and suggest experiments to solve the open questions. I believe that at least this should be discussed as a follow up to these modeling studies.

Thank you for the comment. As Referee #1 notes, the redox role in ischemia-reperfusion and molecular identity of mPTP remain unsolved questions. We acknowledge that these are important and attractive challenges; however, they are beyond the scope of the current study.

Minor issues

5. A glossary of symbols and abbreviations should be included to ease understanding of the model variables and parameters.

Thank you for the comment. We have newly created an “Abbreviations” section.

6. Lines 190-191. The authors should include references to justify those arguments.

Thank you for the comment. The concept that mPTP is closed under physiological condition is widely accepted, as noted in early review (e.g., Halestrap, *J Mol Cell Cardiol*, 2009). Although transient, stochastic opening of mPTP has been reported in several studies (e.g., Wang et al., *Cell*, 2008; Lu et al., *Circ Res*, 2015), Boyden et al. (*Arch Biochem Biophys*, 2019) presented evidence that mPTP opening events are extremely rare under quiescent conditions in cardiomyocytes. We have therefore included the citations (Halestrap, *J Mol Cell Cardiol*, 2009; Boyman et al., *Arch Biochem Biophys*, 2019) in the revised manuscript.

7. Related to my point #4 the section on redox changes during and after ischemia and their dynamics (as in appendix Figure A6) should be discussed more in depth.

Thank you for the valuable comment. Although evaluating the effect of ischemic period on redox changes would give us insightful results, as noted in the response #3, we have decided to omit the ROS model from the current integrated model. Therefore, we would like to leave this issue for future modelling studies.

8. The authors should have included the model ordinary differential equations and rate expressions for the revision of the manuscript.

Thank you for the comment. In the revised manuscript, we have included ordinary differential equations which are important for this study (integration of cytosolic and mitochondrial ATP and H^+) in the "Methods" section. In addition, we included all model ordinary differential equations and rate expressions in the table of 38 pages long "Online Supplementary Material", which we considered too extensive to be placed in the main manuscript. While it would be technically feasible to present them as an Appendix, we are happy to follow the editor's preference.

Referee #2:

The authors describe the development and analysis of a large, new model of myocardial excitation-contraction-energetics coupling. Their analysis concludes that mitochondrial NADH generation is a key aspect of reperfusion injury. The results section contains useful discussion regarding model simulation results and how they align or do not align with our current understanding of the cardiomyocyte response to ischemia/reperfusion injury.

Thank you for your evaluation on our work and valuable feedback. We have revised the manuscript according to the comments.

They found that if NADH production was maintained during reperfusion, reperfusion injury can be mitigated. This finding has potential to be of profound interest; however, the result appears baked into the model. Specifically, on line 744, the authors state, "mitochondrial components were decreased to 70% as in the standard reperfusion protocol." The finding that undoing this decrease leads to higher NADH levels and better ATP production rates is a trivial result.

Thank you for the comment. To be more consistent with experimental data, we assumed that mitochondrial activities are reduced to 50%, instead of 70%, at the onset of reperfusion in the revised model, based on the observations of Kuzmiak-Glancy et al. (*Am J Physiol Heart Circ Physiol*, 2022). Importantly, our results do not simply reflect the reversal of this global reduction. Even under this impaired mitochondrial state, restoration of dehydrogenase activity alone was sufficient to attenuate reperfusion injury (old Figure 5 and new Figure 12). Conversely, when only dehydrogenase activity was impaired, reperfusion injury still occurred despite normal activities of all

other mitochondrial components. Taken together, these findings indicate that NADH production by dehydrogenases is a key determinant for mitigating reperfusion injury, rather than a trivial consequence of undoing a uniform 50% reduction ("baked-in" result).

Moreover, on line 420, authors state that OXPHOS recovery is only partial, but this is set by the user and not a simulated phenomenon. A more useful model would be able to simulate this phenomenon de novo; however, that may be beyond the scope of this study.

Thank you for the comment. Our previous explanation was misleading and did not adequately capture the phenomenon. Our intent was to convey that, upon reperfusion, OXPHOS resumes and supports ATP synthesis; nevertheless, the incomplete recovery of dehydrogenase activity limits NADH supply and thereby prevents the generation of sufficient ATP. Indeed, even when all other mitochondrial components are allowed to fully recover while dehydrogenases remain impaired, the simulation still exhibits reperfusion injury (old Figure 5 and new Figure 12). These results indicate that the observed partial recovery of OXPHOS arises from the simulated interplay among dehydrogenase-driven NADH production, ΔΨ, and adenine nucleotide dynamics, rather than from predetermined parameter choices. We apologize for the confusing statements and have thoroughly revised the text to explain in more detail the ischemia-reperfusion injury in the "Discussion" section (page 21, lines 4-23).

Simulating quantitative data from cardiomyocyte hypoxia/reperfusion studies would significantly strengthen the impact of this model. That said, the model qualitatively, and in some cases quantitatively, reproduce some of the major events that occur in an isolated cardiomyocyte during ischemia/reperfusion such as Na+ accumulation;

Thank you for the comments. In this revised version, we have thoroughly updated the model parameters and structure. In particular, equation for O_2 decline during ischemia was updated so that the time courses of phosphocreatine and NADH changes well fitted experimental data of global ischemia of hearts (Marban et al., *Circ Res*, 1990; Chang et al., *Am J Physiol Heart Circ Physiol,* 2001; Sakamoto et al., *Jpn J Pharmacol*, 2000; Wengrowski et al. *Am J Physiol Heart Circ Physiol,* 2014). In addition, we found an interesting study showing that the ATP_i dependence of contraction shifts to higher ATP concentrations in the absence of phosphocreatine (Mekhfi et al., *Circ Res*, 1996), and incorporated this phenomenon into the model. The updated model quantitatively reproduces a wide range of independent datasets reported in various literature (see new Figure 7). We have added the new subsection "ATP and PCr dependence of contraction" in the "Methods" section (page 9, lines 11-19), and the description on the extensive comparison with experimental data in the "Results" section (page 14, line 16-page 15, line 8).

however, it does not fully capture a few important physiological events such as accumulation of succinate, breakdown of purine nucleotides during ischemia, and RET mode of ROS production during reperfusion due to excess succinate.

Thank you for pointing this out. In addition to predicting an extremely high concentration of ROS, as we will reply to the following question, the model does not encompass the reverse electron flow driven by succinate accumulation during reperfusion. Therefore, we have decided to omit the ROS component from the revised model. This allows the model to focus robustly on its primary strengths: the coupling of excitation, contraction, and core energy metabolism. We believe this omission significantly improves the reliability and clarity of the manuscript.

The mitochondrial pH may be a bit high considering a large cytosolic K+ concentration. I am aware "consensus" values lie between 7.5-8; however, there is evidence that these consensus values are overestimates (see PMID: 27557123). In my opinion, lower matrix pH values make more sense considering 1) pmf = dPsi + dpH, 2) eukaryotic ATP synthase is more sensitive to dPsi vs chloroplast ATP synthase being more sensitive to dpH, and 3) nature is very good at optimization, so eukaryotic mitochondria would have high dPsi and low dpH to maximize ATP production rates. Regardless of my opinion, how would lowering the pH to values closer to cytosolic pH values influence the model outputs?

Thank you for the comment. The paper showing that the matrix pH in the yeast is much lower than had been generally assumed is very interesting, but we consider that verification using mammalian heart mitochondria is needed. Nevertheless, we performed ischemia/reperfusion simulation with lowering the pH to 7.20, which are close to cytosolic pH, by increasing H^+ production by substrate dehydrogenation by about 1%. As presented below for the review reference only and not included in the manuscript, the responses of the model cell to ischemia/reperfusion were almost the same when lowering pH_{mit}, with the trajectories of $\Delta\Psi$ and dpH changes merely exhibiting a parallel shift, except for a negligible reduction in Ca^{2+}_i overload and cell shortening (pH_{mit} = 7.67 (black lines) and pH_{mit} = 7.2 (blue lines)). Our opinion, however, is that these small effects should not contribute to any qualitative change in the simulated pathophysiology of ischemia/reperfusion.

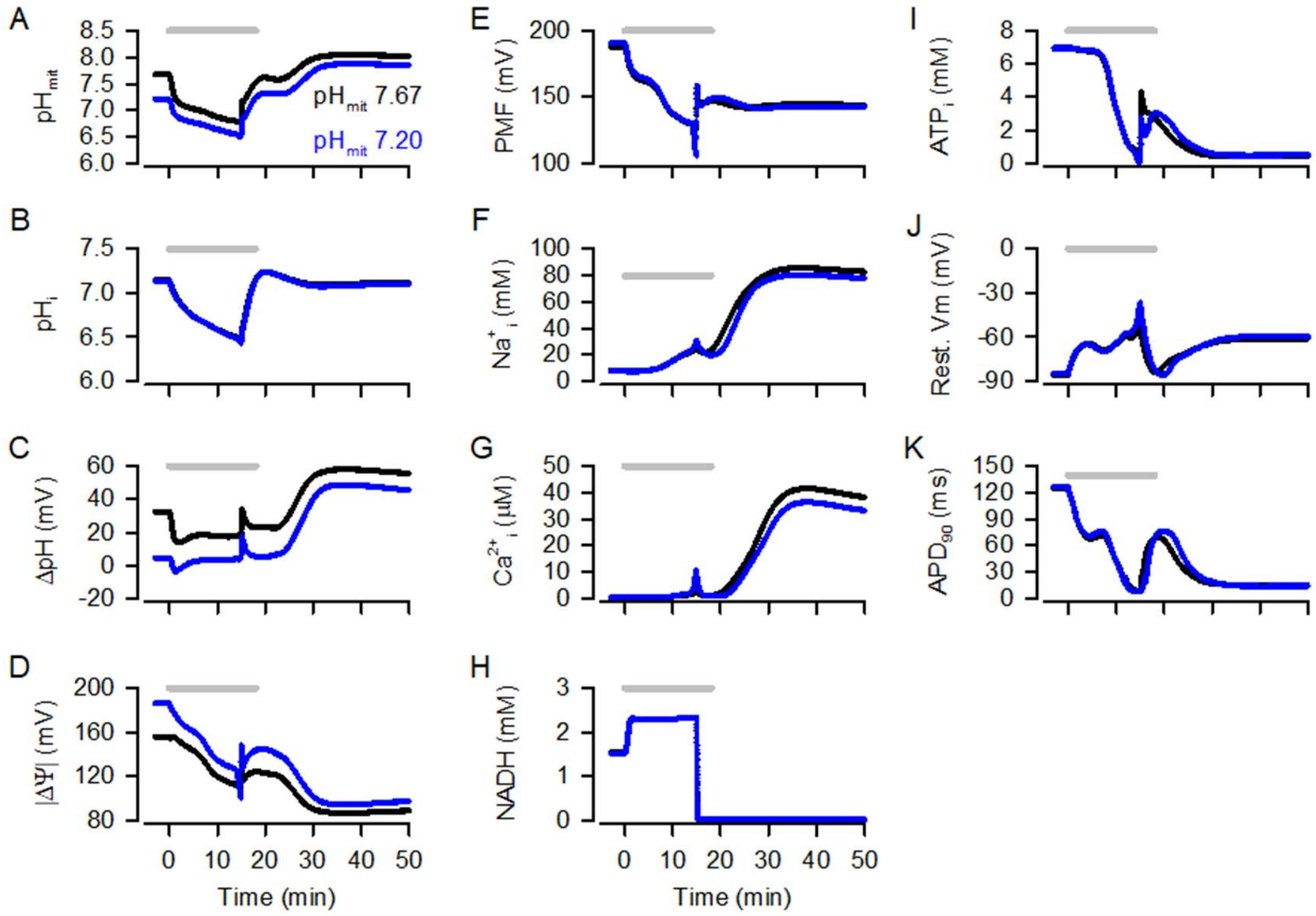

Figure only for review. Effects of lowering pH_{mit} on the responses to ischemia and reperfusion. The black and blue lines show the simulated results of ischemia-reperfusion with original pH_{mit} (7.67) and lowered pH_{mit} (7.20), respectively. A. pH_{mit}, B. pH_i, C. ΔpH, D. |ΔΨ|, E. PMF, F. Na^+_i, G. Ca^{2+}_i, H. NADH, I. ATP_i, J. resting V_m, K. APD_{90}.

The +/- 10% perturbation for parameter sensitivity estimation is a bit too large for my comfort. Please use a smaller perturbation. Also, this appears not to be a standard method. It looks like a modified central difference method. Regardless, sensitivity coefficients should be normalized to enable a like-for-like comparison. Alternatively, the complex method is worth looking at considering its simplicity and better accuracy. See https://doi.org/10.1137/S003614459631241X.

Thank you for pointing this out. We re-performed sensitivity analyses by applying +/-1, 2, and 0% perturbation of each parameter (new Figure 11), then calculated sensitivity values using linear regression. The results are essentially the same as those in the original manuscript, though the model was updated as described above and way of sensitivity analysis was changed.

In key points and abstract, it would be better to state why preserving NADH improves recovery (i.e., improved ATP production).

Thank you for the comments. We updated the “Key points summary” and “Abstract”.

On line 278, Ca mito should go down considering mitos loaded with calcium during ischemia and reperfusion opening PTP would dump all calcium into cytosol. I'm comparing Fig 5F for cytosolic Ca and Fig A5B for matrix calcium. Also on this topic, 1 mM matrix Ca concentration is extremely large. I am unfamiliar with any empirical study supporting this model output considering the cytosolic Ca concentration given in Fig 5F. On lines 414-415, the authors state that "dPsi depolarizes due to the opening of mPTP triggered by the increase in Ca2+mit," so this should lead to equilibration between the cytosolic and matrix calcium concentrations. Am I missing something?

Thank you for the comments. During the ischemia–reperfusion period, the $\Delta\Psi$ remains partially preserved (depolarized but not abolished), which maintains a residual electrochemical driving force for Ca^{2+} influx from the cytosol to the matrix. The cytosolic Ca^{2+} elevation is driven primarily by sarcoplasmic reticulum Ca^{2+} unloading and plasma membrane Ca^{2+} entry, because ATP_i depletion impairs SERCA, Na^+/K^+ pump and thereby Na^+-Ca^{2+} exchanger. Because Ca^{2+} is continuously supplied from outside the cells, matrix Ca^{2+} do not go down even when mPTP is open. Because absolute concentration measurements are highly sensitive to calibration procedures and experimental conditions, the precise magnitude cannot be stated with confidence.

Em's are midpoint potentials, redox potential is ambiguous. As it is defined, Em's are Eh's or redox poise and Em0 is the midpoint potential.

Thank you for pointing this out. However, for simplicity and to maintain consistency with the original work of Dzbek and Korzeniewski (*J Biol Chem*, 2008), we have kept the parameter explanations unchanged.

Figure A10 shows what appears to be an unrealistically high O2.- concentration in the 100s of mM range.

Thank you for the critical comments. As mentioned above, we found that quantitative description of ROS dynamics by integrating the components into our excitation-contraction-energy metabolism coupling, is challenging. Therefore, we have decided to omit the ROS component from the revised model.

1st Revision - Editorial Decision 24-Jun-2026

Dear Dr Takeuchi,

Re: JP-RP-2026-290440R1 "Integrative simulation analysis of myocardial ischemia-reperfusion injury" by Takao Shimayoshi, Ayako Takeuchi, and Satoshi Matsuoka

Thank you for submitting your manuscript to The Journal of Physiology. It has been assessed by a Reviewing Editor and by 2 expert referees and we are pleased to tell you that it is acceptable for publication following satisfactory revision.

The referee reports are copied at the end of this email.

Please address all the points raised and incorporate all requested revisions or explain in your Response to Referees why a change has not been made. We hope you will find the comments helpful and that you will be able to return your revised manuscript within 4 weeks. If you require longer than this, please contact journal staff: jp@physoc.org.

Your revised manuscript should be submitted online using the link in your Author Tasks Link Not Available. This link is accessible via your account as Corresponding Author; it is not available to your co-authors. If this presents a problem, please contact journal staff (jp@physoc.org). Image files from the previous version are retained on the system. Please ensure you replace or remove any files that are being revised.

If you do not wish to submit a revised version of your manuscript, you must inform our journal staff (jp@physoc.org) or reply to this email to request withdrawal. Please note that a manuscript must be formally withdrawn from the peer review process at one journal before it may be submitted to another journal.

TRANSPARENT PEER REVIEW POLICY: To improve the transparency of its peer review process The Journal of Physiology publishes online, as supporting information, the peer review history of all articles accepted for publication. Readers will have access to decision letters, including Editors' comments and referee reports, for each version of the manuscript, as well as any author responses to peer review comments. Referees can decide whether or not they wish to be named on the peer review history document.

ABSTRACT FIGURES: Authors are expected to use The Journal's premium BioRender account to create/redraw their Abstract Figures. Information on how to access this account is here: https://physoc.onlinelibrary.wiley.com/journal/14697793/biorender-access.

This will enable Authors to create and download high-resolution figures. If authors have used the free BioRender service, they can use the instructions provided in the link above to download a high-resolution version suitable for publication.

The link provided should only be used for the purposes of this submission. Authors will be charged for figures created on this account if they are not related to this manuscript submission.

LANGUAGE EDITING AND SUPPORT FOR PUBLICATION: If you would like help with English language editing, or other article preparation support, Wiley Editing Services offers expert help, including English Language Editing, as well as translation, manuscript formatting, and figure formatting at www.wileyauthors.com/eeo/preparation. You can also find resources for Preparing Your Article for general guidance about writing and preparing your manuscript at www.wileyauthors.com/eeo/prepresources.

RIGOUR AND REPRODUCIBILITY: We recommend authors consult The Journal's Rigour and Reproducibility requirements to ensure that their manuscript is suitable for publication: https://physoc.onlinelibrary.wiley.com/pb-assets/hub-assets/physoc/documents/TJP-Rigour-and-Reproducibility-Requirements.pdf

REVISION CHECKLIST:

Check that your Methods section conforms to journal policy: https://jp.msubmit.net/cgi-bin/main.plex?form_type=display_requirements#methods

Check that data presented conforms to the statistics policy: https://jp.msubmit.net/cgi-bin/main.plex?form_type=display_requirements#statistics

Upload a full Response to Referees file. To create your 'Response to Referees' copy all the reports, including any comments from the Senior and Reviewing Editors, into a Microsoft Word, or similar, file and respond to each point, using font or background colour to distinguish comments and responses and upload as the required file type.

Please upload two versions of your manuscript text: one with all relevant changes highlighted and one clean version with no changes tracked. The manuscript file should include all tables and figure legends, but each figure/graph should be uploaded as separate, high-resolution files. The journal is now integrated with Wiley's Image Checking service. For further details, see: https://www.wiley.com/en-us/network/publishing/research-publishing/trending-stories/upholding-image-integrity-wileys-image-screening-service

You may also upload:

- 'Potential Cover Art' for consideration as the issue's cover image
- Appropriate Supporting Information (Video, audio or data set: see https://jp.msubmit.net/cgi-bin/main.plex?form_type=display_requirements#supp).

We look forward to receiving your revised submission.

If you have any queries, please reply to this email and we will be pleased to advise.

Yours sincerely,

Natalia Trayanova
Senior Editor
The Journal of Physiology

REQUIRED ITEMS

- Authors are required to fully disclose the use of any Generative AI (GAI) tools used in the preparation of their manuscript. This disclosure should also be included as a statement alongside the Additional Information and Data Availability statements towards the end of the article under the heading 'Generative AI Statement', and include the name(s), model and version of AI technology used, and exact details on the purpose for, and methods of, its use.

If no AI tool has been used, this should also be specifically stated within the manuscript.

EDITOR COMMENTS

Reviewing Editor:

Comments to the Author:
The authors have satisfied the reviewers' concerns. There remain two final minor points for them to address, as outlined in the Referee Comments.

REFEREE COMMENTS

Referee #1:

The authors have addressed most of the issues raised by this reviewer. Despite being somewhat disappointed with the elimination of the ROS components from the revised manuscript, I believe the manuscript is largely improved and the authors have thoroughly dealt with the issues regarding the time dependence of CrP and ATP during and after ischemia-reperfusion injury.

The only observation is the confusing use of pHFactor, pHFacor and pHFactor2 (that I couldn't find). The authors should consolidate and unify the notation to clarify the names used throughout the manuscript.

Referee #2:

Authors satisfactorily addressed my comments.

Minor: Add a space between 3 and min on page 13. Use of "minutes" and "min" is inconsistent, e.g. see top of page 15, middle of 20, bottom of 31.

END OF COMMENTS

National University Corporation OKAYAMA UNIVERSITY

1-1-1Tsushima-naka, Kita-ku, Okayama
700-8530 Japan
Tel. +81-86-251-7978

June 26th, 2026

Professor Natalia Trayanova
Senior Editor
The Journal of Physiology

Revision of JP-RP-2026-290440R1

Thank you for your positive reply. We have carefully studied the comments by Editors and Referees, and revised manuscript according to their suggestions. We replied point-by-point to the comments as coloured blue below. In the manuscript, we highlighted in blue any changes, including typos and errors. In addition, we updated our “Online Supplementary Material”, to correct typos.

Sincerely yours,
Ayako Takeuchi

REQUIRED ITEMS

- Authors are required to https://jp.msubmit.net/cgi-bin/main.plex?form_type=display_requirements#aigc fully disclose the use of any Generative AI (GAI) tools used in the preparation of their manuscript. This disclosure should also be included as a statement alongside the Additional Information and Data Availability statements towards the end of the article under the heading 'Generative AI Statement', and include the name(s), model and version of AI technology used, and exact details on the purpose for, and methods of, its use.

If no AI tool has been used, this should also be specifically stated within the manuscript.

We have added a “Generative AI statement” under the “Additional information” section, clarifying that no generative AI tools were used in the preparation of this manuscript (page 25, lines 1-2).

EDITOR COMMENTS

Reviewing Editor:

Comments to the Author:

The authors have satisfied the reviewers' concerns. There remain two final minor points for them to address, as outlined in the Referee Comments.

Thank you for your evaluation of our work. We have revised the manuscript according to the referees’ suggestions. Our point-by-point responses are highlighted in blue below. In addition, we made the source code available on GitHub, and included the statement in the manuscript as follows;

“The source code is available on GitHub at https://github.com/atakeuti/TS-AT-MS-2026-IRmodel” (page 7, line 4; and page 24, lines 22-23).

REFEREE COMMENTS

Referee #1:

The authors have addressed most of the issues raised by this reviewer. Despite being somewhat disappointed with the elimination of the ROS components from the revised manuscript, I believe the manuscript is largely improved and the authors have thoroughly dealt with the issues regarding the time dependence of CrP and ATP during and after ischemia-reperfusion injury.

Thank you for your evaluation of our work. We appreciate your understanding regarding the elimination of the ROS components, which allowed us to focus on the primary strengths: the coupling of excitation, contraction, and core energy metabolism. We hope to explore the ROS components in greater depth in our future studies.

The only observation is the confusing use of pHFactor, pHFacor and pHFactor2 (that I couldn't find). The authors should consolidate and unify the notation to clarify the names used throughout the manuscript.

Thank you for pointing this out. The terms “pHFacor”, “pHFacor1” and “pHFacor2” in the equations were misspelled. We have corrected them to “pHFactor”, “pHFactor1” and “pHFactor2” (page 7, lines 20 & 22; page 8, lines 1 & 17 & 18).

Referee #2:

Authors satisfactorily addressed my comments.

Thank you for your evaluation of our work.

Minor: Add a space between 3 and min on page 13. Use of "minutes" and "min" is inconsistent, e.g. see top of page 15, middle of 20, bottom of 31.

Thank you for pointing them out. We have fixed the errors (page 13, line 19; page 15, line 6; page 20, line 13; page 31, line 23).

Dear Professor Takeuchi,

Re: JP-RP-2026-290440R2 "Integrative simulation analysis of myocardial ischemia-reperfusion injury" by Takao Shimayoshi, Ayako Takeuchi, and Satoshi Matsuoka

We are pleased to tell you that your paper has been accepted for publication in The Journal of Physiology.
- Authors are required to fully disclose the use of any Generative AI (GAI) tools used in the preparation of their manuscript. This disclosure should also be included as a statement alongside the Additional Information and Data Availability statements towards the end of the article under the heading 'Generative AI Statement', and include the name(s), model and version of AI technology used, and exact details on the purpose for, and methods of, its use.

If no AI tool has been used, this should also be specifically stated within the manuscript.

The last Word (or similar) version of the manuscript provided will be used by the Production Editor to prepare your proof. When this is ready you will receive an email containing a link to Wiley's Online Proofing System. The proof should be thoroughly checked and corrected as promptly as possible.

Authors should note that it is too late at this point to offer corrections prior to proofreading. Major corrections at proof stage, such as changes to figures, will be referred to the Editors for approval before they can be incorporated. Only minor changes, such as to style and consistency, should be made at proof stage. Changes that need to be made after proof stage will usually require a formal correction notice.

All queries at proof stage should be sent to: TJP@wiley.com.

Following copyediting and typesetting the accepted version will be published online.

Yours sincerely,

Natalia Trayanova
Senior Editor
The Journal of Physiology

IMPORTANT POINTS TO NOTE FOLLOWING ACCEPTANCE OF YOUR PAPER:

• IMPORTANT NOTICE ABOUT OPEN ACCESS: To assist authors whose funding agencies mandate immediate public access to published research findings, The Journal of Physiology allows authors to pay an Open Access (OA) fee to have their papers made freely available immediately on publication.

The Corresponding Author will receive an email from Wiley with details on how to register or log in to Wiley Authors where you will be able to place an order.

• You can check if your funder or institution has a Wiley Open Access Account here:
https://authors.wiley.com/author-resources/Journal-Authors/open-access/author-compliance-tool.html

MEMBER DISCOUNT: Corresponding authors who are Members of the Physiological Society are eligible for a 20% discount on the cost of the Article Processing Charge (APC). To claim this discount, the Corresponding Author will need to log in to the Physiological Society Membership portal and retrieve the discount code (https://www.physoc.org/journals-and-media/journals/journal-discount-for-members/). This code should then be quoted in the billing section of Wiley Authors when selecting the open access option.

• TRANSPARENT PEER REVIEW POLICY: To improve the transparency of its peer review process, The Journal of Physiology publishes online as supporting information the peer review history of all articles accepted for publication. Readers will have access to decision letters, including Editors' comments and referee reports, for each version of the manuscript as well as any author responses to peer review comments. Referees can decide whether or not they wish to be named on the peer review history document.

• You can help your research get the attention it deserves! Check out Wiley's free Promotion Guide for best-practice recommendations for promoting your work at: www.wileyauthors.com/eeo/guide. You can learn more about Wiley Editing Services which offers professional video, design, and writing services to create shareable video abstracts, infographics, conference posters, lay summaries, and research news stories for your research at: www.wileyauthors.com/eeo/promotion.

• If you would like to receive our 'Research Roundup', a monthly newsletter highlighting the cutting-edge research published

in The Physiological Society's family of journals (The Journal of Physiology, Experimental Physiology, Physiological Reports, The Journal of Nutritional Physiology and The Journal of Precision Medicine: Health and Disease), please click this link, fill in your name and email address and select 'Research Roundup':
https://www.physoc.org/journals-and-media/membernews

EDITOR COMMENTS

Reviewing Editor:
The authors have satisfied all reviewer concerns.
